# Characterization of Gut Bacteria in Natural Populations of Sand Flies (*Diptera*: *Psychodidae*) from Endemic and Non-Endemic Areas of Leishmaniasis in Morocco

**DOI:** 10.3390/microorganisms13102279

**Published:** 2025-09-30

**Authors:** Mohamed Daoudi, Abdelkrim Outammassine, El Mahdi Redouane, Souad Loqman, Mohamed Hafidi, Ali Boumezzough, Martin Olivier, Samia Boussaa, Momar Ndao

**Affiliations:** 1 Infectious Diseases and Immunity in Global Health Program, Research Institute of the McGill University Health Centre, Montreal, QC H4A 3J1, Canada; mohamed.daoudi@mail.mcgill.ca (M.D.); martin.olivier@mcgill.ca (M.O.); 2 Department of Microbiology and Immunology, McGill University, Montreal, QC H3A 2B4, Canada; 3 Laboratory of Microbiology and Virology, Faculty of Medicine and Pharmacy, Cadi Ayyad University, Marrakech 40080, Morocco; abdelkrim.outammassine@gmail.com (A.O.); 4 Laboratory of Water, Microbial Biotechnologies and Natural Resources Sustainability (AQUABIOTECH), Department of Biology, Faculty of Sciences Semlalia, Cadi Ayyad University, Marrakech 40000, Morocco; 5 Microbial Biotechnologies, Agrosciences and Environment Laboratory, Faculty of Sciences Semlalia, Cadi Ayyad University, Marrakech 40080, Morocco; hafidi@uca.ac.ma (M.H.); aboumezzough@gmail.com (A.B.); 6 Division of Experimental Medicine, McGill University, Montreal, QC H3A 2B4, Canada; 7 Higher Institute of Nursing Professions and Health Techniques, Ministry of Health and Social Protection, Rabat 10020, Morocco; 8 National Reference Centre for Parasitology, Research Institute of the McGill University Health Centre, Montreal, QC H4A 3J1, Canada

**Keywords:** gut bacteria, sand fly, MALDI-TOF, *Leishmania*, Morocco

## Abstract

Leishmaniasis is a vector-borne parasitic disease caused by *Leishmania* spp., transmitted to humans by phlebotomine sand flies. The development of *Leishmania* into infective metacyclic promastigotes occurs within the sand fly gut, where the bacterial microbiota plays a pivotal role in parasite development and transmission dynamics. This study aimed to characterize the gut bacterial composition of phlebotomine sand flies collected from both endemic (Lalla Aaziza) and non-endemic (Marrakech) regions of leishmaniasis in Morocco. We investigated the microbiota of *Phlebotomus papatasi*, *P. sergenti*, *P. perniciosus*, and *P. longicuspis*, all proven vectors of cutaneous and visceral leishmaniasis in the Old World, including Morocco, as well as *Sergentomyia minuta*, a potential vector in the Mediterranean basin. Gut bacteria were isolated using conventional microbiological techniques and identified by MALDI-TOF mass spectrometry. Fifteen bacterial strains from three phyla were identified, with *Bacillus pumilus* being the most frequently detected species. Significant differences in colony-forming unit (CFU) counts and bacterial richness were observed between sand fly species and collection sites. Notably, *Bacillus simplex* (in *P. papatasi*), *Nocardia ignorata* (in *P. sergenti*), and *Serratia* spp. (in *P. longicuspis*) were identified for the first time in these vectors. This study is the first to investigate the gut bacterial composition of sand flies in Morocco, revealing species and locality-dependent differences in microbial communities. The predominance of *Bacillus* spp., particularly *B. pumilus*, suggests a potentially influential role in sand fly physiology and vector competence. Furthermore, the novel detection of *B. simplex*, *N. ignorata*, and *Serratia* spp. underscores previously unrecognized microbial associations that warrant further investigation. These findings provide a critical baseline for future studies exploring the microbiota-mediated modulation of sand fly–*Leishmania* interactions.

## 1. Introduction

Phlebotomine sand flies (*Diptera*: *Psychodidae*) are the exclusive natural vectors of leishmaniasis, a group of parasitic diseases caused by protozoan *Kinetoplastid* flagellates belonging to the genus *Leishmania*. Leishmaniasis affects between 12 and 15 million people in many countries located in the Mediterranean, tropical and subtropical regions, with around 350 million people at risk [1]. In Morocco, leishmaniasis remains a significant public health concern in both endemic and emerging regions. Cutaneous leishmaniasis (CL) caused by *L. tropica* has notably expanded since the 1980s across central and arid areas, becoming the most prevalent form of the disease [2]. Between 1997 and 2018, over 80,000 cases of human leishmaniasis were reported nationally [3]. In the northern Tangier–Tétouan–Al Hoceima region, 1255 cases were recorded, representing about 1.56% of the national burden, with *Leishmania infantum* responsible for most infections [4]. Visceral leishmaniasis (VL) remains endemic in northern Morocco, with approximately 100 cases reported annually, mainly affecting children under five years old [5]. The disease also has serious significant social and psychological consequences, as CL often results in permanent scarring and associated stigma [6]. Sand flies bear different microorganisms in their body and in their gut [7]. The sand fly gut hosts many microorganisms, including bacteria. Several studies have focused on the microbial community of both *Phlebotomus* and *Lutzomyia* species [8,9,10]. Bacterial symbionts significantly control some aspects of the physiology of their host [11]. Characterizing studies on insects’ gut microbiota and their role have been well investigated in recent years [12,13]. Studies focused on diverse mosquito species microbiota, including a potential influence on their vector competence [14,15]. Microorganisms, including bacteria, fungi, and protozoan parasites, can modulate insect vectorial capacity [16,17,18,19]. Furthermore, some investigations have shown a significant impact of the mosquito midgut microbiota in defense against malaria parasites [20]. However, several articles have been published on the effect of sand fly microflora on biology and sand fly–pathogen interaction. Volf et al. (2002) showed that *Phlebotomus dubosqui* had an associated gut bacterial cluster immediately after adult emergence [17]. Moreover, vector gut microbiota influences the development of virulent parasites, as demonstrated in *L. infantum* infection [21]. Some investigations on the midgut bacterial flora have been conducted on *Lutzomyia longipalpis*, *Phlebotomus papatasi*, *P. perniciosus, P. tobbi*, *P. argentipes*, *P. duboscqi* and *Sergentomyia* spp. [8,22,23,24]. Studies have shown that *Phlebotomus papatasi* harbors a low-diversity gut microbiota dominated by *Enterobacteriaceae* (e.g., *Pantoea*, *Enterobacter*, *Serratia*), which may influence *Leishmania major* development [25]. In contrast, *P. perniciosus* exhibits a richer and seasonally variable microbiota, including *Ochrobactrum*, *Serratia*, and *Bacillus*, potentially impacting *L. infantum* transmission [24]. A recent study by Cecilio et al. (2025) [26] demonstrated that the bacterium *Delftia tsuruhatensis* TC1, when introduced into the gut of *Phlebotomus duboscqi* sand flies, disrupts the development of *L. major* parasites. This disruption is attributed to gut dysbiosis induced by the bacterium, leading to reduced vector competence and decreased transmission potential [26]. These findings suggest that gut microbial composition may modulate vector competence and serve as an infection biomarker in Moroccan populations.

Our study aimed to examine the diversity of gut bacterial community colonization in field-caught sand flies through a culture-dependent method and to discuss its potential implications in sand fly vectorial competence.

## 2. Materials and Methods

### 2.1. Sand Fly Collection, Species Identification, and Gut Dissection

**Figure 1:**
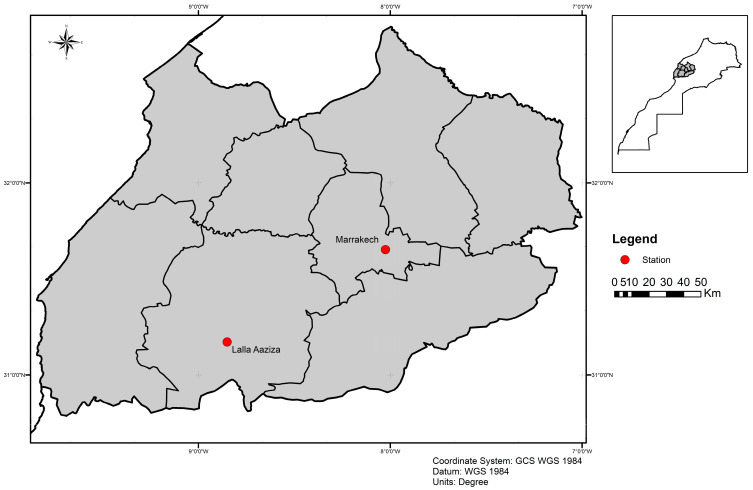
Map with locations of surveyed sites (red circle).

Sampling was carried out in Marrakech city (MA) (31°39′ N 8°01′ W) and Lalla Aaziza (LA) in Chichaoua province (31°04′ N 8°42′ W), located in the central region of Morocco (Figure 1). Lalla Aaziza is well-known as an active focus of cutaneous leishmaniasis due to *Leishmania tropica* in Morocco. In contrast, Marrakech is considered free of leishmaniasis [27].

At each site, six CDC miniature light traps were deployed and placed inside human dwellings. Traps were set in fixed indoor and outdoor locations in randomly selected habitations within the two study locations. Sand flies were collected throughout the activity season, between May and November 2018, yielding a total of 307 specimens. Sampling was conducted monthly over a seven-month period. Traps were deployed before sunset, sterilized with ethanol prior to use, and retrieved the following morning.

In this step, we followed the protocol described by Akhoundi et al. [28]. Each female sand fly was placed in a 1.5 mL microtube containing 30 µL of absolute ethanol and gently vortexed for 1 min to remove surface bacterial contaminants. Using sterile entomological needles, specimens were transferred into new sterile microtubes containing 30 µL of phosphate-buffered saline (PBS) to eliminate residual ethanol. Sand flies were then dissected under a stereomicroscope on a sterile slide with a drop of PBS. The head was removed, and the midgut was isolated, transferred into 30 µL of PBS into a new sterile microtube, and homogenized using a sterile glass pestle. Finally, 30 µL of the resulting suspension was used for bacterial analysis [28].

The genitalia and head of the sand flies were mounted on a slide, and species identification was performed morphologically using Boussaa’s identification key [29].

### 2.2. Bacterial Isolation and Identification

Bacterial identification was performed individually for each sample using standard microbiological techniques, including cultivation, isolation, and identification. From each 30 µL bacterial suspension, 15 µL was directly plated onto Plate Count Agar (PCA) medium (Cat. No. 105463, E. Merck Co., Darmstadt, Germany) to assess colony-forming units (CFU). The remaining 15 µL was inoculated into Brain Heart Infusion (BHI) broth and incubated at 37 °C for 24 h. PCA plates were incubated at 35 °C for 48 h, and the total number of colonies was counted. The CFU for each sample was calculated based on colony counts and dilution factors.

Following incubation in BHI, enriched cultures were plated onto various selective and differential media, including MacConkey Agar, Mannitol Salt Agar, Bile Esculin Agar, Cetrimide Agar, Trypticase Soy Agar, and Blood Agar supplemented with Amphotericin B (2 µg/mL). All plates were incubated at 37 °C for 24–48 h [28]. Isolated colonies were then analyzed by macroscopic (colony size, shape, edge, and optical properties), microscopic (Gram staining using the HiMedia Gram-stain Kit, HiMedia Laboratories Pvt. Ltd., Mumbai, Maharashtra, India), and biochemical methods (Catalase, Oxidase, DNase tests, and API 20E system). Final species-level identification was confirmed using matrix-assisted laser desorption/ionization time-of-flight mass spectrometry (MALDI-TOF MS), following established protocols and the manufacturer’s recommendations [30].

### 2.3. MALDI-TOF Assay

We employed Matrix-Assisted Laser Desorption/Ionization Time-of-Flight Mass Spectrometry (MALDI-TOF MS) to identify bacterial strains isolated from sand flies. A single colony of each bacterial strain was directly smeared onto a designated spot on a metal target plate and allowed to air-dry. Subsequently, 1 µL of a saturated solution of α-cyano-4-hydroxycinnamic acid (CHCA) matrix was added to each spot, and the plate was again air-dried to facilitate co-crystallization of the matrix with bacterial proteins. The prepared target plate was then introduced into the MALDI-TOF MS instrument (Bruker Daltonics, Bremen, Germany), where it was subjected to laser irradiation under vacuum conditions. The ionized proteins were accelerated in an electric field, and their time-of-flight (TOF) to the detector was measured, generating a mass spectrum for each isolate. These spectra were analyzed using Biotyper 2.0 software (Bruker Daltonics), which compared them against a reference database to identify bacterial species based on their unique protein fingerprints (see Supplementary Material).

### 2.4. Statistical Analysis

Two-way analysis of variance (ANOVA) was performed based on colony counts and bacterial species richness across localities, using SPSS software ver. 18 to detect statistical differences in CFU and bacterial populations isolated from sand fly guts. Cytoscape software (version 3.9.1; http://www.cytoscape.org; accessed on 2 September 2020) was employed to construct and visualize networks of associations between sand fly species and their gut bacteria. In these networks, nodes represented either a bacterial species or a sand fly host, while edges indicated confirmed isolation links. Graphical representations and statistical analyses were generated using Microsoft Excel and GraphPad Prism ver. 10.2.3.

## 3. Results

A total of 175 specimens belonging to 7 species were collected in Lalla Aaziza (LA), *Phlebotomus sergenti* (45%), *P. perniciosus* (15%), *P. longicuspis* (11%), *P. papatasi* (9%), *Sergentomyia minuta* (10%), *S. fallax* (6%), and *S. dreyfussi* (4%), with a sex ratio of 0.8. In Marrakech (MA), 134 specimens representing five species were captured: *Phlebotomus papatasi* (40%), *P. sergenti* (18%), *P. longicuspis* (6%), *S. minuta* (23%), and *S. fallax* (13%), with a sex ratio of 0.6.

**Figure 2:**
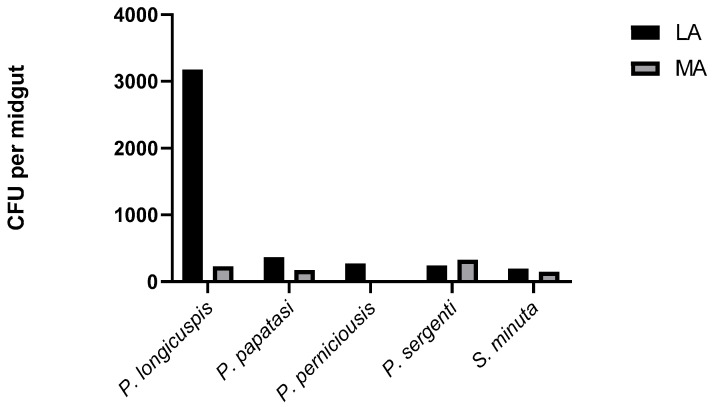
Mean CFU number of bacteria in the midgut of five sand fly species from Lalla Aaziza (LA) and Marrakech (MA) regions in the central area of Morocco.

**Figure 3:**
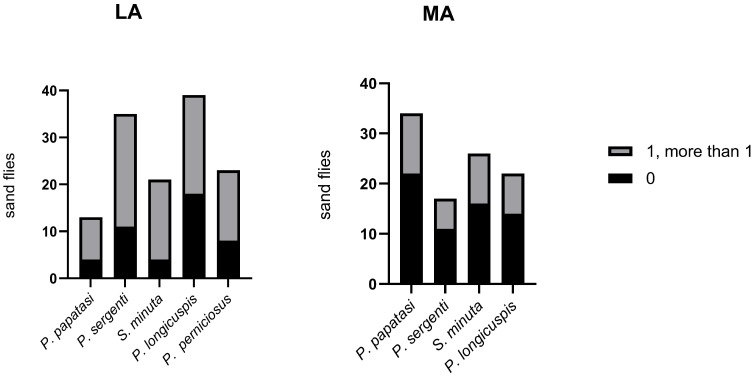
Number of specimens carrying no, one, or more bacterial strains on their gut in the Lalla Aaziza (LA) and Marrakech (MA) regions in the central area of Morocco.

Using a culture-dependent methodology, we successfully isolated the bacteria colonizing the midgut of sand flies. Two-way ANOVA revealed a statistically significant interaction between sand fly species and locality (*p* = 0.033). At the species level, significant differences in CFU counts were observed for *P. papatasi* (*p* < 0.05) and *P. longicuspis* (*p* < 0.05). In contrast, no significant differences were detected for *P. sergenti* and *S. minuta*. The mean number of colonies per midgut varied among species, with *P. longicuspis* from LA showing the highest CFU (3.1 × 10^2^) (Figure 2). The distribution of sand flies carrying no bacteria, a single bacterium, or multiple bacteria also varied among species (Figure 3). Overall, bacterial infection rates appeared higher in LA compared to MA. However, this difference could not be statistically assessed due to unequal sample sizes between localities (Figure 3).

MALDI-TOF identification of bacterial species allowed us to detect fifteen different strains belonging to three phyla (*Firmicutes*, *Proteobacteria*, and *Actinobacteria*) in five sand fly species (*P. sergenti*, *P. papatasi*, *P. longicuspis*, *P. perniciosus*, and *S. minuta*) from the Lalla Aaziza and Marrakech localities. Among these phyla, *Firmicutes* was the most dominant, followed by *Proteobacteria* and *Actinobacteria* (Table 1).

Table 2 shows that bacterial species richness varies according to both locality and sand fly species. ANOVA revealed significant differences between localities and sand fly species for *P. papatasi* and *P. sergenti* (*p* < 0.05). Notably, *P. sergenti* exhibited the highest bacterial richness among the five species studied (7 bacterial species, BS) (Table 2).

Of the fifteen bacterial strains identified, seven were Gram-negative and eight were Gram-positive. Members of the genus *Bacillus* were the most frequently isolated, with *Bacillus pumilus*, *Bacillus subtilis*, *Bacillus licheniformis*, and *Lysinibacillus fusiformis* being the most common species (Table 1). These taxa also represented the most abundant midgut colonizers across the four sand fly species examined from both localities (Table 3). Results obtained using culture-dependent methods are summarized in Table 3.

At the species level, bacterial composition differed between the two localities (Table 2 and Table 3). In *P. sergenti*, the gut bacterial profile from Lalla Aaziza consisted of *B. pumilus* (38.5%), *Staphylococcus hominis* (7.7%), *Staphylococcus* spp. (7.7%), *Staphylococcus aureus* (15.4%), *Escherichia coli* (15.4%), *Morganella morganii* (7.7%), and *Pseudomonas aeruginosa* (7.7%). In contrast, specimens from Marrakech harbored *B. pumilus* (33%), *B. subtilis* (16.6%), *Bacillus* sp. (16.6%), *E. coli* (16.6%), and *Nocardia ignorata* (16.6%).

For *P. longicuspis*, the gut composition in Lalla Aaziza included *B. pumilus* (71.4%), *S. aureus* (14.6%), and *L. fusiformis* (14%). In Marrakech, isolates included *B. pumilus* (40%), *B. subtilis* (20%), *E. coli* (20%), and *Serratia* spp. (20%) (Table 3).

In *P. papatasi*, bacteria isolated from LA included *B. pumilus* (50%) and *L. fusiformis* (50%), while in MA, the gut contained *B. pumilus* (47.4%), *B. subtilis* (14.2%), *Bacillus simplex* (14.2%), and *Burkholderia fungorum* (14.2%) (Table 3). From *S. minuta*, *B. pumilus* (50%) and *B. licheniformis* (50%) were isolated in LA, whereas *B. pumilus* (75%) and *B. subtilis* (25%) were found in MA (Table 3). In *P. perniciosus* from LA, the gut microbiota consisted of *B. subtilis* (60%), *S. hominis* (20%), and *S. aureus* (20%) (Table 3).

A comparison of taxonomic composition between the two localities at the species level revealed 11 bacterial species in Marrakech and 8 in Lalla Aaziza. Interestingly, five species *B. subtilis*, *B. simplex*, *Serratia* spp., *Burkholderia fungorum*, *N. ignorata*, and *B. licheniformis* were found exclusively in Marrakech. Notably, *B. simplex* was detected for the first time in *P. papatasi*. Similarly, *N. ignorata* was reported for the first time in *P. sergenti* and *Serratia* spp. in *P. longicuspis*.

**Figure 4:**
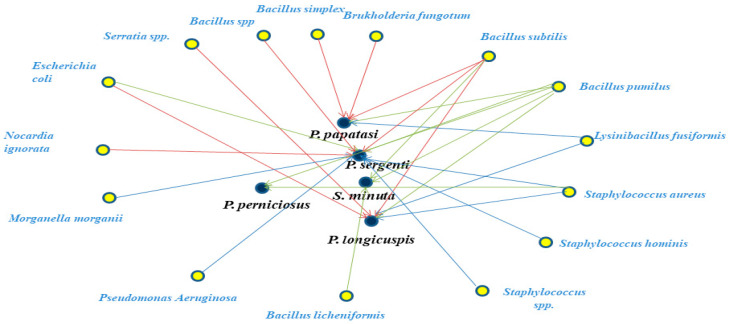
Network analysis showing the shared bacteria species found in natural populations of sand fly species including *P. papatasi*, *P. sergenti*, *P. longicuspis*, *P. perniciosus*, and *S. minuta* originating from the Lalla Aaziza and Marrakech regions in Morocco.

Overall, Gram-positive bacteria were more prevalent across the sand fly species examined, except in *P. longicuspis*, where Gram-negative bacteria were dominant. Network analysis further illustrated the associations between sand fly species and the bacterial taxa isolated in this study (Figure 4).

## 4. Discussion

Morocco is considered one of the countries where leishmaniasis is a major public health concern, and sand fly species exist within a significant geographical range [31,32]. The genus *Phlebotomus* includes the vectors of leishmaniasis in Morocco: *Phlebotomus papatasi*, the proven vector of zoonotic cutaneous leishmaniasis due to *L. major* [33]; *P. ariasi*, *P. longicuspis* and *P. perniciosus*, vectors of visceral leishmaniasis due to *L. infantum* [34]; and *P. sergenti*, the proven vector of anthroponotic cutaneous leishmaniasis caused by *L. tropica* [35].

Sand fly populations in the Mediterranean region, including Morocco, are recognized vectors of both *Leishmania* spp. and phleboviruses [36]; however, their vectorial competence, particularly regarding bacterial associations, remains underexplored. To investigate the bacterial composition of natural sand fly populations in Morocco, field studies were conducted in two central regions: Lalla Aaziza, an active focus of cutaneous leishmaniasis caused by *L. tropica* [37] with occasional cases of visceral leishmaniasis due to *L. infantum* [38], and Marrakech, which is considered a non-endemic area for leishmaniasis [39].

Sand flies are the only proven vectors of *Leishmania* species for humans [40]. Their vectorial capacity can be significantly influenced by symbiotic microorganisms [41,42,43], with bacteria representing promising targets for potential disease control strategies [44]. Understanding sand fly–bacteria–*Leishmania* interactions may provide new approaches for vector control and reduction in transmission. We therefore evaluated the bacterial composition of Moroccan phlebotomine sand flies and described the abundance of gut bacteria in different species and localities using a culture-dependent method and MALDI-TOF identification.

This identification technique has shown high potential for microbial identification in clinical and food sectors, as it is fast, easy to handle, and highly discriminatory down to the strain level [45]. Our results identified the gut bacterial flora of sand flies, which may contribute to better knowledge of their microbiota and to the development of new biological tools for disease control [46]. Several studies on the gut microbiome of sand flies in South America have applied classical molecular ecology techniques such as DGGE and 16S rRNA sequencing [10,18]. In Morocco, only one study has examined the intestinal microbiome of sand flies (*P. papatasi* compared to *P. duboscqi*) using DNA sequencing [47]. This is the first study to use a culture-dependent method coupled with MALDI-TOF to investigate the gut microbiota of Moroccan sand flies.

Our study on the gut flora of wild-caught sand flies (*P. papatasi*, *P. sergenti*, *P. longicuspis*, *P. perniciosus*, and *S. minuta*) demonstrated the presence of a wide diversity of bacterial strains belonging to the phyla Firmicutes (8 strains), Proteobacteria (6 strains), and Actinobacteria (1 strain). This result confirms previous investigations on sand fly gut microbiota, showing that Proteobacteria and Firmicutes are the predominant phyla [16,48,49]. Several studies carried out on the gut of natural populations or laboratory-reared sand flies have revealed the presence of diverse bacterial genera, including *Bacillus*, *Staphylococcus*, *Burkholderia*, *Escherichia*, *Pseudomonas*, *Serratia*, *Cellulomonas*, *Chloroflexi*, *Citrobacter*, *Microbacterium*, *Enterobacter*, *Flavimonas*, *Gordonia*, *Klebsiella*, *Maltophila*, *Brevibacterium*, *Micrococcus*, *Morganella*, *Ochrobactrum*, *Oligella*, *Pantoea*, *Shigella*, *Sphingobacterium*, *Stenotrophomonas*, *Streptococcus*, *Acinetobacter*, and *Weeksella* [8,24,25]. The genera *Bacillus*, *Staphylococcus*, *Burkholderia*, *Escherichia*, *Pseudomonas*, *Serratia*, and *Morganella* were also detected in our samples. We observed that variations in sand fly gut microbiota between species and localities (Figure 2 and Table 2) may be explained by the influence of microhabitats and physicochemical properties such as pH, oxygen availability in the insect gut, and food sources [50]. In the present study, Gram-positive strains (8) were isolated more frequently than Gram-negative strains (7). Additionally, in *P. longicuspis*, Gram-negative bacteria were the most prevalent strains, particularly in the Marrakech locality. Previous investigations have reported that the presence and prevalence of Gram-negative bacteria can completely or partially influence pathogen development in the insect midgut [25]. Moreover, the high prevalence of microbial infections in sand fly guts could negatively affect *Leishmania* transmission in endemic areas [51].

Notable variations in gut bacterial composition were observed among sand fly species across the two study localities. Among the dominant strains, *Bacillus pumilus* (Gram-positive) was detected in all sand fly species except *P. perniciosus*. The widespread occurrence and high abundance of *B. pumilus* may be attributed to its resistance to extreme environmental conditions, as previously reported by Kempf et al. (2005) [52]. Interestingly, members of the *Bacillus* genus exhibited host specificity and geographical variation. For example, *Bacillus subtilis* was exclusively isolated from sand flies collected in Marrakech (*P. sergenti*, *P. papatasi*, *P. longicuspis*, and *S. minuta*), whereas in Lalla Aaziza, it was only found in *P. perniciosus*. Although *B. subtilis* is considered non-pathogenic to humans [53], it is widely used in agriculture as a biofertilizer [54] and has been reported to possess probiotic properties [55,56]. These findings suggest that both environmental factors and host species influence the composition and distribution of sand fly gut microbiota, with *Bacillus* species showing ecological and biological adaptability. The isolation of *B. subtilis* from *P. papatasi* highlights its potential as a candidate for paratransgenic approaches, although other genera were detected less frequently. *P. sergenti*, *P. papatasi*, *P. perniciosus*, and *P. longicuspis* are well-known vectors of *Leishmania* spp. in Morocco and elsewhere [34,35]. *S. minuta* could also be a potential vector of *L. infantum* and *L. tropica* in Morocco [57].

The high bacterial diversity observed in sand flies may be linked to vectorial competence. For example, the gut microbiota of *Lutzomyia longipalpis* (the vector of VL in the New World) has been shown to play an important role in *Leishmania* development [21]. A study by Fraihi et al. (2017) reported that seasonal variation in *P. perniciosus* gut microbiota was associated with *L. infantum* transmission periods [24]. The identification of *Serratia* spp. and *B. subtilis* in sand flies in our study suggests these species may be suitable candidates for paratransgenic strategies [14,19,58]. Interestingly, *B. subtilis* was identified only in Marrakech, a non-endemic area of leishmaniasis in Morocco. This bacterium has already been used in paratransgenic mosquitoes and sand flies [55,56].

Some bacteria detected, such as *Escherichia coli*, are commonly associated with humans. Their presence may reflect environmental contamination from human or animal waste, or contact with contaminated soil, rather than stable colonization of sand fly guts. Although our study provides valuable insights into the bacterial communities of sand flies from endemic and non-endemic areas of Morocco, some limitations should be noted. Sampling was conducted monthly from May to September, covering only the peak sand fly activity season, so seasonal variations outside this period were not assessed. Moreover, the use of culture-dependent bacterial identification may underestimate the full microbial diversity present. These factors should be considered when interpreting the potential role of sand fly microbiota in vectorial competence.

## 5. Conclusions

This study provides the first characterization of gut bacterial communities in Moroccan sand flies from both endemic and non-endemic regions of leishmaniasis. A total of fifteen bacterial strains from three phyla were identified, with *Bacillus pumilus* being the most frequently detected species. Notably, *Bacillus simplex* (in *P. papatasi*), *Nocardia ignorata* (in *P. sergenti*), and *Serratia* spp. (in *P. longicuspis*) were reported for the first time in these vectors, highlighting previously unrecognized microbial associations. Significant differences in bacterial richness and colony-forming units were observed between sand fly species and localities, underscoring species and environment-dependent variation in gut microbiota. These findings provide a critical baseline for future studies on the role of gut bacteria in sand fly physiology and vector competence, and they open promising avenues for paratransgenic strategies to control leishmaniasis transmission in Morocco.

## Figures and Tables

**Table 1:** Assignment of bacterial strains and corresponding sand fly species.

Bacterial Strains	Gram-Stain	Identifying NCBI	Phylum	Sand Fly Species
*Bacillus pumilus*	Gram+	130148166	Firmicutes	*P. sergenti* *P. papatasi* *S. minuta* *P. longicuspis* *P. perniciosus*
*Bacillus simplex*	Gram−	133055080		*P. papatasi*
*Bacillus subtilis*	Gram+	133993714		*S. minuta* *P. papatasi* *P. sergenti* *P. longicuspis*
*Bacillus licheniformis*	Gram+	1402		*S. minuta*
*Bacillus* sp.	Gram+	1409		*P. sergenti*
*Lysinibacillus fusiformis*	Gram+	28031		*P. papatasi* *P. longicuspis*
*Staphylococcus aureus*	Gram+	703339		*P. sergenti* *P. perniciosus* *P. longicuspis*
*Staphylococcus hominis*	Gram−	1290		*P. sergenti* *P. perniciosus*
*Staphylococcus* spp.	Gram+	29387		*P. sergenti*
*Brukholderia fungorum*	Gram−	1218077	Proteobacteria	*P. papatasi*
*Morganella morganii*	Gram−	582		*P. sergenti*
*Pseudomonas aeruginosa*	Gram−	287		*P. sergenti*
*Escherichia coli*	Gram−	562		*P. longicuspis*
				*P. sergenti*
*Serratia* spp.	Gram−	616		*P. longicuspis*
*Nocardia ignorata*	Gram+	145285	Actinobacteria	*P. sergenti*

**Table 2:** Details of bacterial richness in the midgut of sand fly species from the Lalla Aaziza and Marrakech regions in the central area of Morocco.

Location	*P. papatasi*		*P. longicuspis*		*P. sergenti*		*P. perniciosus*		*S. minuta*		Total
	n	bspp	n	bspr	n	bspp	n	bspp	n	bspp	n
Lalla Aaziza	9	2	21	3	24	7	15	3	17	2	89
Marrakech	22	4	11	4	14	5	*	*	16	2	71
Total	31		32		38		15		33		160

Abbreviations: n number of sand flies, bspr bacterial specific richness, * absence of species in this locality.

**Table 3:** Gut bacterial composition of sand fly species from endemic (Lalla Aaziza) and non-endemic (Marrakech) regions of Morocco.

Sand Fly Species	Locality	Bacterial Species	Relative Abundance (%)
*P. sergenti*	Lalla Aaziza	*Bacillus pumilus*	38.5
		*Staphylococcus hominis*	7.7
		*Staphylococcus* spp.	7.7
		*Staphylococcus aureus*	15.4
		*Escherichia coli*	15.4
		*Morganella morganii*	7.7
		*Pseudomonas aeruginosa*	7.7
	Marrakech	*Bacillus pumilus*	33
		*Bacillus subtilis*	16.6
		*Bacillus* sp.	16.6
		*Escherichia coli*	16.6
		*Nocardia ignorata*	16.6
*P. longicuspis*	Lalla Aaziza	*Bacillus pumilus*	71.4
		*Staphylococcus aureus*	14.6
		*Lysinibacillus fusiformis*	14
	Marrakech	*Bacillus pumilus*	40
		*Bacillus subtilis*	20
		*Escherichia coli*	20
		*Serratia* spp.	20
*P. papatasi*	Lalla Aaziza	*Bacillus pumilus*	50
		*Lysinibacillus fusiformis*	50
	Marrakech	*Bacillus pumilus*	47.4
		*Bacillus subtilis*	14.2
		*Bacillus simplex*	14.2
		*Brukholderia fungorum*	14.2
*S. minuta*	Lalla Aaziza	*Bacillus pumilus*	50
		*Bacillus licheniformis*	50
	Marrakech	*Bacillus pumilus*	75
		*Bacillus subtilis*	25
*P. perniciosus*	Lalla Aaziza	*Bacillus subtilis*	60
		*Staphylococcus hominis*	20
		*Staphylococcus aureus*	20

## Data Availability

The original contributions presented in this study are included in the article/Supplementary Material. Further inquiries can be directed to the corresponding author.

## Supplementary Materials

The following supporting information can be downloaded at: https://www.mdpi.com/article/10.3390/microorganisms13102279/s1. S1: Bruker Daltonik MALDI Biotyper Identification Results 1; S2: Bruker Daltonik MALDI Biotyper Identification Results 2.

## Abbreviations

MALDI-TOF	Matrix-Assisted Laser Desorption Ionization—Time of Flight
P	*Phlebotomus*
S	*Sergentomyia*
L	*Leishmania*
DGGE	Denaturing Gradient Gel Electrophoresis
CFUs	Colony-Forming Units
LA	Lala Laaziza
MA	Marrakech
PBS	Phosphate-Buffered Saline
