# Supplementary material for: Characterization of Gut Bacteria in Natural Populations of Sand Flies (Diptera: Psychodidae) from Endemic and Non-Endemic Areas of Leishmaniasis in Morocco
Source: Microorganisms. 2025 Sep 30;13(10):2279. doi: 10.3390/microorganisms13102279 (PMC12565922; doi:10.3390/microorganisms13102279)
Supplement: Supplementary file 1 [file microorganisms-13-02279-s001.zip › microorganisms-3810545 S1.pdf]

Bruker Daltonik MALDI Biotyper
Résultats d'identification

Détails du projet:

**Nom du projet:**	190710-1700-10102279
**Description du projet:**	DAS-02279-100719-1
**Auteur du projet:**	tof-user@FLEX-PC
**Date/Heure de création du projet:**	2019-07-10T17:04:20.582
**Nombre d'échantillons :**	96
**Type :**	Échantillon standard
**BTS-QC :**	pas existant
**Position BTS-QC :**	
**ID instrument :**	8269944.03603
**Version du serveur :**	4.1.80 (PYTH) 102 2017-08-226_04-55-52

Aperçu des résultats

Nom de l'échantillon	ID de l'échantillon	Organisme (meilleur candidat)	Score Valeur	Organisme (second candidat)	Score Valeur
A1 (-) (C)	A1 (standard)	**Aucune identification possible**	1.35	**Aucune identification possible**	1.33
A2 (+++) (A)	A2 (standard)	Bacillus pumilus	2.26	Bacillus pumilus	2.15
A3 (+++) (A)	A3 (standard)	Bacillus pumilus	2.23	Bacillus pumilus	2.20
A4 (+++) (A)	A4 (standard)	Bacillus pumilus	2.34	Bacillus pumilus	2.19
A5 (-) (C)	A5 (standard)	**Aucune identification possible**	1.40	**Aucune identification possible**	1.39
A6 (-) (C)	A6 (standard)	**Aucune identification possible**	1.41	**Aucune identification possible**	1.38

Aperçu des résultats -- suite page suivante

Aperçu des résultats -- suite de la page précédente					
**Nom de l'échantillon**	**ID de l'échantillon**	**Organisme (meilleur candidat)**	**Score Valeur**	**Organisme (second candidat)**	**Score Valeur**
A7 (+++) (A)	A7 (standard)	Bacillus pumilus	2.27	Bacillus pumilus	2.20
A8 (+++) (A)	A8 (standard)	Bacillus pumilus	2.19	Bacillus pumilus	2.16
A9 (+++) (A)	A9 (standard)	Bacillus pumilus	2.07	Bacillus pumilus	1.99
A10 (+++) (A)	A10 (standard)	Bacillus pumilus	2.30	Bacillus pumilus	2.18
A11 (+++) (A)	A11 (standard)	Bacillus pumilus	2.06	Bacillus pumilus	1.97
A12 (+++) (A)	A12 (standard)	Bacillus pumilus	2.09	Bacillus pumilus	1.98
B1 (+) (B)	B1 (standard)	Turicella otitidis	1.71	**Aucune identification possible**	1.64
B2 (+) (B)	B2 (standard)	Kocuria rhizophila	1.71	**Aucune identification possible**	1.68
B3 (-) (C)	B3 (standard)	**Aucune identification possible**	1.49	**Aucune identification possible**	1.16
B4 (-) (C)	B4 (standard)	**Aucune identification possible**	1.38	**Aucune identification possible**	1.28
B5 (+++) (A)	B5 (standard)	Bacillus pumilus	2.44	Bacillus pumilus	2.29
B6 (-) (C)	B6 (standard)	**Aucune identification possible**	1.52	**Aucune identification possible**	1.49
B7 (+++) (A)	B7 (standard)	Lysinibacillus fusiformis	2.16	Lysinibacillus fusiformis	2.13
B8 (+++) (A)	B8 (standard)	Lysinibacillus fusiformis	2.13	Lysinibacillus fusiformis	2.08
B9 (+++) (A)	B9 (standard)	Lysinibacillus fusiformis	2.13	Lysinibacillus fusiformis	2.13
B10 (+++) (A)	B10 (standard)	Lysinibacillus fusiformis	2.02	Lysinibacillus fusiformis	2.01
Aperçu des résultats -- suite page suivante					

Aperçu des résultats -- suite de la page précédente					
**Nom de l'échantillon**	**ID de l'échantillon**	**Organisme (meilleur candidat)**	**Score Valeur**	**Organisme (second candidat)**	**Score Valeur**
B11 (+++) (A)	B11 (standard)	Bacillus pumilus	2.03	Bacillus pumilus	1.98
B12 (-) (C)	B12 (standard)	**Aucune identification possible**	1.47	**Aucune identification possible**	1.39
C1 (+++) (A)	C1 (standard)	Bacillus pumilus	2.17	Bacillus pumilus	2.13
C2 (+++) (A)	C2 (standard)	Bacillus pumilus	2.09	Bacillus pumilus	1.99
C3 (+++) (A)	C3 (standard)	Bacillus pumilus	2.42	Bacillus pumilus	2.35
C4 (-) (C)	C4 (standard)	**Aucune identification possible**	1.42	**Aucune identification possible**	1.42
C5 (+++) (A)	C5 (standard)	Bacillus pumilus	2.11	Bacillus pumilus	1.98
C6 (+++) (A)	C6 (standard)	Bacillus pumilus	2.24	Bacillus pumilus	2.10
C7 (+) (B)	C7 (standard)	Staphylococcus hominis	1.93	Staphylococcus hominis	1.82
C8 (+) (B)	C8 (standard)	Staphylococcus hominis	1.93	Staphylococcus hominis	1.90
C9 (-) (C)	C9 (standard)	**Aucune identification possible**	1.50	**Aucune identification possible**	1.34
C10 (-) (C)	C10 (standard)	**Aucune identification possible**	1.23	**Aucune identification possible**	1.17
C11 (+++) (A)	C11 (standard)	Bacillus pumilus	2.14	Bacillus pumilus	2.11
C12 (-) (C)	C12 (standard)	**Aucune identification possible**	1.15	**Aucune identification possible**	1.10
D1 (+) (B)	D1 (standard)	Escherichia coli	1.95	Escherichia coli	1.89
D2 (+++) (A)	D2 (standard)	Escherichia coli	2.38	Escherichia coli	2.38
Aperçu des résultats -- suite page suivante					

Nom de l'échantillon	ID de l'échantillon	Organisme (meilleur candidat)	Score Valeur	Organisme (second candidat)	Score Valeur
Aperçu des résultats -- suite de la page précédente					
D3 (-) (C)	D3 (standard)	**Aucune identification possible**	1.56	**Aucune identification possible**	1.43
D4 (-) (C)	D4 (standard)	**Aucune identification possible**	1.46	**Aucune identification possible**	1.40
D5 (+) (B)	D5 (standard)	Bacillus simplex	1.97	Bacillus simplex	1.86
D6 (+) (B)	D6 (standard)	Bacillus simplex	1.91	**Aucune identification possible**	1.57
D7 (+++) (A)	D7 (standard)	Bacillus pumilus	2.42	Bacillus pumilus	2.40
D8 (+) (B)	D8 (standard)	Bacillus pumilus	1.75	Bacillus pumilus	1.73
D9 (+++) (A)	D9 (standard)	Bacillus pumilus	2.36	Bacillus pumilus	2.29
D10 (+++) (A)	D10 (standard)	Bacillus pumilus	2.23	Bacillus pumilus	2.16
D11 (-) (C)	D11 (standard)	**Aucune identification possible**	1.38	**Aucune identification possible**	1.33
D12 (+) (B)	D12 (standard)	Bacillus simplex	1.86	**Aucune identification possible**	1.53
E1 (+) (B)	E1 (standard)	Bacillus pumilus	1.99	Bacillus pumilus	1.92
E2 (+++) (A)	E2 (standard)	Bacillus pumilus	2.14	Bacillus pumilus	2.01
E3 (+++) (A)	E3 (standard)	Bacillus pumilus	2.31	Bacillus pumilus	1.99
E4 (+++) (A)	E4 (standard)	Bacillus pumilus	2.39	Bacillus pumilus	2.37
E5 (+++) (A)	E5 (standard)	Bacillus pumilus	2.31	Bacillus pumilus	2.27
E6 (+) (B)	E6 (standard)	Bacillus pumilus	1.75	Bacillus pumilus	1.72

Aperçu des résultats -- suite page suivante

Aperçu des résultats -- suite de la page précédente					
**Nom de l'échantillon**	**ID de l'échantillon**	**Organisme (meilleur candidat)**	**Score Valeur**	**Organisme (second candidat)**	**Score Valeur**
E7 (+) (B)	E7 (standard)	Bacillus pumilus	1.74	Bacillus pumilus	1.71
E8 (+++) (A)	E8 (standard)	Bacillus pumilus	2.40	Bacillus pumilus	2.36
E9 (+++) (A)	E9 (standard)	Bacillus pumilus	2.48	Bacillus pumilus	2.24
E10 (+++) (A)	E10 (standard)	Bacillus pumilus	2.32	Bacillus pumilus	2.25
E11 (-) (C)	E11 (standard)	**Aucune identification possible**	1.63	**Aucune identification possible**	1.61
E12 (+++) (A)	E12 (standard)	Bacillus pumilus	2.18	Bacillus pumilus	2.07
F1 (-) (C)	F1 (standard)	**Aucune identification possible**	1.49	**Aucune identification possible**	1.40
F2 (+) (B)	F2 (standard)	Bacillus pumilus	1.81	**Aucune identification possible**	1.54
F3 (+++) (A)	F3 (standard)	Bacillus pumilus	2.29	Bacillus pumilus	2.10
F4 (+) (B)	F4 (standard)	Bacillus pumilus	1.99	Bacillus pumilus	1.94
F5 (+++) (A)	F5 (standard)	Bacillus pumilus	2.13	Bacillus pumilus	2.04
F6 (-) (C)	F6 (standard)	**Aucune identification possible**	1.31	**Aucune identification possible**	1.31
F7 (+++) (A)	F7 (standard)	Bacillus pumilus	2.28	Bacillus pumilus	2.15
F8 (+) (B)	F8 (standard)	Bacillus simplex	1.80	**Aucune identification possible**	1.67
F9 (+++) (A)	F9 (standard)	Bacillus pumilus	2.37	Bacillus pumilus	2.21
F10 (+++) (A)	F10 (standard)	Bacillus pumilus	2.11	Bacillus pumilus	2.06
Aperçu des résultats -- suite page suivante					

Aperçu des résultats -- suite de la page précédente					
**Nom de l'échantillon**	**ID de l'échantillon**	**Organisme (meilleur candidat)**	**Score Valeur**	**Organisme (second candidat)**	**Score Valeur**
F11 (+) (B)	F11 (standard)	Bacillus pumilus	1.94	Bacillus pumilus	1.92
F12 (+++) (A)	F12 (standard)	Bacillus pumilus	2.16	Bacillus pumilus	2.14
G1 (+++) (A)	G1 (standard)	Bacillus pumilus	2.45	Bacillus pumilus	2.32
G2 (+++) (A)	G2 (standard)	Bacillus pumilus	2.23	Bacillus pumilus	2.11
G3 (+++) (A)	G3 (standard)	Bacillus pumilus	2.32	Bacillus pumilus	2.28
G4 (+++) (A)	G4 (standard)	Bacillus pumilus	2.26	Bacillus pumilus	2.23
G5 (-) (C)	G5 (standard)	**Aucune identification possible**	1.49	**Aucune identification possible**	1.43
G6 (-) (C)	G6 (standard)	**Aucune identification possible**	1.67	**Aucune identification possible**	1.49
G7 (+++) (A)	G7 (standard)	Bacillus pumilus	2.14	Bacillus pumilus	2.05
G8 (+) (B)	G8 (standard)	Bacillus pumilus	1.90	Bacillus pumilus	1.89
G9 (+++) (A)	G9 (standard)	Bacillus pumilus	2.21	Bacillus pumilus	2.05
G10 (+++) (A)	G10 (standard)	Bacillus pumilus	2.42	Bacillus pumilus	2.40
G11 (-) (C)	G11 (standard)	**Aucune identification possible**	1.46	**Aucune identification possible**	1.43
G12 (+++) (A)	G12 (standard)	Bacillus subtilis	2.23	Bacillus subtilis	2.22
H1 (+++) (A)	H1 (standard)	Bacillus subtilis	2.05	Bacillus subtilis	2.03
H2 (-) (C)	H2 (standard)	**Aucune identification possible**	1.33	**Aucune identification possible**	1.33
Aperçu des résultats -- suite page suivante					

Aperçu des résultats -- suite de la page précédente					
**Nom de l'échantillon**	**ID de l'échantillon**	**Organisme (meilleur candidat)**	**Score Valeur**	**Organisme (second candidat)**	**Score Valeur**
H3 (-) (C)	H3 (standard)	**Aucune identification possible**	1.34	**Aucune identification possible**	1.31
H4 (+) (B)	H4 (standard)	Bacillus subtilis	1.99	Bacillus subtilis	1.89
H5 (-) (C)	H5 (standard)	**Aucune identification possible**	1.46	**Aucune identification possible**	1.34
H6 (+) (B)	H6 (standard)	Bacillus licheniformis	1.70	**Aucune identification possible**	1.48
H7 (-) (C)	H7 (standard)	**Aucune identification possible**	1.53	**Aucune identification possible**	1.43
H8 (+++) (A)	H8 (standard)	Escherichia coli	2.36	Escherichia coli	2.31
H9 (+++) (A)	H9 (standard)	Escherichia coli	2.39	Escherichia coli	2.39
H10 (+++) (A)	H10 (standard)	Escherichia coli	2.33	Escherichia coli	2.27
H11 (-) (C)	H11 (standard)	**Aucune identification possible**	1.49	**Aucune identification possible**	1.32
H12 (-) (C)	H12 (standard)	**Aucune identification possible**	1.30	**Aucune identification possible**	1.23

Indication de référence

Profil de référence	Commentaire
Aeromonas encheleia CECT 4342T DSM	Les profils des espèces appartenant à ce genre sont très ressemblants: la différentiation des espèces est donc difficile.
Aeromonas hydrophila CECT 839T DSM	Les profils des espèces appartenant à ce genre sont très ressemblants: la différentiation des espèces est donc difficile.
Aeromonas veronii CECT 5761T DSM	Les profils des espèces appartenant à ce genre sont très ressemblants: la différentiation des espèces est donc difficile.
Bacillus cibi DSM 16189T DSM	La qualité des spectres (qui influe sur le score) dépend du degré de sporulation: Utilisez des colonies fraîches.
Bacillus cohnii DSM 6307T DSM	La qualité des spectres (qui influe sur le score) dépend du degré de sporulation: Utilisez des colonies fraîches.
Bacillus endophyticus DSM 13796T DSM	La qualité des spectres (qui influe sur le score) dépend du degré de sporulation: Utilisez des colonies fraîches.
Bacillus indicus DSM 15820T DSM	La qualité des spectres (qui influe sur le score) dépend du degré de sporulation: Utilisez des colonies fraîches.
Bacillus jeotgali DSM 18226T DSM	La qualité des spectres (qui influe sur le score) dépend du degré de sporulation: Utilisez des colonies fraîches.
Bacillus licheniformis CS 54_1 BRB	appartient au groupe Bacillus subtilis. La qualité des spectres (qui influe sur le score) dépend du degré de sporulation: Utilisez des colonies fraîches.
Bacillus megaterium DSM 32T DSM	La qualité des spectres (qui influe sur le score) dépend du degré de sporulation: Utilisez des colonies fraîches.
Bacillus mojavensis DSM 9205T DSM	appartient au groupe Bacillus subtilis. La qualité des spectres (qui influe sur le score) dépend du degré de sporulation: Utilisez des colonies fraîches.
Bacillus muralis DSM 16288T DSM	La qualité des spectres (qui influe sur le score) dépend du degré de sporulation: Utilisez des colonies fraîches.
Bacillus pseudomycoides DSM 12442T DSM	Bacillus anthracis, cereus, mycoides, pseudomycoides, thuringiensis et weihenstephanensis sont proches et appartiennent au groupe Bacillus cereus. En particulier les spectres de Bacillus cereus sont très similaires à ceux de Bacillus anthracis. Bacillus anthracis n’est pas inclus dans la base de données du MALDI Biotyper. Pour une différentiation une méthode adéquate doit être sélectionnée par un professionnel expérimenté. La qualité des spectres (score) dépende du degré de sporulation: utiliser des cultures fraîches.
Bacillus psychrosaccharolyticus DSM 6T DSM	La qualité des spectres (qui influe sur le score) dépend du degré de sporulation: Utilisez des colonies fraîches.

Indication de référence --suite page suivante

Indication de référence -- suite de la page précédente	
**Profil de référence**	**Commentaire**
Bacillus pumilus DSM 354 DSM	La qualité des spectres (qui influe sur le score) dépend du degré de sporulation: Utilisez des colonies fraîches.
Bacillus simplex CS 206_1aI BRB	La qualité des spectres (qui influe sur le score) dépend du degré de sporulation: Utilisez des colonies fraîches.
Bacillus simplex DSM 1321T DSM	La qualité des spectres (qui influe sur le score) dépend du degré de sporulation: Utilisez des colonies fraîches.
Bacillus sonorensis DSM 13779T DSM	appartient au groupe Bacillus subtilis. La qualité des spectres (qui influe sur le score) dépend du degré de sporulation: Utilisez des colonies fraîches.
Bacillus subtilis DSM 5552 DSM	appartient au groupe Bacillus subtilis. La qualité des spectres (qui influe sur le score) dépend du degré de sporulation: Utilisez des colonies fraîches.
Bacillus subtilis DSM 5611 DSM	appartient au groupe Bacillus subtilis. La qualité des spectres (qui influe sur le score) dépend du degré de sporulation: Utilisez des colonies fraîches.
Bacillus subtilis ssp spizizenii DSM 15029T DSM	appartient au groupe Bacillus subtilis. La qualité des spectres (qui influe sur le score) dépend du degré de sporulation: Utilisez des colonies fraîches.
Bacillus subtilis ssp subtilis CICC 23950 CICC	appartient au groupe Bacillus subtilis. La qualité des spectres (qui influe sur le score) dépend du degré de sporulation: Utilisez des colonies fraîches.
Bacillus subtilis ssp subtilis DSM 10T DSM	appartient au groupe Bacillus subtilis. La qualité des spectres (qui influe sur le score) dépend du degré de sporulation: Utilisez des colonies fraîches.
Bacillus subtilis ssp subtilis DSM 5660 DSM	appartient au groupe Bacillus subtilis. La qualité des spectres (qui influe sur le score) dépend du degré de sporulation: Utilisez des colonies fraîches.
Bacteroides vulgatus DSM 1447T DSM	Les espèces vulgatus/dorei du genre Bacteroides présentent des profils très similaires: il est donc difficile de distinguer ces espèces. Bacteroides dorei n’est pas inclus dans la base de données du MALDI Biotyper. Pour une différentiation une méthode adéquate doit être sélectionnée par un professionnel expérimenté.
Citrobacter freundii 13158_2 CHB	Les profils des espèces appartenant à ce genre sont très ressemblants: la différentiation des espèces est donc difficile.
Clostridium beijerinckii 1011_DSM 552 BOG	Les espèces beijerinckii / diolis du genre Clostridium présentent des profils très similaires : il est donc difficile de distinguer ces espèces.
Escherichia coli ATCC 25922 CHB	proche de Shigella / Escherichia fergusonii mais ne peut en être distingué avec certitude pour le moment
Escherichia coli ATCC 25922 THL	proche de Shigella / Escherichia fergusonii mais ne peut en être distingué avec certitude pour le moment
Escherichia coli DH5alpha BRL	proche de Shigella / Escherichia fergusonii mais ne peut en être distingué avec certitude pour le moment
Indication de référence --suite page suivante	

Indication de référence -- suite de la page précédente	
**Profil de référence**	**Commentaire**
Escherichia coli MB11464_1 CHB	proche de Shigella / Escherichia fergusonii mais ne peut en être distingué avec certitude pour le moment
Listeria seeligeri CCUG 27802 CCUG	Une Extraction doit être effectuée pour permettre l'identification fiable d'espèce.
Lysinibacillus boronitolerans DSM 17140T DSM	Les espèces boronitolerans / xylanilyticus du genre Lysinibacillus présentent des profils très similaires : il est donc difficile de distinguer ces espèces. La qualité des spectres (qui influe sur le score) dépend du degré de sporulation: Utilisez des colonies fraîches.
Lysinibacillus fusiformis DSM 2898T BRB	La qualité des spectres (qui influe sur le score) dépend du degré de sporulation: Utilisez des colonies fraîches.
Lysinibacillus fusiformis DSM 2898T DSM	La qualité des spectres (qui influe sur le score) dépend du degré de sporulation: Utilisez des colonies fraîches.
Lysinibacillus fusiformis DSM 493 DSM	La qualité des spectres (qui influe sur le score) dépend du degré de sporulation: Utilisez des colonies fraîches.
Lysinibacillus sphaericus DSM 2899 DSM	La qualité des spectres (qui influe sur le score) dépend du degré de sporulation: Utilisez des colonies fraîches.
Lysinibacillus xylanilyticus CICC 20858 CICC	Les espèces boronitolerans / xylanilyticus du genre Lysinibacillus présentent des profils très similaires : il est donc difficile de distinguer ces espèces. La qualité des spectres (qui influe sur le score) dépend du degré de sporulation: Utilisez des colonies fraîches.
Neisseria meningitidis 639 PGM	Les espèces non pathogènes de Neisseria pourraient être confondues avec les espèces Neisseria meningitidis. Pour une différentiation une méthode adéquate doit être sélectionnée par un professionnel expérimenté.
Pseudomonas veronii B561 UFL	appartient au groupe Pseudomonas fluorescens
Stenotrophomonas maltophilia 10942 CHB	appartient au ou est proche de groupe Stenotrophomonas maltophilia

Correspondances des scores

Valeur	Description	Symboles	Couleur
2.00 - 3.00	Identification avec une haute confiance	(+++)	vert
1.70 - 1.99	Identification avec une faible confiance	(+)	jaune
0.00 - 1.69	Aucune identification possible	(-)	rouge

Signification des indices de cohérence (A - C)

Catégorie	Description
(A)	**Haute cohérence :** Le meilleur résultat correspond à une identification avec une haute confiance. Le deuxième meilleur résultat est (1) une identification avec une haute confiance, dans laquelle l'espèce est identique au meilleur résultat, (2) une identification avec une faible confiance, dans laquelle le genre est identique au meilleur résultat, ou (3) une absence d'identification.
(B)	**Faible cohérence :** Les exigences pour un indice de cohérence élevé ne sont pas atteintes. Le meilleur résultat correspond à une identification avec une confiance haute ou faible. Le deuxième meilleur résultat est (1) une identification avec une confiance haute ou faible, dans laquelle le genre est identique au meilleur résultat ou (2) une absence d'identification.
(C)	**Pas de cohérence :** Les exigences pour un indice de cohérence élevé ou faible ne sont pas atteintes.

Analyte 1

**Nom de l'échantillon:**	A1
**Description de l'échantillon:**	
**ID de l'échantillon:**	A1
**Date/Heure de création de l'échantillon:**	2019-07-10T17:04:20.599
**Type de l'échantillon:**	Échantillon standard
**Méthode de classification :**	MALDI Biotyper MSP Identification Standard Method 1.1
**Méthode de prétraitement :**	BioTyper Preprocessing Standard Method 1.2
**Méthode ACQ :**	D:\Methods\flexControlMethods\MBT_FC.par
**Horodatage ACQ :**	2019-07-10T17:05:04.563
**Méthode AutoXecute :**	MBT_AutoX_smart
**Bibliothèque de MSP utilisée:**	Culturomics / f8c211c3-71c5-471b-8a7e-7f6abca59bb9 / 2019-06-28T14:15:05.935, Timone / 29617d84-2a1e-4bf6-a13d-569eecb48f06 / 2018-04-19T13:24:29.884, BDAL / contains 7854 MSPs / e7ef41ca-b750-4d47-9a1c-6c26fa454356 / 2019-02-01T09:48:20.358

Classement (Qualité)	Profil de référence	Score Valeur	Identifiant NCBI
1 (-)	Arthrobacter gandavensis DSM 15046T DSM	1.35	169960
2 (-)	Aspergillus flavus 1081 PFM	1.33	5059
3 (-)	Arthrobacter koreensis DSM 16760T DSM	1.23	199136
4 (-)	Aeromonas hydrophila CECT 839T DSM	1.22	644
5 (-)	Lactobacillus fructivorans DSM 20203T DSM	1.21	1614
6 (-)	Cupriavidus necator B619 UFL	1.19	106590
7 (-)	Clostridium septicum 1026_NCTC 547T BOG	1.18	1504
8 (-)	Cryptococcus neoformans 29 PSB	1.18	5207
9 (-)	Gordonia rubripertincta B310 UFL	1.18	36822

Tableau des résultats pour analyte 1--suite page suivante

Tableau des résultats pour analyte 1 -- suite de la page précédente			
**Classement (Qualité)**	**Profil de référence**	**Score Valeur**	**Identifiant NCBI**
10<br>(-)	Streptococcus equi_ssp_zooepidemicus BKT17613_10 NVU	1.17	40041

Analyte 2

**Nom de l'échantillon:**	A2
**Description de l'échantillon:**	
**ID de l'échantillon:**	A2
**Date/Heure de création de l'échantillon:**	2019-07-10T17:04:20.601
**Type de l'échantillon:**	Échantillon standard
**Méthode de classification :**	MALDI Biotyper MSP Identification Standard Method 1.1
**Méthode de prétraitement :**	BioTyper Preprocessing Standard Method 1.2
**Méthode ACQ :**	D:\Methods\flexControlMethods\MBT_FC.par
**Horodatage ACQ :**	2019-07-10T17:05:26.050
**Méthode AutoXecute :**	MBT_AutoX_smart
**Bibliothèque de MSP utilisée:**	Culturomics / f8c211c3-71c5-471b-8a7e-7f6abca59bb9 / 2019-06-28T14:15:05.935, Timone / 29617d84-2a1e-4bf6-a13d-569eecb48f06 / 2018-04-19T13:24:29.884, BDAL / contains 7854 MSPs / e7ef41ca-b750-4d47-9a1c-6c26fa454356 / 2019-02-01T09:48:20.358

Classement (Qualité)	Profil de référence	Score Valeur	Identifiant NCBI
1 (+++)	Bacillus pumilus CSURP4085	2.26	130148166
2 (+++)	Bacillus pumilus CSURP8100	2.15	130148166
3 (+++)	Bacillus pumilus CSURP4226	2.10	130148166
4 (+++)	Bacillus pumilus CSURP4105	2.09	130148166
5 (+++)	Bacillus pumilus 10403329	2.00	133055080
6 (+)	Bacillus pumilus CSURP6343	1.91	130148166
7 (+)	Bacillus pumilus CSURP6343	1.91	130148166
8 (+)	Bacillus pumilus 10403607	1.88	133055080
9 (+)	Bacillus pumilus 10403206	1.85	133055080

Tableau des résultats pour analyte 2--suite page suivante

Tableau des résultats pour analyte 2 -- suite de la page précédente			
**Classement (Qualité)**	**Profil de référence**	**Score Valeur**	**Identifiant NCBI**
10<br>(+)	Bacillus pumilus 10403990	1.82	133055080

Analyte 3

**Nom de l'échantillon:**	A3
**Description de l'échantillon:**	
**ID de l'échantillon:**	A3
**Date/Heure de création de l'échantillon:**	2019-07-10T17:04:20.602
**Type de l'échantillon:**	Échantillon standard
**Méthode de classification :**	MALDI Biotyper MSP Identification Standard Method 1.1
**Méthode de prétraitement :**	BioTyper Preprocessing Standard Method 1.2
**Méthode ACQ :**	D:\Methods\flexControlMethods\MBT_FC.par
**Horodatage ACQ :**	2019-07-10T17:05:47.623
**Méthode AutoXecute :**	MBT_AutoX_smart
**Bibliothèque de MSP utilisée:**	Culturomics / f8c211c3-71c5-471b-8a7e-7f6abca59bb9 / 2019-06-28T14:15:05.935, Timone / 29617d84-2a1e-4bf6-a13d-569eecb48f06 / 2018-04-19T13:24:29.884, BDAL / contains 7854 MSPs / e7ef41ca-b750-4d47-9a1c-6c26fa454356 / 2019-02-01T09:48:20.358

Classement (Qualité)	Profil de référence	Score Valeur	Identifiant NCBI
1 (+++)	Bacillus pumilus CSURP8100	2.23	130148166
2 (+++)	Bacillus pumilus CSURP4226	2.20	130148166
3 (+++)	Bacillus pumilus CSURP4085	2.16	130148166
4 (+++)	Bacillus pumilus CSURP4105	2.11	130148166
5 (+++)	Bacillus pumilus 10403329	2.02	133055080
6 (+)	Bacillus pumilus 10403985	1.98	133055080
7 (+)	Bacillus pumilus 10403987	1.90	133055080
8 (+)	Bacillus pumilus 10403607	1.89	133055080
9 (+)	Bacillus pumilus 10403990	1.88	133055080

Tableau des résultats pour analyte 3--suite page suivante

Tableau des résultats pour analyte 3 -- suite de la page précédente			
**Classement (Qualité)**	**Profil de référence**	**Score Valeur**	**Identifiant NCBI**
10<br>(+)	Bacillus pumilus 10403751	1.84	133055080

Analyte 4

**Nom de l’échantillon:**	A4
**Description de l’échantillon:**	
**ID de l’échantillon:**	A4
**Date/Heure de création de l’échantillon:**	2019-07-10T17:04:20.604
**Type de l’échantillon:**	Échantillon standard
**Méthode de classification :**	MALDI Biotyper MSP Identification Standard Method 1.1
**Méthode de prétraitement :**	BioTyper Preprocessing Standard Method 1.2
**Méthode ACQ :**	D:\Methods\flexControlMethods\MBT_FC.par
**Horodatage ACQ :**	2019-07-10T17:06:09.281
**Méthode AutoXecute :**	MBT_AutoX_smart
**Bibliothèque de MSP utilisée:**	Culturomics / f8c211c3-71c5-471b-8a7e-7f6abca59bb9 / 2019-06-28T14:15:05.935, Timone / 29617d84-2a1e-4bf6-a13d-569eecb48f06 / 2018-04-19T13:24:29.884, BDAL / contains 7854 MSPs / e7ef41ca-b750-4d47-9a1c-6c26fa454356 / 2019-02-01T09:48:20.358

Classement (Qualité)	Profil de référence	Score Valeur	Identifiant NCBI
1 (+++)	Bacillus pumilus CSURP4226	2.34	130148166
2 (+++)	Bacillus pumilus CSURP8100	2.19	130148166
3 (+++)	Bacillus pumilus CSURP4085	2.15	130148166
4 (+++)	Bacillus pumilus CSURP4105	2.00	130148166
5 (+)	Bacillus pumilus CSURP6343	1.94	130148166
6 (+)	Bacillus pumilus CSURP6343	1.94	130148166
7 (+)	Bacillus pumilus 10403329	1.86	133055080
8 (+)	Bacillus pumilus 10403990	1.78	133055080
9 (+)	Bacillus pumilus 10403607	1.76	133055080

Tableau des résultats pour analyte 4--suite page suivante

Tableau des résultats pour analyte 4 -- suite de la page précédente			
**Classement (Qualité)**	**Profil de référence**	**Score Valeur**	**Identifiant NCBI**
10<br>(+)	Bacillus pumilus CSURP505	1.76	130148166

Analyte 5

**Nom de l'échantillon:**	A5
**Description de l'échantillon:**	
**ID de l'échantillon:**	A5
**Date/Heure de création de l'échantillon:**	2019-07-10T17:04:20.605
**Type de l'échantillon:**	Échantillon standard
**Méthode de classification :**	MALDI Biotyper MSP Identification Standard Method 1.1
**Méthode de prétraitement :**	BioTyper Preprocessing Standard Method 1.2
**Méthode ACQ :**	D:\Methods\flexControlMethods\MBT_FC.par
**Horodatage ACQ :**	2019-07-10T17:06:30.510
**Méthode AutoXecute :**	MBT_AutoX_smart
**Bibliothèque de MSP utilisée:**	Culturomics / f8c211c3-71c5-471b-8a7e-7f6abca59bb9 / 2019-06-28T14:15:05.935, Timone / 29617d84-2a1e-4bf6-a13d-569eecb48f06 / 2018-04-19T13:24:29.884, BDAL / contains 7854 MSPs / e7ef41ca-b750-4d47-9a1c-6c26fa454356 / 2019-02-01T09:48:20.358

Classement (Qualité)	Profil de référence	Score Valeur	Identifiant NCBI
1 (-)	Pichia occidentalis CBS 1910 CBS	1.40	54552
2 (-)	Lactobacillus fructivorans DSM 20203T DSM	1.39	1614
3 (-)	Pseudomonas aeruginosa 19955_1 CHB	1.36	287
4 (-)	Lactobacillus paralimentarius DSM 13238T DSM	1.35	83526
5 (-)	Lactobacillus paracasei ssp paracasei DSM 20207 DSM	1.34	47714
6 (-)	Pseudomonas abietaniphila CIP 106708T HAM	1.32	89065
7 (-)	Streptomyces avidinii B190 UFL	1.32	1895
8 (-)	Slackia exigua 8269163-2	1.31	133993714
9 (-)	Neisseria meningitidis 639 PGM	1.31	487

Tableau des résultats pour analyte 5--suite page suivante

Tableau des résultats pour analyte 5 -- suite de la page précédente			
**Classement (Qualité)**	**Profil de référence**	**Score Valeur**	**Identifiant NCBI**
10<br>(-)	Lactobacillus plantarum DSM 20205 DSM	1.30	1590

Analyte 6

**Nom de l'échantillon:**	A6
**Description de l'échantillon:**	
**ID de l'échantillon:**	A6
**Date/Heure de création de l'échantillon:**	2019-07-10T17:04:20.605
**Type de l'échantillon:**	Échantillon standard
**Méthode de classification :**	MALDI Biotyper MSP Identification Standard Method 1.1
**Méthode de prétraitement :**	BioTyper Preprocessing Standard Method 1.2
**Méthode ACQ :**	D:\Methods\flexControlMethods\MBT_FC.par
**Horodatage ACQ :**	2019-07-10T17:06:50.448
**Méthode AutoXecute :**	MBT_AutoX_smart
**Bibliothèque de MSP utilisée:**	Culturomics / f8c211c3-71c5-471b-8a7e-7f6abca59bb9 / 2019-06-28T14:15:05.935, Timone / 29617d84-2a1e-4bf6-a13d-569eecb48f06 / 2018-04-19T13:24:29.884, BDAL / contains 7854 MSPs / e7ef41ca-b750-4d47-9a1c-6c26fa454356 / 2019-02-01T09:48:20.358

Classement (Qualité)	Profil de référence	Score Valeur	Identifiant NCBI
1 (-)	Lactobacillus paracasei ssp paracasei DSM 2649 DSM	1.41	47714
2 (-)	Lactobacillus paracasei ssp paracasei DSM 5622T DSM	1.38	47714
3 (-)	Clostridium innocuum 1079_ATCC 14501T BOG	1.30	1522
4 (-)	Lactobacillus sakei DSM 6333 DSM	1.29	1599
5 (-)	Staphylococcus lutrae DSM 10244T DSM	1.29	155085
6 (-)	Lactobacillus saerimneri DSM 16049T DSM	1.27	228229
7 (-)	Lactobacillus paracasei ssp tolerans DSM 20258T DSM	1.27	113557
8 (-)	Lactobacillus satsumensis DSM 16230T DSM	1.27	259059
9 (-)	Arthrobacter sp P1 B386 UFL	1.26	1663

Tableau des résultats pour analyte 6--suite page suivante

Tableau des résultats pour analyte 6 -- suite de la page précédente			
**Classement (Qualité)**	**Profil de référence**	**Score Valeur**	**Identifiant NCBI**
10<br>(-)	Lactobacillus pentosus DSM 20199 DSM	1.25	1589

Analyte 7

**Nom de l'échantillon:**	A7
**Description de l'échantillon:**	
**ID de l'échantillon:**	A7
**Date/Heure de création de l'échantillon:**	2019-07-10T17:04:20.606
**Type de l'échantillon:**	Échantillon standard
**Méthode de classification :**	MALDI Biotyper MSP Identification Standard Method 1.1
**Méthode de prétraitement :**	BioTyper Preprocessing Standard Method 1.2
**Méthode ACQ :**	D:\Methods\flexControlMethods\MBT_FC.par
**Horodatage ACQ :**	2019-07-10T17:07:10.949
**Méthode AutoXecute :**	MBT_AutoX_smart
**Bibliothèque de MSP utilisée:**	Culturomics / f8c211c3-71c5-471b-8a7e-7f6abca59bb9 / 2019-06-28T14:15:05.935, Timone / 29617d84-2a1e-4bf6-a13d-569eecb48f06 / 2018-04-19T13:24:29.884, BDAL / contains 7854 MSPs / e7ef41ca-b750-4d47-9a1c-6c26fa454356 / 2019-02-01T09:48:20.358

Classement (Qualité)	Profil de référence	Score Valeur	Identifiant NCBI
1 (+++)	Bacillus pumilus CSURP4226	2.27	130148166
2 (+++)	Bacillus pumilus CSURP8100	2.20	130148166
3 (+++)	Bacillus pumilus CSURP4085	2.17	130148166
4 (+++)	Bacillus pumilus CSURP4105	2.13	130148166
5 (+++)	Bacillus pumilus 10403607	2.03	133055080
6 (+)	Bacillus pumilus 10403329	1.95	133055080
7 (+)	Bacillus pumilus 10403985	1.91	133055080
8 (+)	Bacillus pumilus CSURP6343	1.86	130148166
9 (+)	Bacillus pumilus CSURP6343	1.86	130148166

Tableau des résultats pour analyte 7--suite page suivante

Tableau des résultats pour analyte 7 -- suite de la page précédente			
**Classement (Qualité)**	**Profil de référence**	**Score Valeur**	**Identifiant NCBI**
10 (+)	Bacillus pumilus CSURP8210	1.85	130148166

Analyte 8

**Nom de l'échantillon:**	A8
**Description de l'échantillon:**	
**ID de l'échantillon:**	A8
**Date/Heure de création de l'échantillon:**	2019-07-10T17:04:20.607
**Type de l'échantillon:**	Échantillon standard
**Méthode de classification :**	MALDI Biotyper MSP Identification Standard Method 1.1
**Méthode de prétraitement :**	BioTyper Preprocessing Standard Method 1.2
**Méthode ACQ :**	D:\Methods\flexControlMethods\MBT_FC.par
**Horodatage ACQ :**	2019-07-10T17:07:32.233
**Méthode AutoXecute :**	MBT_AutoX_smart
**Bibliothèque de MSP utilisée:**	Culturomics / f8c211c3-71c5-471b-8a7e-7f6abca59bb9 / 2019-06-28T14:15:05.935, Timone / 29617d84-2a1e-4bf6-a13d-569eecb48f06 / 2018-04-19T13:24:29.884, BDAL / contains 7854 MSPs / e7ef41ca-b750-4d47-9a1c-6c26fa454356 / 2019-02-01T09:48:20.358

Classement (Qualité)	Profil de référence	Score Valeur	Identifiant NCBI
1 (+++)	Bacillus pumilus CSURP4226	2.19	130148166
2 (+++)	Bacillus pumilus CSURP8100	2.16	130148166
3 (+++)	Bacillus pumilus CSURP4085	2.09	130148166
4 (+)	Bacillus pumilus CSURP6343	1.99	130148166
5 (+)	Bacillus pumilus CSURP6343	1.99	130148166
6 (+)	Bacillus pumilus 10403329	1.93	133055080
7 (+)	Bacillus pumilus CSURP4105	1.88	130148166
8 (+)	Bacillus pumilus 10403985	1.85	133055080
9 (+)	Bacillus pumilus 10403607	1.81	133055080

Tableau des résultats pour analyte 8--suite page suivante

Tableau des résultats pour analyte 8 -- suite de la page précédente			
**Classement (Qualité)**	**Profil de référence**	**Score Valeur**	**Identifiant NCBI**
10<br>(+)	Bacillus pumilus 10403987	1.72	133055080

Analyte 9

**Nom de l'échantillon:**	A9
**Description de l'échantillon:**	
**ID de l'échantillon:**	A9
**Date/Heure de création de l'échantillon:**	2019-07-10T17:04:20.608
**Type de l'échantillon:**	Échantillon standard
**Méthode de classification :**	MALDI Biotyper MSP Identification Standard Method 1.1
**Méthode de prétraitement :**	BioTyper Preprocessing Standard Method 1.2
**Méthode ACQ :**	D:\Methods\flexControlMethods\MBT_FC.par
**Horodatage ACQ :**	2019-07-10T17:07:53.207
**Méthode AutoXecute :**	MBT_AutoX_smart
**Bibliothèque de MSP utilisée:**	Culturomics / f8c211c3-71c5-471b-8a7e-7f6abca59bb9 / 2019-06-28T14:15:05.935, Timone / 29617d84-2a1e-4bf6-a13d-569eecb48f06 / 2018-04-19T13:24:29.884, BDAL / contains 7854 MSPs / e7ef41ca-b750-4d47-9a1c-6c26fa454356 / 2019-02-01T09:48:20.358

Classement (Qualité)	Profil de référence	Score Valeur	Identifiant NCBI
1 (+++)	Bacillus pumilus CSURP4226	2.07	130148166
2 (+)	Bacillus pumilus CSURP8100	1.99	130148166
3 (+)	Bacillus pumilus CSURP4085	1.96	130148166
4 (+)	Bacillus pumilus 10403987	1.91	133055080
5 (+)	Bacillus pumilus 10403985	1.81	133055080
6 (+)	Bacillus pumilus 10403329	1.80	133055080
7 (+)	Bacillus pumilus 10403607	1.80	133055080
8 (+)	Bacillus pumilus CSURP4105	1.78	130148166
9 (+)	Bacillus pumilus 10403751	1.75	133055080

Tableau des résultats pour analyte 9--suite page suivante

Tableau des résultats pour analyte 9 -- suite de la page précédente

Classement (Qualité)	Profil de référence	Score Valeur	Identifiant NCBI
10 (+)	Bacillus pumilus 10403990	1.74	133055080

Analyte 10

**Nom de l'échantillon:**	A10
**Description de l'échantillon:**	
**ID de l'échantillon:**	A10
**Date/Heure de création de l'échantillon:**	2019-07-10T17:04:20.609
**Type de l'échantillon:**	Échantillon standard
**Méthode de classification :**	MALDI Biotyper MSP Identification Standard Method 1.1
**Méthode de prétraitement :**	BioTyper Preprocessing Standard Method 1.2
**Méthode ACQ :**	D:\Methods\flexControlMethods\MBT_FC.par
**Horodatage ACQ :**	2019-07-10T17:08:14.823
**Méthode AutoXecute :**	MBT_AutoX_smart
**Bibliothèque de MSP utilisée:**	Culturomics / f8c211c3-71c5-471b-8a7e-7f6abca59bb9 / 2019-06-28T14:15:05.935, Timone / 29617d84-2a1e-4bf6-a13d-569eecb48f06 / 2018-04-19T13:24:29.884, BDAL / contains 7854 MSPs / e7ef41ca-b750-4d47-9a1c-6c26fa454356 / 2019-02-01T09:48:20.358

Classement (Qualité)	Profil de référence	Score Valeur	Identifiant NCBI
1 (+++)	Bacillus pumilus CSURP4226	2.30	130148166
2 (+++)	Bacillus pumilus CSURP8100	2.18	130148166
3 (+++)	Bacillus pumilus CSURP4085	2.17	130148166
4 (+++)	Bacillus pumilus CSURP4105	2.11	130148166
5 (+)	Bacillus pumilus CSURP6343	1.94	130148166
6 (+)	Bacillus pumilus CSURP6343	1.94	130148166
7 (+)	Bacillus pumilus 10403985	1.88	133055080
8 (+)	Bacillus pumilus CSURP505	1.88	130148166
9 (+)	Bacillus pumilus 10403329	1.87	133055080

Tableau des résultats pour analyte 10--suite page suivante

Tableau des résultats pour analyte 10 -- suite de la page précédente			
**Classement (Qualité)**	**Profil de référence**	**Score Valeur**	**Identifiant NCBI**
10<br>(+)	Bacillus pumilus 10403987	1.86	133055080

Analyte 11

Nom de l'échantillon: A11
Description de l'échantillon:
ID de l'échantillon: A11
Date/Heure de création de l'échantillon: 2019-07-10T17:04:20.610
Type de l'échantillon: Échantillon standard
Méthode de classification : MALDI Biotyper MSP Identification Standard Method 1.1
Méthode de prétraitement : BioTyper Preprocessing Standard Method 1.2
Méthode ACQ : D:\Methods\flexControlMethods\MBT_FC.par
Horodatage ACQ : 2019-07-10T17:08:36.892
Méthode AutoXecute : MBT_AutoX_smart
Bibliothèque de MSP utilisée: Culturomics / f8c211c3-71c5-471b-8a7e-7f6abca59bb9 / 2019-06-28T14:15:05.935, Timone / 29617d84-2a1e-4bf6-a13d-569eecb48f06 / 2018-04-19T13:24:29.884, BDAL / contains 7854 MSPs / e7ef41ca-b750-4d47-9a1c-6c26fa454356 / 2019-02-01T09:48:20.358

Classement (Qualité)	Profil de référence	Score Valeur	Identifiant NCBI
1 (+++)	Bacillus pumilus CSURP4085	2.06	130148166
2 (+)	Bacillus pumilus CSURP4226	1.97	130148166
3 (+)	Bacillus pumilus CSURP4105	1.96	130148166
4 (+)	Bacillus pumilus CSURP8100	1.95	130148166
5 (+)	Bacillus pumilus 10403985	1.92	133055080
6 (+)	Bacillus pumilus 10403607	1.90	133055080
7 (+)	Bacillus pumilus 10403987	1.84	133055080
8 (+)	Bacillus pumilus 10403329	1.84	133055080
9 (+)	Bacillus pumilus CSURP6343	1.80	130148166

Tableau des résultats pour analyte 11--suite page suivante

Tableau des résultats pour analyte 11 -- suite de la page précédente			
**Classement (Qualité)**	**Profil de référence**	**Score Valeur**	**Identifiant NCBI**
10<br>(+)	Bacillus pumilus CSURP6343	1.80	130148166

Analyte 12

**Nom de l'échantillon:**	A12
**Description de l'échantillon:**	
**ID de l'échantillon:**	A12
**Date/Heure de création de l'échantillon:**	2019-07-10T17:04:20.611
**Type de l'échantillon:**	Échantillon standard
**Méthode de classification :**	MALDI Biotyper MSP Identification Standard Method 1.1
**Méthode de prétraitement :**	BioTyper Preprocessing Standard Method 1.2
**Méthode ACQ :**	D:\Methods\flexControlMethods\MBT_FC.par
**Horodatage ACQ :**	2019-07-10T17:08:58.385
**Méthode AutoXecute :**	MBT_AutoX_smart
**Bibliothèque de MSP utilisée:**	Culturomics / f8c211c3-71c5-471b-8a7e-7f6abca59bb9 / 2019-06-28T14:15:05.935, Timone / 29617d84-2a1e-4bf6-a13d-569eecb48f06 / 2018-04-19T13:24:29.884, BDAL / contains 7854 MSPs / e7ef41ca-b750-4d47-9a1c-6c26fa454356 / 2019-02-01T09:48:20.358

Classement (Qualité)	Profil de référence	Score Valeur	Identifiant NCBI
1 (+++)	Bacillus pumilus CSURP4226	2.09	130148166
2 (+)	Bacillus pumilus CSURP4085	1.98	130148166
3 (+)	Bacillus pumilus 10403987	1.87	133055080
4 (+)	Bacillus pumilus 10403990	1.84	133055080
5 (+)	Bacillus pumilus CSURP8100	1.82	130148166
6 (+)	Bacillus pumilus 10403607	1.78	133055080
7 (+)	Bacillus pumilus CSURP4105	1.77	130148166
8 (+)	Bacillus pumilus 10403751	1.77	133055080
9 (+)	Bacillus pumilus 10403985	1.75	133055080

Tableau des résultats pour analyte 12--suite page suivante

Tableau des résultats pour analyte 12 -- suite de la page précédente			
**Classement (Qualité)**	**Profil de référence**	**Score Valeur**	**Identifiant NCBI**
10<br>(+)	Bacillus pumilus 10403329	1.75	133055080

Analyte 13

**Nom de l'échantillon:**	B1
**Description de l'échantillon:**	
**ID de l'échantillon:**	B1
**Date/Heure de création de l'échantillon:**	2019-07-10T17:04:20.612
**Type de l'échantillon:**	Échantillon standard
**Méthode de classification :**	MALDI Biotyper MSP Identification Standard Method 1.1
**Méthode de prétraitement :**	BioTyper Preprocessing Standard Method 1.2
**Méthode ACQ :**	D:\Methods\flexControlMethods\MBT_FC.par
**Horodatage ACQ :**	2019-07-10T17:09:19.634
**Méthode AutoXecute :**	MBT_AutoX_smart
**Bibliothèque de MSP utilisée:**	Culturomics / f8c211c3-71c5-471b-8a7e-7f6abca59bb9 / 2019-06-28T14:15:05.935, Timone / 29617d84-2a1e-4bf6-a13d-569eecb48f06 / 2018-04-19T13:24:29.884, BDAL / contains 7854 MSPs / e7ef41ca-b750-4d47-9a1c-6c26fa454356 / 2019-02-01T09:48:20.358

Classement (Qualité)	Profil de référence	Score Valeur	Identifiant NCBI
1 (+)	Turicella otitidis CSURP4453	1.71	130148166
2 (-)	Magnusomyces capitatus CSURP3898	1.64	130148166
3 (-)	Turicella otitidis CSURP6057	1.64	130148166
4 (-)	Corynebacterium accolens CSURP7679	1.64	130148166
5 (-)	Kocuria rhizophila CSURP9116	1.63	130148166
6 (-)	Lactobacillus johnsonii CSURP4452	1.57	130148166
7 (-)	Dolosigranulum pigrum CSURP6052	1.41	130148166
8 (-)	Weissella cibaria CSURP4100	1.28	130148166
9 (-)	Kocuria rhizophila CSURP7443	1.27	130148166

Tableau des résultats pour analyte 13--suite page suivante

Tableau des résultats pour analyte 13 -- suite de la page précédente			
**Classement (Qualité)**	**Profil de référence**	**Score Valeur**	**Identifiant NCBI**
10<br>(-)	Kocuria rhizophila CSURP8518	1.26	130148166

Analyte 14

**Nom de l'échantillon:**	B2
**Description de l'échantillon:**	
**ID de l'échantillon:**	B2
**Date/Heure de création de l'échantillon:**	2019-07-10T17:04:20.613
**Type de l'échantillon:**	Échantillon standard
**Méthode de classification :**	MALDI Biotyper MSP Identification Standard Method 1.1
**Méthode de prétraitement :**	BioTyper Preprocessing Standard Method 1.2
**Méthode ACQ :**	D:\Methods\flexControlMethods\MBT_FC.par
**Horodatage ACQ :**	2019-07-10T17:09:40.611
**Méthode AutoXecute :**	MBT_AutoX_smart
**Bibliothèque de MSP utilisée:**	Culturomics / f8c211c3-71c5-471b-8a7e-7f6abca59bb9 / 2019-06-28T14:15:05.935, Timone / 29617d84-2a1e-4bf6-a13d-569eecb48f06 / 2018-04-19T13:24:29.884, BDAL / contains 7854 MSPs / e7ef41ca-b750-4d47-9a1c-6c26fa454356 / 2019-02-01T09:48:20.358

Classement (Qualité)	Profil de référence	Score Valeur	Identifiant NCBI
1 (+)	Kocuria rhizophila CSURP9116	1.71	130148166
2 (-)	Corynebacterium accolens CSURP7679	1.68	130148166
3 (-)	Kocuria rhizophila CSURP7443	1.65	130148166
4 (-)	Turicella otitidis CSURP6057	1.64	130148166
5 (-)	Magnusomyces capitatus CSURP3898	1.62	130148166
6 (-)	Dolosigranulum pigrum CSURP6052	1.52	130148166
7 (-)	Kocuria rhizophila DSM 348 DSM	1.31	72000
8 (-)	Kocuria rhizophila DSM 46222 DSM	1.30	72000
9 (-)	Pseudarthrobacter polychromogenes IMET 11071T HKJ	1.30	1676

Tableau des résultats pour analyte 14--suite page suivante

Tableau des résultats pour analyte 14 -- suite de la page précédente			
**Classement (Qualité)**	**Profil de référence**	**Score Valeur**	**Identifiant NCBI**
10<br>(-)	Turicella otitidis CSURP4453	1.29	130148166

Analyte 15

**Nom de l'échantillon:**	B3
**Description de l'échantillon:**	
**ID de l'échantillon:**	B3
**Date/Heure de création de l'échantillon:**	2019-07-10T17:04:20.614
**Type de l'échantillon:**	Échantillon standard
**Méthode de classification :**	MALDI Biotyper MSP Identification Standard Method 1.1
**Méthode de prétraitement :**	BioTyper Preprocessing Standard Method 1.2
**Méthode ACQ :**	D:\Methods\flexControlMethods\MBT_FC.par
**Horodatage ACQ :**	2019-07-10T17:10:00.863
**Méthode AutoXecute :**	MBT_AutoX_smart
**Bibliothèque de MSP utilisée:**	Culturomics / f8c211c3-71c5-471b-8a7e-7f6abca59bb9 / 2019-06-28T14:15:05.935, Timone / 29617d84-2a1e-4bf6-a13d-569eecb48f06 / 2018-04-19T13:24:29.884, BDAL / contains 7854 MSPs / e7ef41ca-b750-4d47-9a1c-6c26fa454356 / 2019-02-01T09:48:20.358

Classement (Qualité)	Profil de référence	Score Valeur	Identifiant NCBI
1 (-)	Phoenicibacter massiliensis P5887P	1.49	130148166
2 (-)	180618-IF2-Thao24 P7771P	1.16	130136093
3 (-)	Corynebacterium tuberculostearicum CSURP4602	1.15	130148166
4 (-)	Corynebacterium tuberculostearicum CSUP4602	1.15	130148166
5 (-)	Streptococcus anginosus CIP 102921T	1.09	133055080
6 (-)	Clostridium histolyticum 1036_NCTC 503T BOG	1.08	1498
7 (-)	Micrococcus luteus CSURP792	1.08	130148166
8 (-)	Bacillus massiligabonensis P6262P	1.07	130148166
9 (-)	Bacillus soli CSURP3680	1.06	130148166

Tableau des résultats pour analyte 15--suite page suivante

Tableau des résultats pour analyte 15 -- suite de la page précédente			
**Classement (Qualité)**	**Profil de référence**	**Score Valeur**	**Identifiant NCBI**
10<br>(-)	Corynebacterium coyleae DSM 44184T DSM	1.05	53374

Analyte 16

**Nom de l'échantillon:**	B4
**Description de l'échantillon:**	
**ID de l'échantillon:**	B4
**Date/Heure de création de l'échantillon:**	2019-07-10T17:04:20.615
**Type de l'échantillon:**	Échantillon standard
**Méthode de classification :**	MALDI Biotyper MSP Identification Standard Method 1.1
**Méthode de prétraitement :**	BioTyper Preprocessing Standard Method 1.2
**Méthode ACQ :**	D:\Methods\flexControlMethods\MBT_FC.par
**Horodatage ACQ :**	2019-07-10T17:10:17.735
**Méthode AutoXecute :**	MBT_AutoX_smart
**Bibliothèque de MSP utilisée:**	Culturomics / f8c211c3-71c5-471b-8a7e-7f6abca59bb9 / 2019-06-28T14:15:05.935, Timone / 29617d84-2a1e-4bf6-a13d-569eecb48f06 / 2018-04-19T13:24:29.884, BDAL / contains 7854 MSPs / e7ef41ca-b750-4d47-9a1c-6c26fa454356 / 2019-02-01T09:48:20.358

Classement (Qualité)	Profil de référence	Score Valeur	Identifiant NCBI
1 (-)	Urmitella massiliensis CSURP3072	1.38	130148166
2 (-)	Streptococcus equi_ssp_zooepidemicus ATCC 43079T THL	1.28	40041
3 (-)	Streptococcus equi_ssp_ruminatorum DSM 17037T DSM	1.25	254358
4 (-)	Marinibacillus marinus DSM 1297T DSM	1.24	86667
5 (-)	Streptococcus dysgalactiae CSURP7579	1.24	130148166
6 (-)	Burkholderia tuberum LMG 21444T HAM	1.23	157910
7 (-)	Acidaminococcus fermentans ENR_0552 ENR	1.23	905
8 (-)	Streptococcus pyogenes CSURP5499	1.22	130148166
9 (-)	Lactobacillus sakei DSM 20494 DSM	1.20	1599

Tableau des résultats pour analyte 16--suite page suivante

Tableau des résultats pour analyte 16 -- suite de la page précédente			
**Classement (Qualité)**	**Profil de référence**	**Score Valeur**	**Identifiant NCBI**
10<br>(-)	181204-IF5-Thao34-CSURP9190	1.20	130136093

Analyte 17

**Nom de l’échantillon:**	B5
**Description de l’échantillon:**	
**ID de l’échantillon:**	B5
**Date/Heure de création de l’échantillon:**	2019-07-10T17:04:20.616
**Type de l’échantillon:**	Échantillon standard
**Méthode de classification :**	MALDI Biotyper MSP Identification Standard Method 1.1
**Méthode de prétraitement :**	BioTyper Preprocessing Standard Method 1.2
**Méthode ACQ :**	D:\Methods\flexControlMethods\MBT_FC.par
**Horodatage ACQ :**	2019-07-10T17:10:39.215
**Méthode AutoXecute :**	MBT_AutoX_smart
**Bibliothèque de MSP utilisée:**	Culturomics / f8c211c3-71c5-471b-8a7e-7f6abca59bb9 / 2019-06-28T14:15:05.935, Timone / 29617d84-2a1e-4bf6-a13d-569eecb48f06 / 2018-04-19T13:24:29.884, BDAL / contains 7854 MSPs / e7ef41ca-b750-4d47-9a1c-6c26fa454356 / 2019-02-01T09:48:20.358

Classement (Qualité)	Profil de référence	Score Valeur	Identifiant NCBI
1 (+++)	Bacillus pumilus CSURP4226	2.44	130148166
2 (+++)	Bacillus pumilus CSURP8100	2.29	130148166
3 (+++)	Bacillus pumilus CSURP4085	2.16	130148166
4 (+++)	Bacillus pumilus CSURP6343	2.09	130148166
5 (+++)	Bacillus pumilus CSURP6343	2.09	130148166
6 (+++)	Bacillus pumilus CSURP4105	2.04	130148166
7 (+)	Bacillus pumilus CSURP8210	1.94	130148166
8 (+)	Bacillus pumilus 10403607	1.92	133055080
9 (+)	Bacillus pumilus CSURP3862	1.89	130148166

Tableau des résultats pour analyte 17--suite page suivante

Tableau des résultats pour analyte 17 -- suite de la page précédente			
**Classement (Qualité)**	**Profil de référence**	**Score Valeur**	**Identifiant NCBI**
10 (+)	Bacillus pumilus 10403987	1.86	133055080

Analyte 18

**Nom de l'échantillon:**	B6
**Description de l'échantillon:**	
**ID de l'échantillon:**	B6
**Date/Heure de création de l'échantillon:**	2019-07-10T17:04:20.617
**Type de l'échantillon:**	Échantillon standard
**Méthode de classification :**	MALDI Biotyper MSP Identification Standard Method 1.1
**Méthode de prétraitement :**	BioTyper Preprocessing Standard Method 1.2
**Méthode ACQ :**	D:\Methods\flexControlMethods\MBT_FC.par
**Horodatage ACQ :**	2019-07-10T17:11:00.166
**Méthode AutoXecute :**	MBT_AutoX_smart
**Bibliothèque de MSP utilisée:**	Culturomics / f8c211c3-71c5-471b-8a7e-7f6abca59bb9 / 2019-06-28T14:15:05.935, Timone / 29617d84-2a1e-4bf6-a13d-569eecb48f06 / 2018-04-19T13:24:29.884, BDAL / contains 7854 MSPs / e7ef41ca-b750-4d47-9a1c-6c26fa454356 / 2019-02-01T09:48:20.358

Classement (Qualité)	Profil de référence	Score Valeur	Identifiant NCBI
1 (-)	Bacillus pumilus 10403987	1.52	133055080
2 (-)	Bacillus pumilus 10403329	1.49	133055080
3 (-)	Bacillus pumilus 10403990	1.43	133055080
4 (-)	Bacillus pumilus CSURP6343	1.41	130148166
5 (-)	Bacillus pumilus CSURP6343	1.41	130148166
6 (-)	Bacillus pumilus 10403985	1.38	133055080
7 (-)	Lactobacillus zeae DSM 20178T DSM	1.33	57037
8 (-)	Bacillus pumilus 10403751	1.32	133055080
9 (-)	Bacillus pumilus CSURP4105	1.31	130148166

Tableau des résultats pour analyte 18--suite page suivante

Tableau des résultats pour analyte 18 -- suite de la page précédente			
**Classement (Qualité)**	**Profil de référence**	**Score Valeur**	**Identifiant NCBI**
10<br>(-)	Bacillus pumilus CSURP4226	1.29	130148166

Analyte 19

**Nom de l'échantillon:**	B7
**Description de l'échantillon:**	
**ID de l'échantillon:**	B7
**Date/Heure de création de l'échantillon:**	2019-07-10T17:04:20.618
**Type de l'échantillon:**	Échantillon standard
**Méthode de classification :**	MALDI Biotyper MSP Identification Standard Method 1.1
**Méthode de prétraitement :**	BioTyper Preprocessing Standard Method 1.2
**Méthode ACQ :**	D:\Methods\flexControlMethods\MBT_FC.par
**Horodatage ACQ :**	2019-07-10T17:11:20.303
**Méthode AutoXecute :**	MBT_AutoX_smart
**Bibliothèque de MSP utilisée:**	Culturomics / f8c211c3-71c5-471b-8a7e-7f6abca59bb9 / 2019-06-28T14:15:05.935, Timone / 29617d84-2a1e-4bf6-a13d-569eecb48f06 / 2018-04-19T13:24:29.884, BDAL / contains 7854 MSPs / e7ef41ca-b750-4d47-9a1c-6c26fa454356 / 2019-02-01T09:48:20.358

Classement (Qualité)	Profil de référence	Score Valeur	Identifiant NCBI
1 (+++)	Lysinibacillus fusiformis CSURP4522	2.16	130148166
2 (+++)	Lysinibacillus fusiformis CSURP2236	2.13	130148166
3 (+++)	Lysinibacillus boronitolerans DSM 17140T DSM	2.08	309788
4 (+)	Lysinibacillus fusiformis DSM 493 DSM	1.97	28031
5 (+)	Lysinibacillus fusiformis DSM 2898T BRB	1.95	28031
6 (+)	Lysinibacillus fusiformis DSM 2898T DSM	1.87	28031
7 (+)	Lysinibacillus sphaericus DSM 2899 DSM	1.81	1421
8 (+)	Lysinibacillus fusiformis CSURP5657	1.81	130148166
9 (+)	Lysinibacillus fusiformis CSURP8109	1.79	130148166

Tableau des résultats pour analyte 19--suite page suivante

Tableau des résultats pour analyte 19 -- suite de la page précédente			
**Classement (Qualité)**	**Profil de référence**	**Score Valeur**	**Identifiant NCBI**
10<br>(+)	Lysinibacillus fusiformis CSURP1153	1.78	130148166

Analyte 20

**Nom de l'échantillon:**	B8
**Description de l'échantillon:**	
**ID de l'échantillon:**	B8
**Date/Heure de création de l'échantillon:**	2019-07-10T17:04:20.620
**Type de l'échantillon:**	Échantillon standard
**Méthode de classification :**	MALDI Biotyper MSP Identification Standard Method 1.1
**Méthode de prétraitement :**	BioTyper Preprocessing Standard Method 1.2
**Méthode ACQ :**	D:\Methods\flexControlMethods\MBT_FC.par
**Horodatage ACQ :**	2019-07-10T17:11:41.468
**Méthode AutoXecute :**	MBT_AutoX_smart
**Bibliothèque de MSP utilisée:**	Culturomics / f8c211c3-71c5-471b-8a7e-7f6abca59bb9 / 2019-06-28T14:15:05.935, Timone / 29617d84-2a1e-4bf6-a13d-569eecb48f06 / 2018-04-19T13:24:29.884, BDAL / contains 7854 MSPs / e7ef41ca-b750-4d47-9a1c-6c26fa454356 / 2019-02-01T09:48:20.358

Classement (Qualité)	Profil de référence	Score Valeur	Identifiant NCBI
1 (+++)	Lysinibacillus fusiformis CSURP4522	2.13	130148166
2 (+++)	Lysinibacillus fusiformis CSURP5657	2.08	130148166
3 (+++)	Lysinibacillus fusiformis CSURP2236	2.06	130148166
4 (+)	Lysinibacillus boronitolerans DSM 17140T DSM	1.99	309788
5 (+)	Lysinibacillus xylanilyticus CICC 20858 CICC	1.94	400634
6 (+)	Lysinibacillus fusiformis CSURP1153	1.91	130148166
7 (+)	Lysinibacillus fusiformis DSM 2898T DSM	1.88	28031
8 (+)	Lysinibacillus fusiformis DSM 2898T BRB	1.85	28031
9 (+)	Lysinibacillus fusiformis CSURP8109	1.82	130148166

Tableau des résultats pour analyte 20--suite page suivante

Tableau des résultats pour analyte 20 -- suite de la page précédente			
**Classement (Qualité)**	**Profil de référence**	**Score Valeur**	**Identifiant NCBI**
10 (+)	Lysinibacillus fusiformis DSM 493 DSM	1.75	28031

Analyte 21

Nom de l'échantillon: B9
Description de l'échantillon:
ID de l'échantillon: B9
Date/Heure de création de l'échantillon: 2019-07-10T17:04:20.621
Type de l'échantillon: Échantillon standard
Méthode de classification : MALDI Biotyper MSP Identification Standard Method 1.1
Méthode de prétraitement : BioTyper Preprocessing Standard Method 1.2
Méthode ACQ : D:\Methods\flexControlMethods\MBT_FC.par
Horodatage ACQ : 2019-07-10T17:12:02.960
Méthode AutoXecute : MBT_AutoX_smart
Bibliothèque de MSP utilisée: Culturomics / f8c211c3-71c5-471b-8a7e-7f6abca59bb9 / 2019-06-28T14:15:05.935, Timone / 29617d84-2a1e-4bf6-a13d-569eecb48f06 / 2018-04-19T13:24:29.884, BDAL / contains 7854 MSPs / e7ef41ca-b750-4d47-9a1c-6c26fa454356 / 2019-02-01T09:48:20.358

Classement (Qualité)	Profil de référence	Score Valeur	Identifiant NCBI
1 (+++)	Lysinibacillus fusiformis CSURP4522	2.13	130148166
2 (+++)	Lysinibacillus fusiformis CSURP2236	2.13	130148166
3 (+)	Lysinibacillus boronitolerans DSM 17140T DSM	1.99	309788
4 (+)	Lysinibacillus fusiformis CSURP5657	1.99	130148166
5 (+)	Lysinibacillus fusiformis DSM 2898T DSM	1.94	28031
6 (+)	Lysinibacillus fusiformis DSM 493 DSM	1.83	28031
7 (+)	Lysinibacillus fusiformis CSURP8109	1.82	130148166
8 (+)	Lysinibacillus fusiformis DSM 2898T BRB	1.81	28031
9 (+)	Lysinibacillus fusiformis CSURP1153	1.81	130148166

Tableau des résultats pour analyte 21--suite page suivante

Tableau des résultats pour analyte 21 -- suite de la page précédente			
**Classement (Qualité)**	**Profil de référence**	**Score Valeur**	**Identifiant NCBI**
10 (+)	Lysinibacillus sphaericus DSM 2899 DSM	1.79	1421

Analyte 22

**Nom de l'échantillon:**	B10
**Description de l'échantillon:**	
**ID de l'échantillon:**	B10
**Date/Heure de création de l'échantillon:**	2019-07-10T17:04:20.622
**Type de l'échantillon:**	Échantillon standard
**Méthode de classification :**	MALDI Biotyper MSP Identification Standard Method 1.1
**Méthode de prétraitement :**	BioTyper Preprocessing Standard Method 1.2
**Méthode ACQ :**	D:\Methods\flexControlMethods\MBT_FC.par
**Horodatage ACQ :**	2019-07-10T17:12:23.836
**Méthode AutoXecute :**	MBT_AutoX_smart
**Bibliothèque de MSP utilisée:**	Culturomics / f8c211c3-71c5-471b-8a7e-7f6abca59bb9 / 2019-06-28T14:15:05.935, Timone / 29617d84-2a1e-4bf6-a13d-569eecb48f06 / 2018-04-19T13:24:29.884, BDAL / contains 7854 MSPs / e7ef41ca-b750-4d47-9a1c-6c26fa454356 / 2019-02-01T09:48:20.358

Classement (Qualité)	Profil de référence	Score Valeur	Identifiant NCBI
1 (+++)	Lysinibacillus fusiformis CSURP5657	2.02	130148166
2 (+++)	Lysinibacillus fusiformis CSURP1153	2.01	130148166
3 (+)	Lysinibacillus fusiformis CSURP2236	1.99	130148166
4 (+)	Lysinibacillus fusiformis DSM 2898T DSM	1.90	28031
5 (+)	Lysinibacillus xylanilyticus CICC 20858 CICC	1.82	400634
6 (+)	Lysinibacillus fusiformis	1.76	133993714
7 (+)	Lysinibacillus fusiformis CSURP8109	1.74	130148166
8 (+)	Lysinibacillus fusiformis CSURP4522	1.74	130148166
9 (-)	Lysinibacillus sphaericus DSM 2899 DSM	1.63	1421

Tableau des résultats pour analyte 22--suite page suivante

Tableau des résultats pour analyte 22 -- suite de la page précédente			
**Classement (Qualité)**	**Profil de référence**	**Score Valeur**	**Identifiant NCBI**
10 (-)	Lysinibacillus fusiformis DSM 2898T BRB	1.61	28031

Analyte 23

**Nom de l'échantillon:**	B11
**Description de l'échantillon:**	
**ID de l'échantillon:**	B11
**Date/Heure de création de l'échantillon:**	2019-07-10T17:04:20.623
**Type de l'échantillon:**	Échantillon standard
**Méthode de classification :**	MALDI Biotyper MSP Identification Standard Method 1.1
**Méthode de prétraitement :**	BioTyper Preprocessing Standard Method 1.2
**Méthode ACQ :**	D:\Methods\flexControlMethods\MBT_FC.par
**Horodatage ACQ :**	2019-07-10T17:12:43.659
**Méthode AutoXecute :**	MBT_AutoX_smart
**Bibliothèque de MSP utilisée:**	Culturomics / f8c211c3-71c5-471b-8a7e-7f6abca59bb9 / 2019-06-28T14:15:05.935, Timone / 29617d84-2a1e-4bf6-a13d-569eecb48f06 / 2018-04-19T13:24:29.884, BDAL / contains 7854 MSPs / e7ef41ca-b750-4d47-9a1c-6c26fa454356 / 2019-02-01T09:48:20.358

Classement (Qualité)	Profil de référence	Score Valeur	Identifiant NCBI
1 (+++)	Bacillus pumilus CSURP8100	2.03	130148166
2 (+)	Bacillus pumilus CSURP4085	1.98	130148166
3 (+)	Bacillus pumilus CSURP4105	1.77	130148166
4 (+)	Bacillus pumilus 10403329	1.76	133055080
5 (+)	Bacillus pumilus 10403206	1.72	133055080
6 (-)	Bacillus pumilus CSURP6343	1.66	130148166
7 (-)	Bacillus pumilus CSURP6343	1.66	130148166
8 (-)	Bacillus pumilus 10403987	1.64	133055080
9 (-)	Bacillus pumilus CSURP4226	1.62	130148166

Tableau des résultats pour analyte 23--suite page suivante

Tableau des résultats pour analyte 23 -- suite de la page précédente			
**Classement (Qualité)**	**Profil de référence**	**Score Valeur**	**Identifiant NCBI**
10<br>(-)	Bacillus pumilus 10403607	1.51	133055080

Analyte 24

**Nom de l'échantillon:**	B12
**Description de l'échantillon:**	
**ID de l'échantillon:**	B12
**Date/Heure de création de l'échantillon:**	2019-07-10T17:04:20.625
**Type de l'échantillon:**	Échantillon standard
**Méthode de classification :**	MALDI Biotyper MSP Identification Standard Method 1.1
**Méthode de prétraitement :**	BioTyper Preprocessing Standard Method 1.2
**Méthode ACQ :**	D:\Methods\flexControlMethods\MBT_FC.par
**Horodatage ACQ :**	2019-07-10T17:13:03.955
**Méthode AutoXecute :**	MBT_AutoX_smart
**Bibliothèque de MSP utilisée:**	Culturomics / f8c211c3-71c5-471b-8a7e-7f6abca59bb9 / 2019-06-28T14:15:05.935, Timone / 29617d84-2a1e-4bf6-a13d-569eecb48f06 / 2018-04-19T13:24:29.884, BDAL / contains 7854 MSPs / e7ef41ca-b750-4d47-9a1c-6c26fa454356 / 2019-02-01T09:48:20.358

Classement (Qualité)	Profil de référence	Score Valeur	Identifiant NCBI
1 (-)	Bacillus pumilus 10403329	1.47	133055080
2 (-)	Bacillus pumilus 10403206	1.39	133055080
3 (-)	Bacillus pumilus 10403987	1.33	133055080
4 (-)	Bacillus pumilus CSURP6343	1.32	130148166
5 (-)	Bacillus pumilus CSURP6343	1.32	130148166
6 (-)	Bacillus pumilus CSURP4085	1.32	130148166
7 (-)	Clostridium novyi 161006283 HLG	1.26	1542
8 (-)	Bacillus pumilus 10403990	1.23	133055080
9 (-)	Bacillus pumilus CSURP4105	1.21	130148166

Tableau des résultats pour analyte 24--suite page suivante

Tableau des résultats pour analyte 24 -- suite de la page précédente			
**Classement (Qualité)**	**Profil de référence**	**Score Valeur**	**Identifiant NCBI**
10<br>(-)	Morganella morganii 9544_1 CHB	1.20	582

Analyte 25

**Nom de l'échantillon:**	C1
**Description de l'échantillon:**	
**ID de l'échantillon:**	C1
**Date/Heure de création de l'échantillon:**	2019-07-10T17:04:20.626
**Type de l'échantillon:**	Échantillon standard
**Méthode de classification :**	MALDI Biotyper MSP Identification Standard Method 1.1
**Méthode de prétraitement :**	BioTyper Preprocessing Standard Method 1.2
**Méthode ACQ :**	D:\Methods\flexControlMethods\MBT_FC.par
**Horodatage ACQ :**	2019-07-10T17:13:24.902
**Méthode AutoXecute :**	MBT_AutoX_smart
**Bibliothèque de MSP utilisée:**	Culturomics / f8c211c3-71c5-471b-8a7e-7f6abca59bb9 / 2019-06-28T14:15:05.935, Timone / 29617d84-2a1e-4bf6-a13d-569eecb48f06 / 2018-04-19T13:24:29.884, BDAL / contains 7854 MSPs / e7ef41ca-b750-4d47-9a1c-6c26fa454356 / 2019-02-01T09:48:20.358

Classement (Qualité)	Profil de référence	Score Valeur	Identifiant NCBI
1 (+++)	Bacillus pumilus CSURP4105	2.17	130148166
2 (+++)	Bacillus pumilus CSURP6343	2.13	130148166
3 (+++)	Bacillus pumilus CSURP6343	2.13	130148166
4 (+++)	Bacillus pumilus CSURP8100	2.10	130148166
5 (+++)	Bacillus pumilus CSURP4085	2.03	130148166
6 (+)	Bacillus pumilus 10149151	1.92	133055080
7 (+)	Bacillus pumilus 10403607	1.92	133055080
8 (+)	Bacillus pumilus CSURP4226	1.89	130148166
9 (+)	Bacillus pumilus 10403751	1.88	133055080

Tableau des résultats pour analyte 25--suite page suivante

Tableau des résultats pour analyte 25 -- suite de la page précédente			
**Classement (Qualité)**	**Profil de référence**	**Score Valeur**	**Identifiant NCBI**
10<br>(+)	Bacillus pumilus CSURP3862	1.86	130148166

Analyte 26

**Nom de l'échantillon:**	C2
**Description de l'échantillon:**	
**ID de l'échantillon:**	C2
**Date/Heure de création de l'échantillon:**	2019-07-10T17:04:20.627
**Type de l'échantillon:**	Échantillon standard
**Méthode de classification :**	MALDI Biotyper MSP Identification Standard Method 1.1
**Méthode de prétraitement :**	BioTyper Preprocessing Standard Method 1.2
**Méthode ACQ :**	D:\Methods\flexControlMethods\MBT_FC.par
**Horodatage ACQ :**	2019-07-10T17:13:46.024
**Méthode AutoXecute :**	MBT_AutoX_smart
**Bibliothèque de MSP utilisée:**	Culturomics / f8c211c3-71c5-471b-8a7e-7f6abca59bb9 / 2019-06-28T14:15:05.935, Timone / 29617d84-2a1e-4bf6-a13d-569eecb48f06 / 2018-04-19T13:24:29.884, BDAL / contains 7854 MSPs / e7ef41ca-b750-4d47-9a1c-6c26fa454356 / 2019-02-01T09:48:20.358

Classement (Qualité)	Profil de référence	Score Valeur	Identifiant NCBI
1 (+++)	Bacillus pumilus CSURP4085	2.09	130148166
2 (+)	Bacillus pumilus CSURP8100	1.99	130148166
3 (+)	Bacillus pumilus CSURP6343	1.86	130148166
4 (+)	Bacillus pumilus CSURP6343	1.86	130148166
5 (+)	Bacillus pumilus CSURP4226	1.83	130148166
6 (+)	Bacillus pumilus 10403329	1.74	133055080
7 (-)	Bacillus pumilus 10403206	1.64	133055080
8 (-)	Bacillus pumilus CSURP4105	1.64	130148166
9 (-)	Bacillus pumilus 10403607	1.52	133055080

Tableau des résultats pour analyte 26--suite page suivante

Tableau des résultats pour analyte 26 -- suite de la page précédente			
**Classement (Qualité)**	**Profil de référence**	**Score Valeur**	**Identifiant NCBI**
10<br>(-)	Bacillus pumilus CSURP8210	1.50	130148166

Analyte 27

**Nom de l'échantillon:**	C3
**Description de l'échantillon:**	
**ID de l'échantillon:**	C3
**Date/Heure de création de l'échantillon:**	2019-07-10T17:04:20.629
**Type de l'échantillon:**	Échantillon standard
**Méthode de classification :**	MALDI Biotyper MSP Identification Standard Method 1.1
**Méthode de prétraitement :**	BioTyper Preprocessing Standard Method 1.2
**Méthode ACQ :**	D:\Methods\flexControlMethods\MBT_FC.par
**Horodatage ACQ :**	2019-07-10T17:14:07.212
**Méthode AutoXecute :**	MBT_AutoX_smart
**Bibliothèque de MSP utilisée:**	Culturomics / f8c211c3-71c5-471b-8a7e-7f6abca59bb9 / 2019-06-28T14:15:05.935, Timone / 29617d84-2a1e-4bf6-a13d-569eecb48f06 / 2018-04-19T13:24:29.884, BDAL / contains 7854 MSPs / e7ef41ca-b750-4d47-9a1c-6c26fa454356 / 2019-02-01T09:48:20.358

Classement (Qualité)	Profil de référence	Score Valeur	Identifiant NCBI
1 (+++)	Bacillus pumilus CSURP4226	2.42	130148166
2 (+++)	Bacillus pumilus CSURP8100	2.35	130148166
3 (+++)	Bacillus pumilus CSURP4105	2.23	130148166
4 (+++)	Bacillus pumilus CSURP4085	2.12	130148166
5 (+++)	Bacillus pumilus 10403329	2.08	133055080
6 (+++)	Bacillus pumilus 10403985	2.07	133055080
7 (+++)	Bacillus pumilus 10403607	2.04	133055080
8 (+++)	Bacillus pumilus 10403987	2.04	133055080
9 (+++)	Bacillus pumilus CSURP6343	2.03	130148166

Tableau des résultats pour analyte 27--suite page suivante

Tableau des résultats pour analyte 27 -- suite de la page précédente			
**Classement (Qualité)**	**Profil de référence**	**Score Valeur**	**Identifiant NCBI**
10 (+++)	Bacillus pumilus CSURP6343	2.03	130148166

Analyte 28

**Nom de l'échantillon:**	C4
**Description de l'échantillon:**	
**ID de l'échantillon:**	C4
**Date/Heure de création de l'échantillon:**	2019-07-10T17:04:20.630
**Type de l'échantillon:**	Échantillon standard
**Méthode de classification :**	MALDI Biotyper MSP Identification Standard Method 1.1
**Méthode de prétraitement :**	BioTyper Preprocessing Standard Method 1.2
**Méthode ACQ :**	D:\Methods\flexControlMethods\MBT_FC.par
**Horodatage ACQ :**	2019-07-10T17:14:34.848
**Méthode AutoXecute :**	MBT_AutoX_smart
**Bibliothèque de MSP utilisée:**	Culturomics / f8c211c3-71c5-471b-8a7e-7f6abca59bb9 / 2019-06-28T14:15:05.935, Timone / 29617d84-2a1e-4bf6-a13d-569eecb48f06 / 2018-04-19T13:24:29.884, BDAL / contains 7854 MSPs / e7ef41ca-b750-4d47-9a1c-6c26fa454356 / 2019-02-01T09:48:20.358

Classement (Qualité)	Profil de référence	Score Valeur	Identifiant NCBI
1 (-)	Bacillus pumilus CSURP6343	1.42	130148166
2 (-)	Bacillus pumilus CSURP6343	1.42	130148166
3 (-)	Bacillus pumilus 10403329	1.40	133055080
4 (-)	Acidaminococcus phocaeensis CSURP4266	1.37	130148166
5 (-)	Bacillus pumilus CSURP4085	1.36	130148166
6 (-)	Hydrogenophaga flava B339 UFL	1.31	65657
7 (-)	Bacillus pumilus CSURP4105	1.31	130148166
8 (-)	Bacillus pumilus 10403987	1.31	133055080
9 (-)	180319-IF2-Thao16-P6485	1.26	130136093

Tableau des résultats pour analyte 28--suite page suivante

Tableau des résultats pour analyte 28 -- suite de la page précédente			
**Classement (Qualité)**	**Profil de référence**	**Score Valeur**	**Identifiant NCBI**
10<br>(-)	Pseudomonas chlororaphis ssp aurantiaca CIP 106718T HAM	1.22	333

Analyte 29

**Nom de l'échantillon:**	C5
**Description de l'échantillon:**	
**ID de l'échantillon:**	C5
**Date/Heure de création de l'échantillon:**	2019-07-10T17:04:20.632
**Type de l'échantillon:**	Échantillon standard
**Méthode de classification :**	MALDI Biotyper MSP Identification Standard Method 1.1
**Méthode de prétraitement :**	BioTyper Preprocessing Standard Method 1.2
**Méthode ACQ :**	D:\Methods\flexControlMethods\MBT_FC.par
**Horodatage ACQ :**	2019-07-10T17:15:09.988
**Méthode AutoXecute :**	MBT_AutoX_smart
**Bibliothèque de MSP utilisée:**	Culturomics / f8c211c3-71c5-471b-8a7e-7f6abca59bb9 / 2019-06-28T14:15:05.935, Timone / 29617d84-2a1e-4bf6-a13d-569eecb48f06 / 2018-04-19T13:24:29.884, BDAL / contains 7854 MSPs / e7ef41ca-b750-4d47-9a1c-6c26fa454356 / 2019-02-01T09:48:20.358

Classement (Qualité)	Profil de référence	Score Valeur	Identifiant NCBI
1 (+++)	Bacillus pumilus CSURP4226	2.11	130148166
2 (+)	Bacillus pumilus CSURP8100	1.98	130148166
3 (+)	Bacillus pumilus CSURP4105	1.94	130148166
4 (+)	Bacillus pumilus 10403987	1.87	133055080
5 (+)	Bacillus pumilus CSURP6343	1.87	130148166
6 (+)	Bacillus pumilus CSURP6343	1.87	130148166
7 (+)	Bacillus pumilus 10403607	1.81	133055080
8 (+)	Bacillus pumilus 10403985	1.79	133055080
9 (+)	Bacillus pumilus 10403990	1.76	133055080

Tableau des résultats pour analyte 29--suite page suivante

Tableau des résultats pour analyte 29 -- suite de la page précédente			
**Classement (Qualité)**	**Profil de référence**	**Score Valeur**	**Identifiant NCBI**
10 (+)	Bacillus pumilus CSURP4085	1.72	130148166

Analyte 30

**Nom de l'échantillon:**	C6
**Description de l'échantillon:**	
**ID de l'échantillon:**	C6
**Date/Heure de création de l'échantillon:**	2019-07-10T17:04:20.633
**Type de l'échantillon:**	Échantillon standard
**Méthode de classification :**	MALDI Biotyper MSP Identification Standard Method 1.1
**Méthode de prétraitement :**	BioTyper Preprocessing Standard Method 1.2
**Méthode ACQ :**	D:\Methods\flexControlMethods\MBT_FC.par
**Horodatage ACQ :**	2019-07-10T17:15:31.012
**Méthode AutoXecute :**	MBT_AutoX_smart
**Bibliothèque de MSP utilisée:**	Culturomics / f8c211c3-71c5-471b-8a7e-7f6abca59bb9 / 2019-06-28T14:15:05.935, Timone / 29617d84-2a1e-4bf6-a13d-569eecb48f06 / 2018-04-19T13:24:29.884, BDAL / contains 7854 MSPs / e7ef41ca-b750-4d47-9a1c-6c26fa454356 / 2019-02-01T09:48:20.358

Classement (Qualité)	Profil de référence	Score Valeur	Identifiant NCBI
1 (+++)	Bacillus pumilus CSURP4226	2.24	130148166
2 (+++)	Bacillus pumilus CSURP8100	2.10	130148166
3 (+++)	Bacillus pumilus CSURP4105	2.01	130148166
4 (+)	Bacillus pumilus CSURP4085	1.97	130148166
5 (+)	Bacillus pumilus 10403987	1.96	133055080
6 (+)	Bacillus pumilus 10403985	1.93	133055080
7 (+)	Bacillus pumilus 10403990	1.92	133055080
8 (+)	Bacillus pumilus CSURP6343	1.83	130148166
9 (+)	Bacillus pumilus CSURP6343	1.83	130148166

Tableau des résultats pour analyte 30--suite page suivante

Tableau des résultats pour analyte 30 -- suite de la page précédente			
**Classement (Qualité)**	**Profil de référence**	**Score Valeur**	**Identifiant NCBI**
10 (+)	Bacillus pumilus 10403329	1.83	133055080

Analyte 31

**Nom de l'échantillon:**	C7
**Description de l'échantillon:**	
**ID de l'échantillon:**	C7
**Date/Heure de création de l'échantillon:**	2019-07-10T17:04:20.635
**Type de l'échantillon:**	Échantillon standard
**Méthode de classification :**	MALDI Biotyper MSP Identification Standard Method 1.1
**Méthode de prétraitement :**	BioTyper Preprocessing Standard Method 1.2
**Méthode ACQ :**	D:\Methods\flexControlMethods\MBT_FC.par
**Horodatage ACQ :**	2019-07-10T17:15:52.117
**Méthode AutoXecute :**	MBT_AutoX_smart
**Bibliothèque de MSP utilisée:**	Culturomics / f8c211c3-71c5-471b-8a7e-7f6abca59bb9 / 2019-06-28T14:15:05.935, Timone / 29617d84-2a1e-4bf6-a13d-569eecb48f06 / 2018-04-19T13:24:29.884, BDAL / contains 7854 MSPs / e7ef41ca-b750-4d47-9a1c-6c26fa454356 / 2019-02-01T09:48:20.358

Classement (Qualité)	Profil de référence	Score Valeur	Identifiant NCBI
1 (+)	Staphylococcus hominis CSURP3985	1.93	130148166
2 (+)	Staphylococcus hominis 18 ESL	1.82	1290
3 (+)	Staphylococcus hominis CSURP2345	1.79	130148166
4 (+)	Staphylococcus hominis CSURP5934	1.78	130148166
5 (+)	Staphylococcus hominis CSURP2340	1.72	130148166
6 (-)	Staphylococcus hominis CSURP5483	1.69	130148166
7 (-)	Staphylococcus hominis CSURP547	1.66	130148166
8 (-)	Staphylococcus hominis ssp hominis DSM 20328T DSM	1.66	145391
9 (-)	Staphylococcus hominis CSURP3146	1.65	130148166

Tableau des résultats pour analyte 31--suite page suivante

Tableau des résultats pour analyte 31 -- suite de la page précédente			
**Classement (Qualité)**	**Profil de référence**	**Score Valeur**	**Identifiant NCBI**
10 (-)	Staphylococcus hominis CSURP292	1.65	130148166

Analyte 32

**Nom de l'échantillon:**	C8
**Description de l'échantillon:**	
**ID de l'échantillon:**	C8
**Date/Heure de création de l'échantillon:**	2019-07-10T17:04:20.636
**Type de l'échantillon:**	Échantillon standard
**Méthode de classification :**	MALDI Biotyper MSP Identification Standard Method 1.1
**Méthode de prétraitement :**	BioTyper Preprocessing Standard Method 1.2
**Méthode ACQ :**	D:\Methods\flexControlMethods\MBT_FC.par
**Horodatage ACQ :**	2019-07-10T17:16:12.936
**Méthode AutoXecute :**	MBT_AutoX_smart
**Bibliothèque de MSP utilisée:**	Culturomics / f8c211c3-71c5-471b-8a7e-7f6abca59bb9 / 2019-06-28T14:15:05.935, Timone / 29617d84-2a1e-4bf6-a13d-569eecb48f06 / 2018-04-19T13:24:29.884, BDAL / contains 7854 MSPs / e7ef41ca-b750-4d47-9a1c-6c26fa454356 / 2019-02-01T09:48:20.358

Classement (Qualité)	Profil de référence	Score Valeur	Identifiant NCBI
1 (+)	Staphylococcus hominis CSURP5483	1.93	130148166
2 (+)	Staphylococcus hominis CSURP3985	1.90	130148166
3 (+)	Staphylococcus hominis CSURP5934	1.85	130148166
4 (+)	Staphylococcus hominis CSURP2345	1.81	130148166
5 (+)	Staphylococcus hominis CSURP4932	1.79	130148166
6 (+)	Staphylococcus hominis CSURP547	1.78	130148166
7 (+)	Staphylococcus hominis 18 ESL	1.78	1290
8 (+)	Staphylococcus hominis CSURP2340	1.72	130148166
9 (-)	Staphylococcus hominis ssp hominis DSM 20328T DSM	1.63	145391

Tableau des résultats pour analyte 32--suite page suivante

Tableau des résultats pour analyte 32 -- suite de la page précédente			
**Classement (Qualité)**	**Profil de référence**	**Score Valeur**	**Identifiant NCBI**
10<br>(-)	Staphylococcus hominis CSURP391	1.59	130148166

Analyte 33

**Nom de l'échantillon:**	C9
**Description de l'échantillon:**	
**ID de l'échantillon:**	C9
**Date/Heure de création de l'échantillon:**	2019-07-10T17:04:20.638
**Type de l'échantillon:**	Échantillon standard
**Méthode de classification :**	MALDI Biotyper MSP Identification Standard Method 1.1
**Méthode de prétraitement :**	BioTyper Preprocessing Standard Method 1.2
**Méthode ACQ :**	D:\Methods\flexControlMethods\MBT_FC.par
**Horodatage ACQ :**	2019-07-10T17:16:33.937
**Méthode AutoXecute :**	MBT_AutoX_smart
**Bibliothèque de MSP utilisée:**	Culturomics / f8c211c3-71c5-471b-8a7e-7f6abca59bb9 / 2019-06-28T14:15:05.935, Timone / 29617d84-2a1e-4bf6-a13d-569eecb48f06 / 2018-04-19T13:24:29.884, BDAL / contains 7854 MSPs / e7ef41ca-b750-4d47-9a1c-6c26fa454356 / 2019-02-01T09:48:20.358

Classement (Qualité)	Profil de référence	Score Valeur	Identifiant NCBI
1 (-)	Clostridium chauvoei 1076_ATCC 10092T BOG	1.50	46867
2 (-)	Lactobacillus paracasei ssp paracasei DSM 46331 DSM	1.34	47714
3 (-)	Staphylococcus hominis 18 ESL	1.30	1290
4 (-)	Marseillicoccus timonensis CSURP2399	1.27	133055080
5 (-)	Staphylococcus aureus ssp aureus DSM 346 DSM	1.25	46170
6 (-)	Clostridium ihumii	1.25	133993714
7 (-)	Clostridium-anorexicamassiliensis	1.25	133993714
8 (-)	Arthrobacter psychrolactophilus DSM 15612T DSM	1.24	92442
9 (-)	Paeniglutamicibacter psychrophenolicus DSM 15454T DSM	1.23	257454

Tableau des résultats pour analyte 33--suite page suivante

Tableau des résultats pour analyte 33 -- suite de la page précédente			
**Classement (Qualité)**	**Profil de référence**	**Score Valeur**	**Identifiant NCBI**
10<br>(-)	Bacteroides fragilis MB_5088_05 THL	1.22	817

Analyte 34

**Nom de l'échantillon:**	C10
**Description de l'échantillon:**	
**ID de l'échantillon:**	C10
**Date/Heure de création de l'échantillon:**	2019-07-10T17:04:20.639
**Type de l'échantillon:**	Échantillon standard
**Méthode de classification :**	MALDI Biotyper MSP Identification Standard Method 1.1
**Méthode de prétraitement :**	BioTyper Preprocessing Standard Method 1.2
**Méthode ACQ :**	D:\Methods\flexControlMethods\MBT_FC.par
**Horodatage ACQ :**	2019-07-10T17:16:54.635
**Méthode AutoXecute :**	MBT_AutoX_smart
**Bibliothèque de MSP utilisée:**	Culturomics / f8c211c3-71c5-471b-8a7e-7f6abca59bb9 / 2019-06-28T14:15:05.935, Timone / 29617d84-2a1e-4bf6-a13d-569eecb48f06 / 2018-04-19T13:24:29.884, BDAL / contains 7854 MSPs / e7ef41ca-b750-4d47-9a1c-6c26fa454356 / 2019-02-01T09:48:20.358

Classement (Qualité)	Profil de référence	Score Valeur	Identifiant NCBI
1 (-)	Sphingomonas sp B616 UFL	1.23	13687
2 (-)	Acidiphilium acidophilum B349 UFL	1.17	76588
3 (-)	Clostridium baratii 1084_ATCC 25782 BOG	1.15	1561
4 (-)	Staphylococcus hominis Mb18788_1 CHB	1.15	1290
5 (-)	Candida mesenterica DSM 70759 DSM	1.14	45568
6 (-)	Sporolactobacillus laevolacticus DSM 442T DSM	1.13	2077
7 (-)	Prevotella veroralis	1.12	133993714
8 (-)	Staphylococcus aureus ssp aureus DSM 346 DSM	1.11	46170
9 (-)	Enterobacter cloacae CSURP1962	1.11	130148166

Tableau des résultats pour analyte 34--suite page suivante

Tableau des résultats pour analyte 34 -- suite de la page précédente			
**Classement (Qualité)**	**Profil de référence**	**Score Valeur**	**Identifiant NCBI**
10<br>(-)	Rhizobium radiobacter B170 UFL	1.08	358

Analyte 35

**Nom de l'échantillon:**	C11
**Description de l'échantillon:**	
**ID de l'échantillon:**	C11
**Date/Heure de création de l'échantillon:**	2019-07-10T17:04:20.641
**Type de l'échantillon:**	Échantillon standard
**Méthode de classification :**	MALDI Biotyper MSP Identification Standard Method 1.1
**Méthode de prétraitement :**	BioTyper Preprocessing Standard Method 1.2
**Méthode ACQ :**	D:\Methods\flexControlMethods\MBT_FC.par
**Horodatage ACQ :**	2019-07-10T17:17:15.933
**Méthode AutoXecute :**	MBT_AutoX_smart
**Bibliothèque de MSP utilisée:**	Culturomics / f8c211c3-71c5-471b-8a7e-7f6abca59bb9 / 2019-06-28T14:15:05.935, Timone / 29617d84-2a1e-4bf6-a13d-569eecb48f06 / 2018-04-19T13:24:29.884, BDAL / contains 7854 MSPs / e7ef41ca-b750-4d47-9a1c-6c26fa454356 / 2019-02-01T09:48:20.358

Classement (Qualité)	Profil de référence	Score Valeur	Identifiant NCBI
1 (+++)	Bacillus pumilus CSURP4085	2.14	130148166
2 (+++)	Bacillus pumilus CSURP8100	2.11	130148166
3 (+)	Bacillus pumilus CSURP4105	1.93	130148166
4 (+)	Bacillus pumilus CSURP4226	1.87	130148166
5 (+)	Bacillus pumilus CSURP6343	1.83	130148166
6 (+)	Bacillus pumilus CSURP6343	1.83	130148166
7 (+)	Bacillus pumilus 10403329	1.79	133055080
8 (-)	Bacillus pumilus 10403607	1.68	133055080
9 (-)	Bacillus pumilus CSURP8210	1.56	130148166

Tableau des résultats pour analyte 35--suite page suivante

Tableau des résultats pour analyte 35 -- suite de la page précédente			
**Classement (Qualité)**	**Profil de référence**	**Score Valeur**	**Identifiant NCBI**
10 (-)	Bacillus pumilus DSM 354 DSM	1.46	1408

Analyte 36

**Nom de l'échantillon:**	C12
**Description de l'échantillon:**	
**ID de l'échantillon:**	C12
**Date/Heure de création de l'échantillon:**	2019-07-10T17:04:20.642
**Type de l'échantillon:**	Échantillon standard
**Méthode de classification :**	MALDI Biotyper MSP Identification Standard Method 1.1
**Méthode de prétraitement :**	BioTyper Preprocessing Standard Method 1.2
**Méthode ACQ :**	D:\Methods\flexControlMethods\MBT_FC.par
**Horodatage ACQ :**	2019-07-10T17:17:37.000
**Méthode AutoXecute :**	MBT_AutoX_smart
**Bibliothèque de MSP utilisée:**	Culturomics / f8c211c3-71c5-471b-8a7e-7f6abca59bb9 / 2019-06-28T14:15:05.935, Timone / 29617d84-2a1e-4bf6-a13d-569eecb48f06 / 2018-04-19T13:24:29.884, BDAL / contains 7854 MSPs / e7ef41ca-b750-4d47-9a1c-6c26fa454356 / 2019-02-01T09:48:20.358

Classement (Qualité)	Profil de référence	Score Valeur	Identifiant NCBI
1 (-)	Burkholderia glumae DSM 9512T HAM	1.15	337
2 (-)	Lactobacillus reuteri CSURP4871	1.10	130148166
3 (-)	Corynebacterium coyleae CSURP8040	1.07	130148166
4 (-)	Clostridium beijerinckii 1011_DSM 552 BOG	1.01	1520
5 (-)	Porphyromonas somerae DSM 23386T DSM	1.00	322095
6 (-)	Corynebacterium striatum PX_21086308 MLD	0.99	43770
7 (-)	Listeria seeligeri CCUG 27802 CCUG	0.97	1640
8 (-)	Lactobacillus reuteri CSURP4870	0.97	130148166
9 (-)	Sanamassiliae timonensis CSURP4683	0.97	130148166

Tableau des résultats pour analyte 36--suite page suivante

Tableau des résultats pour analyte 36 -- suite de la page précédente			
**Classement (Qualité)**	**Profil de référence**	**Score Valeur**	**Identifiant NCBI**
10<br>(-)	Neisseria zoodegmatis CCUG 34523 CCUG	0.97	482

Analyte 37

**Nom de l'échantillon:**	D1
**Description de l'échantillon:**	
**ID de l'échantillon:**	D1
**Date/Heure de création de l'échantillon:**	2019-07-10T17:04:20.643
**Type de l'échantillon:**	Échantillon standard
**Méthode de classification :**	MALDI Biotyper MSP Identification Standard Method 1.1
**Méthode de prétraitement :**	BioTyper Preprocessing Standard Method 1.2
**Méthode ACQ :**	D:\Methods\flexControlMethods\MBT_FC.par
**Horodatage ACQ :**	2019-07-10T17:17:59.140
**Méthode AutoXecute :**	MBT_AutoX_smart
**Bibliothèque de MSP utilisée:**	Culturomics / f8c211c3-71c5-471b-8a7e-7f6abca59bb9 / 2019-06-28T14:15:05.935, Timone / 29617d84-2a1e-4bf6-a13d-569eecb48f06 / 2018-04-19T13:24:29.884, BDAL / contains 7854 MSPs / e7ef41ca-b750-4d47-9a1c-6c26fa454356 / 2019-02-01T09:48:20.358

Classement (Qualité)	Profil de référence	Score Valeur	Identifiant NCBI
1 (+)	Escherichia coli CSURP4799	1.95	130148166
2 (+)	Escherichia coli MB11464_1 CHB	1.89	562
3 (+)	Escherichia coli ATCC 25922 THL	1.88	562
4 (+)	Escherichia coli CSURP1872	1.86	130148166
5 (+)	Escherichia coli CSURP216	1.80	130148166
6 (+)	Escherichia coli CSURP427	1.80	130148166
7 (+)	Escherichia coli CSURP4969	1.80	130148166
8 (+)	Escherichia coli CIP7624 lot 561	1.79	133055080
9 (+)	Escherichia coli CSURP5267	1.79	130148166

Tableau des résultats pour analyte 37--suite page suivante

Tableau des résultats pour analyte 37 -- suite de la page précédente			
**Classement (Qualité)**	**Profil de référence**	**Score Valeur**	**Identifiant NCBI**
10 (+)	Escherichia coli ATCC 25922 CHB	1.78	562

Analyte 38

**Nom de l'échantillon:**	D2
**Description de l'échantillon:**	
**ID de l'échantillon:**	D2
**Date/Heure de création de l'échantillon:**	2019-07-10T17:04:20.644
**Type de l'échantillon:**	Échantillon standard
**Méthode de classification :**	MALDI Biotyper MSP Identification Standard Method 1.1
**Méthode de prétraitement :**	BioTyper Preprocessing Standard Method 1.2
**Méthode ACQ :**	D:\Methods\flexControlMethods\MBT_FC.par
**Horodatage ACQ :**	2019-07-10T17:18:20.433
**Méthode AutoXecute :**	MBT_AutoX_smart
**Bibliothèque de MSP utilisée:**	Culturomics / f8c211c3-71c5-471b-8a7e-7f6abca59bb9 / 2019-06-28T14:15:05.935, Timone / 29617d84-2a1e-4bf6-a13d-569eecb48f06 / 2018-04-19T13:24:29.884, BDAL / contains 7854 MSPs / e7ef41ca-b750-4d47-9a1c-6c26fa454356 / 2019-02-01T09:48:20.358

Classement (Qualité)	Profil de référence	Score Valeur	Identifiant NCBI
1 (+++)	Escherichia coli CSURP4799	2.38	130148166
2 (+++)	Escherichia coli CSURP422	2.38	130148166
3 (+++)	Escherichia coli CSURP415	2.33	130148166
4 (+++)	Escherichia coli CSURP420	2.33	130148166
5 (+++)	Escherichia coli CSURP1872	2.29	130148166
6 (+++)	Escherichia coli CSURP1570	2.28	130148166
7 (+++)	Escherichia coli CSURP399	2.27	130148166
8 (+++)	Escherichia coli CIP7624 lot 561	2.26	133055080
9 (+++)	Escherichia coli CSURP3866	2.25	130148166

Tableau des résultats pour analyte 38--suite page suivante

Tableau des résultats pour analyte 38 -- suite de la page précédente			
**Classement (Qualité)**	**Profil de référence**	**Score Valeur**	**Identifiant NCBI**
10 (+++)	Escherichia coli DH5alpha BRL	2.23	562

Analyte 39

**Nom de l'échantillon:**	D3
**Description de l'échantillon:**	
**ID de l'échantillon:**	D3
**Date/Heure de création de l'échantillon:**	2019-07-10T17:04:20.646
**Type de l'échantillon:**	Échantillon standard
**Méthode de classification :**	MALDI Biotyper MSP Identification Standard Method 1.1
**Méthode de prétraitement :**	BioTyper Preprocessing Standard Method 1.2
**Méthode ACQ :**	D:\Methods\flexControlMethods\MBT_FC.par
**Horodatage ACQ :**	2019-07-10T17:18:41.895
**Méthode AutoXecute :**	MBT_AutoX_smart
**Bibliothèque de MSP utilisée:**	Culturomics / f8c211c3-71c5-471b-8a7e-7f6abca59bb9 / 2019-06-28T14:15:05.935, Timone / 29617d84-2a1e-4bf6-a13d-569eecb48f06 / 2018-04-19T13:24:29.884, BDAL / contains 7854 MSPs / e7ef41ca-b750-4d47-9a1c-6c26fa454356 / 2019-02-01T09:48:20.358

Classement (Qualité)	Profil de référence	Score Valeur	Identifiant NCBI
1 (-)	Bacillus subtilis CSURP291	1.56	130148166
2 (-)	Clostridium chauvoei 1076_ATCC 10092T BOG	1.43	46867
3 (-)	Lactobacillus murinus DSM 20452T DSM	1.29	1622
4 (-)	Lactobacillus mucosae DSM 13345T DSM	1.28	97478
5 (-)	Lactobacillus salivarius DSM 20554 DSM	1.28	1624
6 (-)	Bacillus mojavensis CSURP1524	1.28	130148166
7 (-)	Lactobacillus reuteri DSM 20016T DSM	1.27	1598
8 (-)	Lactobacillus curvatus DSM 20019T DSM	1.26	28038
9 (-)	Methylobacterium radiotolerans B236 UFL	1.26	31998

Tableau des résultats pour analyte 39--suite page suivante

Tableau des résultats pour analyte 39 -- suite de la page précédente			
**Classement (Qualité)**	**Profil de référence**	**Score Valeur**	**Identifiant NCBI**
10<br>(-)	Flavobacterium gelidilacus DSM 15343T HAM	1.24	206041

Analyte 40

**Nom de l'échantillon:**	D4
**Description de l'échantillon:**	
**ID de l'échantillon:**	D4
**Date/Heure de création de l'échantillon:**	2019-07-10T17:04:20.647
**Type de l'échantillon:**	Échantillon standard
**Méthode de classification :**	MALDI Biotyper MSP Identification Standard Method 1.1
**Méthode de prétraitement :**	BioTyper Preprocessing Standard Method 1.2
**Méthode ACQ :**	D:\Methods\flexControlMethods\MBT_FC.par
**Horodatage ACQ :**	2019-07-10T17:19:01.915
**Méthode AutoXecute :**	MBT_AutoX_smart
**Bibliothèque de MSP utilisée:**	Culturomics / f8c211c3-71c5-471b-8a7e-7f6abca59bb9 / 2019-06-28T14:15:05.935, Timone / 29617d84-2a1e-4bf6-a13d-569eecb48f06 / 2018-04-19T13:24:29.884, BDAL / contains 7854 MSPs / e7ef41ca-b750-4d47-9a1c-6c26fa454356 / 2019-02-01T09:48:20.358

Classement (Qualité)	Profil de référence	Score Valeur	Identifiant NCBI
1 (-)	Pichia occidentalis CBS 1910 CBS	1.46	54552
2 (-)	Candida lambica CBS 603 CBS	1.40	53655
3 (-)	Staphylococcus epidermidis ATCC 12228 CHB	1.38	1282
4 (-)	Pseudomonas savastanoi ssp savastanoi LMG 5011 HAM	1.37	29438
5 (-)	Arthrobacter pascens DSM 20545T DSM	1.36	1677
6 (-)	Staphylococcus sciuri ssp sciuri DSM 6671 DSM	1.34	147467
7 (-)	Pseudomonas veronii B561 UFL	1.33	76761
8 (-)	Staphylococcus aureus ssp aureus DSM 11822 DSM	1.32	46170
9 (-)	Exophiala dermatitidis RVE_08I VML	1.31	5970

Tableau des résultats pour analyte 40--suite page suivante

Tableau des résultats pour analyte 40 -- suite de la page précédente			
**Classement (Qualité)**	**Profil de référence**	**Score Valeur**	**Identifiant NCBI**
10<br>(-)	Candida albicans ATCC 10231 VML	1.31	5476

Analyte 41

**Nom de l'échantillon:**	D5
**Description de l'échantillon:**	
**ID de l'échantillon:**	D5
**Date/Heure de création de l'échantillon:**	2019-07-10T17:04:20.648
**Type de l'échantillon:**	Échantillon standard
**Méthode de classification :**	MALDI Biotyper MSP Identification Standard Method 1.1
**Méthode de prétraitement :**	BioTyper Preprocessing Standard Method 1.2
**Méthode ACQ :**	D:\Methods\flexControlMethods\MBT_FC.par
**Horodatage ACQ :**	2019-07-10T17:19:23.005
**Méthode AutoXecute :**	MBT_AutoX_smart
**Bibliothèque de MSP utilisée:**	Culturomics / f8c211c3-71c5-471b-8a7e-7f6abca59bb9 / 2019-06-28T14:15:05.935, Timone / 29617d84-2a1e-4bf6-a13d-569eecb48f06 / 2018-04-19T13:24:29.884, BDAL / contains 7854 MSPs / e7ef41ca-b750-4d47-9a1c-6c26fa454356 / 2019-02-01T09:48:20.358

Classement (Qualité)	Profil de référence	Score Valeur	Identifiant NCBI
1 (+)	Bacillus simplex CSURP5196	1.97	130148166
2 (+)	Bacillus simplex CSURP558	1.86	130148166
3 (+)	Bacillus simplex 110818	1.77	133055080
4 (-)	Bacillus simplex 10109635	1.40	133055080
5 (-)	Bacillus cohnii DSM 6307T DSM	1.37	33932
6 (-)	Sanamassiliae timonensis CSURP5586	1.36	130148166
7 (-)	Bacillus muralis DSM 16288T DSM	1.34	264697
8 (-)	Bacillus jeotgali DSM 18226T DSM	1.30	129985
9 (-)	Pseudomonas resinovorans LMG 2274T HAM	1.28	53412

Tableau des résultats pour analyte 41--suite page suivante

Tableau des résultats pour analyte 41 -- suite de la page précédente			
**Classement (Qualité)**	**Profil de référence**	**Score Valeur**	**Identifiant NCBI**
10<br>(-)	Bacillus simplex CS 206_1aI BRB	1.27	1478

Analyte 42

**Nom de l’échantillon:**	D6
**Description de l’échantillon:**	
**ID de l’échantillon:**	D6
**Date/Heure de création de l’échantillon:**	2019-07-10T17:04:20.650
**Type de l’échantillon:**	Échantillon standard
**Méthode de classification :**	MALDI Biotyper MSP Identification Standard Method 1.1
**Méthode de prétraitement :**	BioTyper Preprocessing Standard Method 1.2
**Méthode ACQ :**	D:\Methods\flexControlMethods\MBT_FC.par
**Horodatage ACQ :**	2019-07-10T17:19:43.674
**Méthode AutoXecute :**	MBT_AutoX_smart
**Bibliothèque de MSP utilisée:**	Culturomics / f8c211c3-71c5-471b-8a7e-7f6abca59bb9 / 2019-06-28T14:15:05.935, Timone / 29617d84-2a1e-4bf6-a13d-569eecb48f06 / 2018-04-19T13:24:29.884, BDAL / contains 7854 MSPs / e7ef41ca-b750-4d47-9a1c-6c26fa454356 / 2019-02-01T09:48:20.358

Classement (Qualité)	Profil de référence	Score Valeur	Identifiant NCBI
1 (+)	Bacillus simplex CSURP558	1.91	130148166
2 (-)	Bacillus simplex 10109635	1.57	133055080
3 (-)	Bacillus muralis DSM 16288T DSM	1.55	264697
4 (-)	Bacillus simplex CS 206_1aI BRB	1.52	1478
5 (-)	Bacillus simplex 110818	1.47	133055080
6 (-)	Bacillus simplex CSURP5196	1.45	130148166
7 (-)	Lactobacillus mali DSM 20444T DSM	1.42	1618
8 (-)	Bacillus indicus DSM 15820T DSM	1.38	246786
9 (-)	Bacillus jeotgali DSM 18226T DSM	1.37	129985

Tableau des résultats pour analyte 42--suite page suivante

Tableau des résultats pour analyte 42 -- suite de la page précédente			
**Classement (Qualité)**	**Profil de référence**	**Score Valeur**	**Identifiant NCBI**
10<br>(-)	Lactobacillus parabuchneri DSM 5707T DSM	1.36	152331

Analyte 43

**Nom de l'échantillon:**	D7
**Description de l'échantillon:**	
**ID de l'échantillon:**	D7
**Date/Heure de création de l'échantillon:**	2019-07-10T17:04:20.651
**Type de l'échantillon:**	Échantillon standard
**Méthode de classification :**	MALDI Biotyper MSP Identification Standard Method 1.1
**Méthode de prétraitement :**	BioTyper Preprocessing Standard Method 1.2
**Méthode ACQ :**	D:\Methods\flexControlMethods\MBT_FC.par
**Horodatage ACQ :**	2019-07-10T17:20:06.028
**Méthode AutoXecute :**	MBT_AutoX_smart
**Bibliothèque de MSP utilisée:**	Culturomics / f8c211c3-71c5-471b-8a7e-7f6abca59bb9 / 2019-06-28T14:15:05.935, Timone / 29617d84-2a1e-4bf6-a13d-569eecb48f06 / 2018-04-19T13:24:29.884, BDAL / contains 7854 MSPs / e7ef41ca-b750-4d47-9a1c-6c26fa454356 / 2019-02-01T09:48:20.358

Classement (Qualité)	Profil de référence	Score Valeur	Identifiant NCBI
1 (+++)	Bacillus pumilus CSURP8100	2.42	130148166
2 (+++)	Bacillus pumilus CSURP4085	2.40	130148166
3 (+++)	Bacillus pumilus CSURP4226	2.29	130148166
4 (+++)	Bacillus pumilus CSURP4105	2.09	130148166
5 (+++)	Bacillus pumilus CSURP6343	2.07	130148166
6 (+++)	Bacillus pumilus CSURP6343	2.07	130148166
7 (+++)	Bacillus pumilus 10403329	2.03	133055080
8 (+++)	Bacillus pumilus 10403607	2.02	133055080
9 (+)	Bacillus pumilus CSURP8210	1.91	130148166

Tableau des résultats pour analyte 43--suite page suivante

Tableau des résultats pour analyte 43 -- suite de la page précédente			
**Classement (Qualité)**	**Profil de référence**	**Score Valeur**	**Identifiant NCBI**
10<br>(+)	Bacillus pumilus 10149151	1.90	133055080

Analyte 44

**Nom de l'échantillon:**	D8
**Description de l'échantillon:**	
**ID de l'échantillon:**	D8
**Date/Heure de création de l'échantillon:**	2019-07-10T17:04:20.653
**Type de l'échantillon:**	Échantillon standard
**Méthode de classification :**	MALDI Biotyper MSP Identification Standard Method 1.1
**Méthode de prétraitement :**	BioTyper Preprocessing Standard Method 1.2
**Méthode ACQ :**	D:\Methods\flexControlMethods\MBT_FC.par
**Horodatage ACQ :**	2019-07-10T17:20:27.304
**Méthode AutoXecute :**	MBT_AutoX_smart
**Bibliothèque de MSP utilisée:**	Culturomics / f8c211c3-71c5-471b-8a7e-7f6abca59bb9 / 2019-06-28T14:15:05.935, Timone / 29617d84-2a1e-4bf6-a13d-569eecb48f06 / 2018-04-19T13:24:29.884, BDAL / contains 7854 MSPs / e7ef41ca-b750-4d47-9a1c-6c26fa454356 / 2019-02-01T09:48:20.358

Classement (Qualité)	Profil de référence	Score Valeur	Identifiant NCBI
1 (+)	Bacillus pumilus CSURP8100	1.75	130148166
2 (+)	Bacillus pumilus CSURP4085	1.73	130148166
3 (-)	Bacillus pumilus 10403206	1.62	133055080
4 (-)	Bacillus pumilus 10403329	1.60	133055080
5 (-)	Bacillus pumilus CSURP6343	1.52	130148166
6 (-)	Bacillus pumilus CSURP6343	1.52	130148166
7 (-)	Bacillus pumilus CSURP4105	1.36	130148166
8 (-)	Staphylococcus aureus CSURP4600	1.34	130148166
9 (-)	Bacillus pumilus 10403985	1.34	133055080

Tableau des résultats pour analyte 44--suite page suivante

Tableau des résultats pour analyte 44 -- suite de la page précédente			
**Classement (Qualité)**	**Profil de référence**	**Score Valeur**	**Identifiant NCBI**
10<br>(-)	Bacillus pumilus 10403751	1.33	133055080

Analyte 45

**Nom de l'échantillon:**	D9
**Description de l'échantillon:**	
**ID de l'échantillon:**	D9
**Date/Heure de création de l'échantillon:**	2019-07-10T17:04:20.654
**Type de l'échantillon:**	Échantillon standard
**Méthode de classification :**	MALDI Biotyper MSP Identification Standard Method 1.1
**Méthode de prétraitement :**	BioTyper Preprocessing Standard Method 1.2
**Méthode ACQ :**	D:\Methods\flexControlMethods\MBT_FC.par
**Horodatage ACQ :**	2019-07-10T17:20:48.673
**Méthode AutoXecute :**	MBT_AutoX_smart
**Bibliothèque de MSP utilisée:**	Culturomics / f8c211c3-71c5-471b-8a7e-7f6abca59bb9 / 2019-06-28T14:15:05.935, Timone / 29617d84-2a1e-4bf6-a13d-569eecb48f06 / 2018-04-19T13:24:29.884, BDAL / contains 7854 MSPs / e7ef41ca-b750-4d47-9a1c-6c26fa454356 / 2019-02-01T09:48:20.358

Classement (Qualité)	Profil de référence	Score Valeur	Identifiant NCBI
1 (+++)	Bacillus pumilus CSURP8100	2.36	130148166
2 (+++)	Bacillus pumilus CSURP4226	2.29	130148166
3 (+++)	Bacillus pumilus 10403329	2.15	133055080
4 (+++)	Bacillus pumilus CSURP4085	2.12	130148166
5 (+++)	Bacillus pumilus 10403987	2.10	133055080
6 (+++)	Bacillus pumilus 10403607	2.09	133055080
7 (+++)	Bacillus pumilus 10403990	2.07	133055080
8 (+++)	Bacillus pumilus 10403985	2.05	133055080
9 (+++)	Bacillus pumilus CSURP4105	2.05	130148166

Tableau des résultats pour analyte 45--suite page suivante

Tableau des résultats pour analyte 45 -- suite de la page précédente			
**Classement (Qualité)**	**Profil de référence**	**Score Valeur**	**Identifiant NCBI**
10 (+++)	Bacillus pumilus 10403206	2.03	133055080

Analyte 46

**Nom de l'échantillon:**	D10
**Description de l'échantillon:**	
**ID de l'échantillon:**	D10
**Date/Heure de création de l'échantillon:**	2019-07-10T17:04:20.656
**Type de l'échantillon:**	Échantillon standard
**Méthode de classification :**	MALDI Biotyper MSP Identification Standard Method 1.1
**Méthode de prétraitement :**	BioTyper Preprocessing Standard Method 1.2
**Méthode ACQ :**	D:\Methods\flexControlMethods\MBT_FC.par
**Horodatage ACQ :**	2019-07-10T17:21:09.539
**Méthode AutoXecute :**	MBT_AutoX_smart
**Bibliothèque de MSP utilisée:**	Culturomics / f8c211c3-71c5-471b-8a7e-7f6abca59bb9 / 2019-06-28T14:15:05.935, Timone / 29617d84-2a1e-4bf6-a13d-569eecb48f06 / 2018-04-19T13:24:29.884, BDAL / contains 7854 MSPs / e7ef41ca-b750-4d47-9a1c-6c26fa454356 / 2019-02-01T09:48:20.358

Classement (Qualité)	Profil de référence	Score Valeur	Identifiant NCBI
1 (+++)	Bacillus pumilus CSURP4226	2.23	130148166
2 (+++)	Bacillus pumilus CSURP8100	2.16	130148166
3 (+++)	Bacillus pumilus CSURP4085	2.10	130148166
4 (+)	Bacillus pumilus CSURP6343	1.93	130148166
5 (+)	Bacillus pumilus CSURP6343	1.93	130148166
6 (+)	Bacillus pumilus 10403329	1.92	133055080
7 (+)	Bacillus pumilus 10403985	1.91	133055080
8 (+)	Bacillus pumilus CSURP4105	1.91	130148166
9 (+)	Bacillus pumilus 10403751	1.89	133055080

Tableau des résultats pour analyte 46--suite page suivante

Tableau des résultats pour analyte 46 -- suite de la page précédente			
**Classement (Qualité)**	**Profil de référence**	**Score Valeur**	**Identifiant NCBI**
10 (+)	Bacillus pumilus 10403607	1.86	133055080

Analyte 47

**Nom de l'échantillon:**	D11
**Description de l'échantillon:**	
**ID de l'échantillon:**	D11
**Date/Heure de création de l'échantillon:**	2019-07-10T17:04:20.658
**Type de l'échantillon:**	Échantillon standard
**Méthode de classification :**	MALDI Biotyper MSP Identification Standard Method 1.1
**Méthode de prétraitement :**	BioTyper Preprocessing Standard Method 1.2
**Méthode ACQ :**	D:\Methods\flexControlMethods\MBT_FC.par
**Horodatage ACQ :**	2019-07-10T17:21:31.309
**Méthode AutoXecute :**	MBT_AutoX_smart
**Bibliothèque de MSP utilisée:**	Culturomics / f8c211c3-71c5-471b-8a7e-7f6abca59bb9 / 2019-06-28T14:15:05.935, Timone / 29617d84-2a1e-4bf6-a13d-569eecb48f06 / 2018-04-19T13:24:29.884, BDAL / contains 7854 MSPs / e7ef41ca-b750-4d47-9a1c-6c26fa454356 / 2019-02-01T09:48:20.358

Classement (Qualité)	Profil de référence	Score Valeur	Identifiant NCBI
1 (-)	Clostridium novyi 1082_ATCC 17861T BOG	1.38	1542
2 (-)	Paeniglutamicibacter sulfureus B571 UFL	1.33	43666
3 (-)	Pseudomonas taetrolens LMG 2336T HAM	1.32	47884
4 (-)	Legionella anisa HL04042021	1.31	133055080
5 (-)	Actinocorallia libanotica B246 UFL	1.30	46162
6 (-)	Sinomonas atrocyanea DSM 20127T DSM	1.30	37927
7 (-)	Legionella gormanii ATCC 33297	1.27	133055080
8 (-)	Clostridium cochlearium 1080_ATCC 17794T BOG	1.26	1494
9 (-)	Staphylococcus simulans DSM 20324 DSM	1.25	1286

Tableau des résultats pour analyte 47--suite page suivante

Tableau des résultats pour analyte 47 -- suite de la page précédente			
**Classement (Qualité)**	**Profil de référence**	**Score Valeur**	**Identifiant NCBI**
10<br>(-)	Proteus mirabilis (PX) 22086112 MLD	1.25	584

Analyte 48

**Nom de l'échantillon:**	D12
**Description de l'échantillon:**	
**ID de l'échantillon:**	D12
**Date/Heure de création de l'échantillon:**	2019-07-10T17:04:20.659
**Type de l'échantillon:**	Échantillon standard
**Méthode de classification :**	MALDI Biotyper MSP Identification Standard Method 1.1
**Méthode de prétraitement :**	BioTyper Preprocessing Standard Method 1.2
**Méthode ACQ :**	D:\Methods\flexControlMethods\MBT_FC.par
**Horodatage ACQ :**	2019-07-10T17:21:52.855
**Méthode AutoXecute :**	MBT_AutoX_smart
**Bibliothèque de MSP utilisée:**	Culturomics / f8c211c3-71c5-471b-8a7e-7f6abca59bb9 / 2019-06-28T14:15:05.935, Timone / 29617d84-2a1e-4bf6-a13d-569eecb48f06 / 2018-04-19T13:24:29.884, BDAL / contains 7854 MSPs / e7ef41ca-b750-4d47-9a1c-6c26fa454356 / 2019-02-01T09:48:20.358

Classement (Qualité)	Profil de référence	Score Valeur	Identifiant NCBI
1 (+)	Bacillus simplex CSURP558	1.86	130148166
2 (-)	Bacillus simplex CSURP5196	1.53	130148166
3 (-)	Bacillus muralis DSM 16288T DSM	1.47	264697
4 (-)	Bacillus simplex 110818	1.44	133055080
5 (-)	Bacillus simplex 10109635	1.40	133055080
6 (-)	Lactobacillus satsumensis DSM 16230T DSM	1.33	259059
7 (-)	Bacillus megaterium DSM 32T DSM	1.31	1404
8 (-)	Bacillus subtilis ssp spizizenii DSM 15029T DSM	1.30	96241
9 (-)	Bacillus endophyticus DSM 13796T DSM	1.28	135735

Tableau des résultats pour analyte 48--suite page suivante

Tableau des résultats pour analyte 48 -- suite de la page précédente			
**Classement (Qualité)**	**Profil de référence**	**Score Valeur**	**Identifiant NCBI**
10<br>(-)	Corynebacterium sp 10054746	1.26	133055080

Analyte 49

**Nom de l'échantillon:**	E1
**Description de l'échantillon:**	
**ID de l'échantillon:**	E1
**Date/Heure de création de l'échantillon:**	2019-07-10T17:04:20.661
**Type de l'échantillon:**	Échantillon standard
**Méthode de classification :**	MALDI Biotyper MSP Identification Standard Method 1.1
**Méthode de prétraitement :**	BioTyper Preprocessing Standard Method 1.2
**Méthode ACQ :**	D:\Methods\flexControlMethods\MBT_FC.par
**Horodatage ACQ :**	2019-07-10T17:22:12.754
**Méthode AutoXecute :**	MBT_AutoX_smart
**Bibliothèque de MSP utilisée:**	Culturomics / f8c211c3-71c5-471b-8a7e-7f6abca59bb9 / 2019-06-28T14:15:05.935, Timone / 29617d84-2a1e-4bf6-a13d-569eecb48f06 / 2018-04-19T13:24:29.884, BDAL / contains 7854 MSPs / e7ef41ca-b750-4d47-9a1c-6c26fa454356 / 2019-02-01T09:48:20.358

Classement (Qualité)	Profil de référence	Score Valeur	Identifiant NCBI
1 (+)	Bacillus pumilus CSURP4226	1.99	130148166
2 (+)	Bacillus pumilus CSURP4085	1.92	130148166
3 (+)	Bacillus pumilus CSURP8100	1.90	130148166
4 (+)	Bacillus pumilus 10403329	1.70	133055080
5 (-)	Bacillus pumilus 10403987	1.67	133055080
6 (-)	Bacillus pumilus 10403985	1.63	133055080
7 (-)	Bacillus pumilus 10403206	1.60	133055080
8 (-)	Bacillus pumilus 10403607	1.60	133055080
9 (-)	Bacillus pumilus 10403751	1.55	133055080

Tableau des résultats pour analyte 49--suite page suivante

Tableau des résultats pour analyte 49 -- suite de la page précédente			
**Classement (Qualité)**	**Profil de référence**	**Score Valeur**	**Identifiant NCBI**
10<br>(-)	Bacillus pumilus 10403990	1.55	133055080

Analyte 50

**Nom de l'échantillon:**	E2
**Description de l'échantillon:**	
**ID de l'échantillon:**	E2
**Date/Heure de création de l'échantillon:**	2019-07-10T17:04:20.663
**Type de l'échantillon:**	Échantillon standard
**Méthode de classification :**	MALDI Biotyper MSP Identification Standard Method 1.1
**Méthode de prétraitement :**	BioTyper Preprocessing Standard Method 1.2
**Méthode ACQ :**	D:\Methods\flexControlMethods\MBT_FC.par
**Horodatage ACQ :**	2019-07-10T17:22:33.271
**Méthode AutoXecute :**	MBT_AutoX_smart
**Bibliothèque de MSP utilisée:**	Culturomics / f8c211c3-71c5-471b-8a7e-7f6abca59bb9 / 2019-06-28T14:15:05.935, Timone / 29617d84-2a1e-4bf6-a13d-569eecb48f06 / 2018-04-19T13:24:29.884, BDAL / contains 7854 MSPs / e7ef41ca-b750-4d47-9a1c-6c26fa454356 / 2019-02-01T09:48:20.358

Classement (Qualité)	Profil de référence	Score Valeur	Identifiant NCBI
1 (+++)	Bacillus pumilus CSURP8100	2.14	130148166
2 (+++)	Bacillus pumilus CSURP4085	2.01	130148166
3 (+)	Bacillus pumilus CSURP6343	1.87	130148166
4 (+)	Bacillus pumilus CSURP6343	1.87	130148166
5 (+)	Bacillus pumilus CSURP4226	1.84	130148166
6 (+)	Bacillus pumilus 10149151	1.83	133055080
7 (+)	Bacillus pumilus 10403329	1.82	133055080
8 (+)	Bacillus pumilus 10403987	1.78	133055080
9 (+)	Bacillus pumilus 10403607	1.74	133055080

Tableau des résultats pour analyte 50--suite page suivante

Tableau des résultats pour analyte 50 -- suite de la page précédente			
**Classement (Qualité)**	**Profil de référence**	**Score Valeur**	**Identifiant NCBI**
10<br>(-)	Bacillus pumilus 10403751	1.69	133055080

Analyte 51

**Nom de l'échantillon:**	E3
**Description de l'échantillon:**	
**ID de l'échantillon:**	E3
**Date/Heure de création de l'échantillon:**	2019-07-10T17:04:20.664
**Type de l'échantillon:**	Échantillon standard
**Méthode de classification :**	MALDI Biotyper MSP Identification Standard Method 1.1
**Méthode de prétraitement :**	BioTyper Preprocessing Standard Method 1.2
**Méthode ACQ :**	D:\Methods\flexControlMethods\MBT_FC.par
**Horodatage ACQ :**	2019-07-10T17:22:54.401
**Méthode AutoXecute :**	MBT_AutoX_smart
**Bibliothèque de MSP utilisée:**	Culturomics / f8c211c3-71c5-471b-8a7e-7f6abca59bb9 / 2019-06-28T14:15:05.935, Timone / 29617d84-2a1e-4bf6-a13d-569eecb48f06 / 2018-04-19T13:24:29.884, BDAL / contains 7854 MSPs / e7ef41ca-b750-4d47-9a1c-6c26fa454356 / 2019-02-01T09:48:20.358

Classement (Qualité)	Profil de référence	Score Valeur	Identifiant NCBI
1 (+++)	Bacillus pumilus CSURP8100	2.31	130148166
2 (+)	Bacillus pumilus 10403987	1.99	133055080
3 (+)	Bacillus pumilus 10403329	1.98	133055080
4 (+)	Bacillus pumilus CSURP6343	1.89	130148166
5 (+)	Bacillus pumilus CSURP6343	1.89	130148166
6 (+)	Bacillus pumilus 10403607	1.85	133055080
7 (+)	Bacillus pumilus CSURP4105	1.83	130148166
8 (+)	Bacillus pumilus 10403985	1.83	133055080
9 (+)	Bacillus pumilus CSURP3862	1.76	130148166

Tableau des résultats pour analyte 51--suite page suivante

Classement (Qualité)	Profil de référence	Score Valeur	Identifiant NCBI
Tableau des résultats pour analyte 51 -- suite de la page précédente			
10 (+)	Bacillus pumilus 10403990	1.75	133055080

Analyte 52

**Nom de l'échantillon:**	E4
**Description de l'échantillon:**	
**ID de l'échantillon:**	E4
**Date/Heure de création de l'échantillon:**	2019-07-10T17:04:20.666
**Type de l'échantillon:**	Échantillon standard
**Méthode de classification :**	MALDI Biotyper MSP Identification Standard Method 1.1
**Méthode de prétraitement :**	BioTyper Preprocessing Standard Method 1.2
**Méthode ACQ :**	D:\Methods\flexControlMethods\MBT_FC.par
**Horodatage ACQ :**	2019-07-10T17:23:15.480
**Méthode AutoXecute :**	MBT_AutoX_smart
**Bibliothèque de MSP utilisée:**	Culturomics / f8c211c3-71c5-471b-8a7e-7f6abca59bb9 / 2019-06-28T14:15:05.935, Timone / 29617d84-2a1e-4bf6-a13d-569eecb48f06 / 2018-04-19T13:24:29.884, BDAL / contains 7854 MSPs / e7ef41ca-b750-4d47-9a1c-6c26fa454356 / 2019-02-01T09:48:20.358

Classement (Qualité)	Profil de référence	Score Valeur	Identifiant NCBI
1 (+++)	Bacillus pumilus CSURP4226	2.39	130148166
2 (+++)	Bacillus pumilus CSURP8100	2.37	130148166
3 (+++)	Bacillus pumilus CSURP4085	2.18	130148166
4 (+++)	Bacillus pumilus CSURP4105	2.09	130148166
5 (+++)	Bacillus pumilus CSURP3862	2.01	130148166
6 (+)	Bacillus pumilus CSURP6343	1.98	130148166
7 (+)	Bacillus pumilus CSURP6343	1.98	130148166
8 (+)	Bacillus pumilus CSURP8210	1.95	130148166
9 (+)	Bacillus pumilus 10403607	1.93	133055080

Tableau des résultats pour analyte 52--suite page suivante

Tableau des résultats pour analyte 52 -- suite de la page précédente			
**Classement (Qualité)**	**Profil de référence**	**Score Valeur**	**Identifiant NCBI**
10<br>(+)	Bacillus pumilus 10403987	1.91	133055080

Analyte 53

**Nom de l'échantillon:**	E5
**Description de l'échantillon:**	
**ID de l'échantillon:**	E5
**Date/Heure de création de l'échantillon:**	2019-07-10T17:04:20.668
**Type de l'échantillon:**	Échantillon standard
**Méthode de classification :**	MALDI Biotyper MSP Identification Standard Method 1.1
**Méthode de prétraitement :**	BioTyper Preprocessing Standard Method 1.2
**Méthode ACQ :**	D:\Methods\flexControlMethods\MBT_FC.par
**Horodatage ACQ :**	2019-07-10T17:23:36.393
**Méthode AutoXecute :**	MBT_AutoX_smart
**Bibliothèque de MSP utilisée:**	Culturomics / f8c211c3-71c5-471b-8a7e-7f6abca59bb9 / 2019-06-28T14:15:05.935, Timone / 29617d84-2a1e-4bf6-a13d-569eecb48f06 / 2018-04-19T13:24:29.884, BDAL / contains 7854 MSPs / e7ef41ca-b750-4d47-9a1c-6c26fa454356 / 2019-02-01T09:48:20.358

Classement (Qualité)	Profil de référence	Score Valeur	Identifiant NCBI
1 (+++)	Bacillus pumilus CSURP4226	2.31	130148166
2 (+++)	Bacillus pumilus CSURP8100	2.27	130148166
3 (+++)	Bacillus pumilus CSURP4105	2.06	130148166
4 (+)	Bacillus pumilus 10403987	1.97	133055080
5 (+)	Bacillus pumilus 10403329	1.96	133055080
6 (+)	Bacillus pumilus CSURP4085	1.92	130148166
7 (+)	Bacillus pumilus 10403607	1.89	133055080
8 (+)	Bacillus pumilus CSURP6343	1.87	130148166
9 (+)	Bacillus pumilus CSURP6343	1.87	130148166

Tableau des résultats pour analyte 53--suite page suivante

Tableau des résultats pour analyte 53 -- suite de la page précédente			
**Classement (Qualité)**	**Profil de référence**	**Score Valeur**	**Identifiant NCBI**
10<br>(+)	Bacillus pumilus 10403985	1.82	133055080

Analyte 54

**Nom de l’échantillon:**	E6
**Description de l’échantillon:**	
**ID de l’échantillon:**	E6
**Date/Heure de création de l’échantillon:**	2019-07-10T17:04:20.670
**Type de l’échantillon:**	Échantillon standard
**Méthode de classification :**	MALDI Biotyper MSP Identification Standard Method 1.1
**Méthode de prétraitement :**	BioTyper Preprocessing Standard Method 1.2
**Méthode ACQ :**	D:\Methods\flexControlMethods\MBT_FC.par
**Horodatage ACQ :**	2019-07-10T17:23:57.614
**Méthode AutoXecute :**	MBT_AutoX_smart
**Bibliothèque de MSP utilisée:**	Culturomics / f8c211c3-71c5-471b-8a7e-7f6abca59bb9 / 2019-06-28T14:15:05.935, Timone / 29617d84-2a1e-4bf6-a13d-569eecb48f06 / 2018-04-19T13:24:29.884, BDAL / contains 7854 MSPs / e7ef41ca-b750-4d47-9a1c-6c26fa454356 / 2019-02-01T09:48:20.358

Classement (Qualité)	Profil de référence	Score Valeur	Identifiant NCBI
1 (+)	Bacillus pumilus CSURP4226	1.75	130148166
2 (+)	Bacillus pumilus CSURP8100	1.72	130148166
3 (+)	Bacillus pumilus 10403987	1.71	133055080
4 (-)	Bacillus pumilus 10403990	1.67	133055080
5 (-)	Bacillus pumilus 10403607	1.64	133055080
6 (-)	Bacillus pumilus 10403985	1.60	133055080
7 (-)	Bacillus pumilus 10403751	1.58	133055080
8 (-)	Bacillus pumilus 10403329	1.54	133055080
9 (-)	Myroides odoratus CCM 3297 CCM	1.47	256

Tableau des résultats pour analyte 54--suite page suivante

Tableau des résultats pour analyte 54 -- suite de la page précédente			
**Classement (Qualité)**	**Profil de référence**	**Score Valeur**	**Identifiant NCBI**
10<br>(-)	Bacillus pumilus CSURP4085	1.45	130148166

Analyte 55

**Nom de l'échantillon:**	E7
**Description de l'échantillon:**	
**ID de l'échantillon:**	E7
**Date/Heure de création de l'échantillon:**	2019-07-10T17:04:20.671
**Type de l'échantillon:**	Échantillon standard
**Méthode de classification :**	MALDI Biotyper MSP Identification Standard Method 1.1
**Méthode de prétraitement :**	BioTyper Preprocessing Standard Method 1.2
**Méthode ACQ :**	D:\Methods\flexControlMethods\MBT_FC.par
**Horodatage ACQ :**	2019-07-10T17:24:19.317
**Méthode AutoXecute :**	MBT_AutoX_smart
**Bibliothèque de MSP utilisée:**	Culturomics / f8c211c3-71c5-471b-8a7e-7f6abca59bb9 / 2019-06-28T14:15:05.935, Timone / 29617d84-2a1e-4bf6-a13d-569eecb48f06 / 2018-04-19T13:24:29.884, BDAL / contains 7854 MSPs / e7ef41ca-b750-4d47-9a1c-6c26fa454356 / 2019-02-01T09:48:20.358

Classement (Qualité)	Profil de référence	Score Valeur	Identifiant NCBI
1 (+)	Bacillus pumilus 10403329	1.74	133055080
2 (+)	Bacillus pumilus 10403206	1.71	133055080
3 (-)	Bacillus pumilus CSURP4085	1.51	130148166
4 (-)	Bacillus pumilus CSURP8100	1.49	130148166
5 (-)	Bacillus pumilus CSURP6343	1.45	130148166
6 (-)	Bacillus pumilus CSURP6343	1.45	130148166
7 (-)	Streptococcus agalactiae CSURP4868	1.32	130148166
8 (-)	Stenotrophomonas maltophilia 10942 CHB	1.25	40324
9 (-)	Bacillus pumilus CSURP4105	1.25	130148166

Tableau des résultats pour analyte 55--suite page suivante

Tableau des résultats pour analyte 55 -- suite de la page précédente			
**Classement (Qualité)**	**Profil de référence**	**Score Valeur**	**Identifiant NCBI**
10<br>(-)	Bacillus pumilus CSURP4226	1.24	130148166

Analyte 56

**Nom de l'échantillon:**	E8
**Description de l'échantillon:**	
**ID de l'échantillon:**	E8
**Date/Heure de création de l'échantillon:**	2019-07-10T17:04:20.673
**Type de l'échantillon:**	Échantillon standard
**Méthode de classification :**	MALDI Biotyper MSP Identification Standard Method 1.1
**Méthode de prétraitement :**	BioTyper Preprocessing Standard Method 1.2
**Méthode ACQ :**	D:\Methods\flexControlMethods\MBT_FC.par
**Horodatage ACQ :**	2019-07-10T17:24:38.291
**Méthode AutoXecute :**	MBT_AutoX_smart
**Bibliothèque de MSP utilisée:**	Culturomics / f8c211c3-71c5-471b-8a7e-7f6abca59bb9 / 2019-06-28T14:15:05.935, Timone / 29617d84-2a1e-4bf6-a13d-569eecb48f06 / 2018-04-19T13:24:29.884, BDAL / contains 7854 MSPs / e7ef41ca-b750-4d47-9a1c-6c26fa454356 / 2019-02-01T09:48:20.358

Classement (Qualité)	Profil de référence	Score Valeur	Identifiant NCBI
1 (+++)	Bacillus pumilus CSURP8100	2.40	130148166
2 (+++)	Bacillus pumilus CSURP4226	2.36	130148166
3 (+++)	Bacillus pumilus 10403329	2.12	133055080
4 (+++)	Bacillus pumilus CSURP4105	2.08	130148166
5 (+++)	Bacillus pumilus 10403607	2.04	133055080
6 (+)	Bacillus pumilus CSURP6343	1.95	130148166
7 (+)	Bacillus pumilus CSURP6343	1.95	130148166
8 (+)	Bacillus pumilus 10403990	1.76	133055080
9 (+)	Bacillus pumilus CSURP3862	1.73	130148166

Tableau des résultats pour analyte 56--suite page suivante

Tableau des résultats pour analyte 56 -- suite de la page précédente			
**Classement (Qualité)**	**Profil de référence**	**Score Valeur**	**Identifiant NCBI**
10<br>(-)	Bacillus pumilus 10149151	1.68	133055080

Analyte 57

**Nom de l'échantillon:**	E9
**Description de l'échantillon:**	
**ID de l'échantillon:**	E9
**Date/Heure de création de l'échantillon:**	2019-07-10T17:04:20.675
**Type de l'échantillon:**	Échantillon standard
**Méthode de classification :**	MALDI Biotyper MSP Identification Standard Method 1.1
**Méthode de prétraitement :**	BioTyper Preprocessing Standard Method 1.2
**Méthode ACQ :**	D:\Methods\flexControlMethods\MBT_FC.par
**Horodatage ACQ :**	2019-07-10T17:24:59.407
**Méthode AutoXecute :**	MBT_AutoX_smart
**Bibliothèque de MSP utilisée:**	Culturomics / f8c211c3-71c5-471b-8a7e-7f6abca59bb9 / 2019-06-28T14:15:05.935, Timone / 29617d84-2a1e-4bf6-a13d-569eecb48f06 / 2018-04-19T13:24:29.884, BDAL / contains 7854 MSPs / e7ef41ca-b750-4d47-9a1c-6c26fa454356 / 2019-02-01T09:48:20.358

Classement (Qualité)	Profil de référence	Score Valeur	Identifiant NCBI
1 (+++)	Bacillus pumilus CSURP8100	2.48	130148166
2 (+++)	Bacillus pumilus CSURP4085	2.24	130148166
3 (+++)	Bacillus pumilus CSURP4226	2.22	130148166
4 (+++)	Bacillus pumilus CSURP4105	2.14	130148166
5 (+)	Bacillus pumilus CSURP6343	1.98	130148166
6 (+)	Bacillus pumilus CSURP6343	1.98	130148166
7 (+)	Bacillus pumilus 10403607	1.96	133055080
8 (+)	Bacillus pumilus CSURP8210	1.90	130148166
9 (+)	Bacillus pumilus 10403329	1.89	133055080

Tableau des résultats pour analyte 57--suite page suivante

Tableau des résultats pour analyte 57 -- suite de la page précédente			
**Classement (Qualité)**	**Profil de référence**	**Score Valeur**	**Identifiant NCBI**
10<br>(+)	Bacillus pumilus 10403206	1.87	133055080

Analyte 58

**Nom de l'échantillon:**	E10
**Description de l'échantillon:**	
**ID de l'échantillon:**	E10
**Date/Heure de création de l'échantillon:**	2019-07-10T17:04:20.677
**Type de l'échantillon:**	Échantillon standard
**Méthode de classification :**	MALDI Biotyper MSP Identification Standard Method 1.1
**Méthode de prétraitement :**	BioTyper Preprocessing Standard Method 1.2
**Méthode ACQ :**	D:\Methods\flexControlMethods\MBT_FC.par
**Horodatage ACQ :**	2019-07-10T17:25:20.060
**Méthode AutoXecute :**	MBT_AutoX_smart
**Bibliothèque de MSP utilisée:**	Culturomics / f8c211c3-71c5-471b-8a7e-7f6abca59bb9 / 2019-06-28T14:15:05.935, Timone / 29617d84-2a1e-4bf6-a13d-569eecb48f06 / 2018-04-19T13:24:29.884, BDAL / contains 7854 MSPs / e7ef41ca-b750-4d47-9a1c-6c26fa454356 / 2019-02-01T09:48:20.358

Classement (Qualité)	Profil de référence	Score Valeur	Identifiant NCBI
1 (+++)	Bacillus pumilus CSURP4226	2.32	130148166
2 (+++)	Bacillus pumilus CSURP8100	2.25	130148166
3 (+++)	Bacillus pumilus CSURP4105	2.07	130148166
4 (+++)	Bacillus pumilus 10403329	2.06	133055080
5 (+++)	Bacillus pumilus 10403607	2.04	133055080
6 (+++)	Bacillus pumilus 10403990	2.00	133055080
7 (+)	Bacillus pumilus 10403985	1.98	133055080
8 (+)	Bacillus pumilus 10403987	1.94	133055080
9 (+)	Bacillus pumilus CSURP6343	1.92	130148166

Tableau des résultats pour analyte 58--suite page suivante

Tableau des résultats pour analyte 58 -- suite de la page précédente			
**Classement (Qualité)**	**Profil de référence**	**Score Valeur**	**Identifiant NCBI**
10<br>(+)	Bacillus pumilus CSURP6343	1.92	130148166

Analyte 59

**Nom de l'échantillon:**	E11
**Description de l'échantillon:**	
**ID de l'échantillon:**	E11
**Date/Heure de création de l'échantillon:**	2019-07-10T17:04:20.679
**Type de l'échantillon:**	Échantillon standard
**Méthode de classification :**	MALDI Biotyper MSP Identification Standard Method 1.1
**Méthode de prétraitement :**	BioTyper Preprocessing Standard Method 1.2
**Méthode ACQ :**	D:\Methods\flexControlMethods\MBT_FC.par
**Horodatage ACQ :**	2019-07-10T17:25:41.264
**Méthode AutoXecute :**	MBT_AutoX_smart
**Bibliothèque de MSP utilisée:**	Culturomics / f8c211c3-71c5-471b-8a7e-7f6abca59bb9 / 2019-06-28T14:15:05.935, Timone / 29617d84-2a1e-4bf6-a13d-569eecb48f06 / 2018-04-19T13:24:29.884, BDAL / contains 7854 MSPs / e7ef41ca-b750-4d47-9a1c-6c26fa454356 / 2019-02-01T09:48:20.358

Classement (Qualité)	Profil de référence	Score Valeur	Identifiant NCBI
1 (-)	Bacillus pumilus 10403329	1.63	133055080
2 (-)	Bacillus pumilus 10403987	1.61	133055080
3 (-)	Bacillus pumilus 10403607	1.60	133055080
4 (-)	Bacillus pumilus 10403985	1.57	133055080
5 (-)	Bacillus pumilus 10403751	1.56	133055080
6 (-)	Bacillus pumilus CSURP4226	1.55	130148166
7 (-)	Bacillus pumilus CSURP8100	1.51	130148166
8 (-)	Bacillus pumilus 10403990	1.50	133055080
9 (-)	Bacillus pumilus 10403206	1.43	133055080

Tableau des résultats pour analyte 59--suite page suivante

Tableau des résultats pour analyte 59 -- suite de la page précédente			
**Classement (Qualité)**	**Profil de référence**	**Score Valeur**	**Identifiant NCBI**
10<br>(-)	Bacillus pumilus CSURP4085	1.35	130148166

Analyte 60

**Nom de l'échantillon:**	E12
**Description de l'échantillon:**	
**ID de l'échantillon:**	E12
**Date/Heure de création de l'échantillon:**	2019-07-10T17:04:20.681
**Type de l'échantillon:**	Échantillon standard
**Méthode de classification :**	MALDI Biotyper MSP Identification Standard Method 1.1
**Méthode de prétraitement :**	BioTyper Preprocessing Standard Method 1.2
**Méthode ACQ :**	D:\Methods\flexControlMethods\MBT_FC.par
**Horodatage ACQ :**	2019-07-10T17:26:02.572
**Méthode AutoXecute :**	MBT_AutoX_smart
**Bibliothèque de MSP utilisée:**	Culturomics / f8c211c3-71c5-471b-8a7e-7f6abca59bb9 / 2019-06-28T14:15:05.935, Timone / 29617d84-2a1e-4bf6-a13d-569eecb48f06 / 2018-04-19T13:24:29.884, BDAL / contains 7854 MSPs / e7ef41ca-b750-4d47-9a1c-6c26fa454356 / 2019-02-01T09:48:20.358

Classement (Qualité)	Profil de référence	Score Valeur	Identifiant NCBI
1 (+++)	Bacillus pumilus CSURP8100	2.18	130148166
2 (+++)	Bacillus pumilus CSURP4085	2.07	130148166
3 (+)	Bacillus pumilus CSURP4226	1.95	130148166
4 (+)	Bacillus pumilus CSURP4105	1.89	130148166
5 (+)	Bacillus pumilus CSURP6343	1.82	130148166
6 (+)	Bacillus pumilus CSURP6343	1.82	130148166
7 (+)	Bacillus pumilus 10403206	1.73	133055080
8 (-)	Bacillus pumilus 10403329	1.66	133055080
9 (-)	Bacillus pumilus 10403607	1.56	133055080

Tableau des résultats pour analyte 60--suite page suivante

Tableau des résultats pour analyte 60 -- suite de la page précédente			
**Classement (Qualité)**	**Profil de référence**	**Score Valeur**	**Identifiant NCBI**
10<br>(-)	Bacillus pumilus 10403987	1.54	133055080

Analyte 61

**Nom de l’échantillon:**	F1
**Description de l’échantillon:**	
**ID de l’échantillon:**	F1
**Date/Heure de création de l’échantillon:**	2019-07-10T17:04:20.683
**Type de l’échantillon:**	Échantillon standard
**Méthode de classification :**	MALDI Biotyper MSP Identification Standard Method 1.1
**Méthode de prétraitement :**	BioTyper Preprocessing Standard Method 1.2
**Méthode ACQ :**	D:\Methods\flexControlMethods\MBT_FC.par
**Horodatage ACQ :**	2019-07-10T17:26:23.967
**Méthode AutoXecute :**	MBT_AutoX_smart
**Bibliothèque de MSP utilisée:**	Culturomics / f8c211c3-71c5-471b-8a7e-7f6abca59bb9 / 2019-06-28T14:15:05.935, Timone / 29617d84-2a1e-4bf6-a13d-569eecb48f06 / 2018-04-19T13:24:29.884, BDAL / contains 7854 MSPs / e7ef41ca-b750-4d47-9a1c-6c26fa454356 / 2019-02-01T09:48:20.358

Classement (Qualité)	Profil de référence	Score Valeur	Identifiant NCBI
1 (-)	Bacillus pumilus CSURP8100	1.49	130148166
2 (-)	Rhizobium radiobacter B167 UFL	1.40	358
3 (-)	Lactobacillus paracasei ssp paracasei DSM 5622T DSM	1.33	47714
4 (-)	Bacillus pumilus 10403985	1.30	133055080
5 (-)	Candida parapsilosis CBS 2196 CBS	1.30	5480
6 (-)	Bacillus pumilus 10403987	1.29	133055080
7 (-)	Streptomyces avidinii B190 UFL	1.27	1895
8 (-)	Bacillus cibi DSM 16189T DSM	1.27	265729
9 (-)	Arthrobacter crystallopoietes DSM 20117T DSM	1.26	37928

Tableau des résultats pour analyte 61--suite page suivante

Tableau des résultats pour analyte 61 -- suite de la page précédente			
**Classement (Qualité)**	**Profil de référence**	**Score Valeur**	**Identifiant NCBI**
10<br>(-)	Actinomyces grossensis CSURP242	1.24	133055080

Analyte 62

**Nom de l'échantillon:**	F2
**Description de l'échantillon:**	
**ID de l'échantillon:**	F2
**Date/Heure de création de l'échantillon:**	2019-07-10T17:04:20.685
**Type de l'échantillon:**	Échantillon standard
**Méthode de classification :**	MALDI Biotyper MSP Identification Standard Method 1.1
**Méthode de prétraitement :**	BioTyper Preprocessing Standard Method 1.2
**Méthode ACQ :**	D:\Methods\flexControlMethods\MBT_FC.par
**Horodatage ACQ :**	2019-07-10T17:26:45.564
**Méthode AutoXecute :**	MBT_AutoX_smart
**Bibliothèque de MSP utilisée:**	Culturomics / f8c211c3-71c5-471b-8a7e-7f6abca59bb9 / 2019-06-28T14:15:05.935, Timone / 29617d84-2a1e-4bf6-a13d-569eecb48f06 / 2018-04-19T13:24:29.884, BDAL / contains 7854 MSPs / e7ef41ca-b750-4d47-9a1c-6c26fa454356 / 2019-02-01T09:48:20.358

Classement (Qualité)	Profil de référence	Score Valeur	Identifiant NCBI
1 (+)	Bacillus pumilus 10403329	1.81	133055080
2 (-)	Bacillus pumilus 10403206	1.54	133055080
3 (-)	Bacillus pumilus 10403987	1.51	133055080
4 (-)	Bacillus pumilus CSURP4085	1.50	130148166
5 (-)	Bacillus pumilus CSURP6343	1.47	130148166
6 (-)	Bacillus pumilus CSURP6343	1.47	130148166
7 (-)	Bacillus pumilus CSURP8100	1.45	130148166
8 (-)	Clostridium novyi 1082_ATCC 17861T BOG	1.42	1542
9 (-)	Bacillus pumilus CSURP4105	1.40	130148166

Tableau des résultats pour analyte 62--suite page suivante

Tableau des résultats pour analyte 62 -- suite de la page précédente			
**Classement (Qualité)**	**Profil de référence**	**Score Valeur**	**Identifiant NCBI**
10<br>(-)	Bacillus pumilus 10403990	1.34	133055080

Analyte 63

**Nom de l'échantillon:**	F3
**Description de l'échantillon:**	
**ID de l'échantillon:**	F3
**Date/Heure de création de l'échantillon:**	2019-07-10T17:04:20.686
**Type de l'échantillon:**	Échantillon standard
**Méthode de classification :**	MALDI Biotyper MSP Identification Standard Method 1.1
**Méthode de prétraitement :**	BioTyper Preprocessing Standard Method 1.2
**Méthode ACQ :**	D:\Methods\flexControlMethods\MBT_FC.par
**Horodatage ACQ :**	2019-07-10T17:27:06.360
**Méthode AutoXecute :**	MBT_AutoX_smart
**Bibliothèque de MSP utilisée:**	Culturomics / f8c211c3-71c5-471b-8a7e-7f6abca59bb9 / 2019-06-28T14:15:05.935, Timone / 29617d84-2a1e-4bf6-a13d-569eecb48f06 / 2018-04-19T13:24:29.884, BDAL / contains 7854 MSPs / e7ef41ca-b750-4d47-9a1c-6c26fa454356 / 2019-02-01T09:48:20.358

Classement (Qualité)	Profil de référence	Score Valeur	Identifiant NCBI
1 (+++)	Bacillus pumilus CSURP8100	2.29	130148166
2 (+++)	Bacillus pumilus CSURP4226	2.10	130148166
3 (+++)	Bacillus pumilus CSURP4085	2.09	130148166
4 (+++)	Bacillus pumilus 10403329	2.02	133055080
5 (+)	Bacillus pumilus CSURP6343	1.92	130148166
6 (+)	Bacillus pumilus CSURP6343	1.92	130148166
7 (+)	Bacillus pumilus 10403206	1.84	133055080
8 (+)	Bacillus pumilus CSURP8210	1.83	130148166
9 (+)	Bacillus pumilus CSURP3862	1.76	130148166

Tableau des résultats pour analyte 63--suite page suivante

Tableau des résultats pour analyte 63 -- suite de la page précédente			
**Classement (Qualité)**	**Profil de référence**	**Score Valeur**	**Identifiant NCBI**
10 (+)	Bacillus pumilus 10403987	1.73	133055080

Analyte 64

**Nom de l'échantillon:**	F4
**Description de l'échantillon:**	
**ID de l'échantillon:**	F4
**Date/Heure de création de l'échantillon:**	2019-07-10T17:04:20.688
**Type de l'échantillon:**	Échantillon standard
**Méthode de classification :**	MALDI Biotyper MSP Identification Standard Method 1.1
**Méthode de prétraitement :**	BioTyper Preprocessing Standard Method 1.2
**Méthode ACQ :**	D:\Methods\flexControlMethods\MBT_FC.par
**Horodatage ACQ :**	2019-07-10T17:27:29.061
**Méthode AutoXecute :**	MBT_AutoX_smart
**Bibliothèque de MSP utilisée:**	Culturomics / f8c211c3-71c5-471b-8a7e-7f6abca59bb9 / 2019-06-28T14:15:05.935, Timone / 29617d84-2a1e-4bf6-a13d-569eecb48f06 / 2018-04-19T13:24:29.884, BDAL / contains 7854 MSPs / e7ef41ca-b750-4d47-9a1c-6c26fa454356 / 2019-02-01T09:48:20.358

Classement (Qualité)	Profil de référence	Score Valeur	Identifiant NCBI
1 (+)	Bacillus pumilus CSURP4085	1.99	130148166
2 (+)	Bacillus pumilus CSURP8100	1.94	130148166
3 (+)	Bacillus pumilus CSURP6343	1.86	130148166
4 (+)	Bacillus pumilus CSURP6343	1.86	130148166
5 (+)	Bacillus pumilus CSURP4105	1.79	130148166
6 (+)	Bacillus pumilus 10403329	1.76	133055080
7 (+)	Bacillus pumilus CSURP4226	1.76	130148166
8 (-)	Bacillus pumilus 10403206	1.63	133055080
9 (-)	Bacillus pumilus CSURP8210	1.50	130148166

Tableau des résultats pour analyte 64--suite page suivante

Tableau des résultats pour analyte 64 -- suite de la page précédente			
**Classement (Qualité)**	**Profil de référence**	**Score Valeur**	**Identifiant NCBI**
10<br>(-)	Bacillus pumilus 10403987	1.46	133055080

Analyte 65

**Nom de l'échantillon:**	F5
**Description de l'échantillon:**	
**ID de l'échantillon:**	F5
**Date/Heure de création de l'échantillon:**	2019-07-10T17:04:20.690
**Type de l'échantillon:**	Échantillon standard
**Méthode de classification :**	MALDI Biotyper MSP Identification Standard Method 1.1
**Méthode de prétraitement :**	BioTyper Preprocessing Standard Method 1.2
**Méthode ACQ :**	D:\Methods\flexControlMethods\MBT_FC.par
**Horodatage ACQ :**	2019-07-10T17:27:49.463
**Méthode AutoXecute :**	MBT_AutoX_smart
**Bibliothèque de MSP utilisée:**	Culturomics / f8c211c3-71c5-471b-8a7e-7f6abca59bb9 / 2019-06-28T14:15:05.935, Timone / 29617d84-2a1e-4bf6-a13d-569eecb48f06 / 2018-04-19T13:24:29.884, BDAL / contains 7854 MSPs / e7ef41ca-b750-4d47-9a1c-6c26fa454356 / 2019-02-01T09:48:20.358

Classement (Qualité)	Profil de référence	Score Valeur	Identifiant NCBI
1 (+++)	Bacillus pumilus CSURP4226	2.13	130148166
2 (+++)	Bacillus pumilus CSURP8100	2.04	130148166
3 (+++)	Bacillus pumilus CSURP6343	2.01	130148166
4 (+++)	Bacillus pumilus CSURP6343	2.01	130148166
5 (+)	Bacillus pumilus 10403987	1.99	133055080
6 (+)	Bacillus pumilus 10403990	1.93	133055080
7 (+)	Bacillus pumilus 10403985	1.91	133055080
8 (+)	Bacillus pumilus CSURP4085	1.91	130148166
9 (+)	Bacillus pumilus 10403607	1.90	133055080

Tableau des résultats pour analyte 65--suite page suivante

Tableau des résultats pour analyte 65 -- suite de la page précédente			
**Classement (Qualité)**	**Profil de référence**	**Score Valeur**	**Identifiant NCBI**
10 (+)	Bacillus pumilus CSURP4105	1.89	130148166

Analyte 66

Nom de l'échantillon: F6
Description de l'échantillon:
ID de l'échantillon: F6
Date/Heure de création de l'échantillon: 2019-07-10T17:04:20.692
Type de l'échantillon: Échantillon standard
Méthode de classification : MALDI Biotyper MSP Identification Standard Method 1.1
Méthode de prétraitement : BioTyper Preprocessing Standard Method 1.2
Méthode ACQ : D:\Methods\flexControlMethods\MBT_FC.par
Horodatage ACQ : 2019-07-10T17:28:10.731
Méthode AutoXecute : MBT_AutoX_smart
Bibliothèque de MSP utilisée: Culturomics / f8c211c3-71c5-471b-8a7e-7f6abca59bb9 / 2019-06-28T14:15:05.935, Timone / 29617d84-2a1e-4bf6-a13d-569eecb48f06 / 2018-04-19T13:24:29.884, BDAL / contains 7854 MSPs / e7ef41ca-b750-4d47-9a1c-6c26fa454356 / 2019-02-01T09:48:20.358

Classement (Qualité)	Profil de référence	Score Valeur	Identifiant NCBI
1 (-)	Streptomyces lavendulae B264 UFL	1.31	1914
2 (-)	Staphylococcus xylosus DSM 6179 DSM	1.31	1288
3 (-)	Lactobacillus suebicus DSM 5008 DSM	1.30	152335
4 (-)	Peptinophilus grossensis CSURP254	1.28	130148166
5 (-)	Bacillus pumilus 10403607	1.28	133055080
6 (-)	Pseudomonas veronii B561 UFL	1.28	76761
7 (-)	Lactobacillus paralimentarius DSM 13961 DSM	1.27	83526
8 (-)	Staphylococcus saprophyticus ssp saprophyticus DSM 20038 DSM	1.26	147452
9 (-)	Aromatoleum alkani HxN1 MPB	1.25	12960

Tableau des résultats pour analyte 66--suite page suivante

Tableau des résultats pour analyte 66 -- suite de la page précédente			
**Classement (Qualité)**	**Profil de référence**	**Score Valeur**	**Identifiant NCBI**
10 (-)	Starkeya novella B351 UFL	1.24	921

Analyte 67

**Nom de l'échantillon:**	F7
**Description de l'échantillon:**	
**ID de l'échantillon:**	F7
**Date/Heure de création de l'échantillon:**	2019-07-10T17:04:20.693
**Type de l'échantillon:**	Échantillon standard
**Méthode de classification :**	MALDI Biotyper MSP Identification Standard Method 1.1
**Méthode de prétraitement :**	BioTyper Preprocessing Standard Method 1.2
**Méthode ACQ :**	D:\Methods\flexControlMethods\MBT_FC.par
**Horodatage ACQ :**	2019-07-10T17:28:31.235
**Méthode AutoXecute :**	MBT_AutoX_smart
**Bibliothèque de MSP utilisée:**	Culturomics / f8c211c3-71c5-471b-8a7e-7f6abca59bb9 / 2019-06-28T14:15:05.935, Timone / 29617d84-2a1e-4bf6-a13d-569eecb48f06 / 2018-04-19T13:24:29.884, BDAL / contains 7854 MSPs / e7ef41ca-b750-4d47-9a1c-6c26fa454356 / 2019-02-01T09:48:20.358

Classement (Qualité)	Profil de référence	Score Valeur	Identifiant NCBI
1 (+++)	Bacillus pumilus CSURP8100	2.28	130148166
2 (+++)	Bacillus pumilus CSURP6343	2.15	130148166
3 (+++)	Bacillus pumilus CSURP6343	2.15	130148166
4 (+++)	Bacillus pumilus CSURP4085	2.06	130148166
5 (+++)	Bacillus pumilus CSURP4105	2.05	130148166
6 (+++)	Bacillus pumilus CSURP4226	2.01	130148166
7 (+)	Bacillus pumilus 10403607	1.92	133055080
8 (+)	Bacillus pumilus 10403329	1.91	133055080
9 (+)	Bacillus pumilus 10403206	1.88	133055080

Tableau des résultats pour analyte 67--suite page suivante

Tableau des résultats pour analyte 67 -- suite de la page précédente			
**Classement (Qualité)**	**Profil de référence**	**Score Valeur**	**Identifiant NCBI**
10<br>(+)	Bacillus pumilus 10149151	1.84	133055080

Analyte 68

**Nom de l'échantillon:**	F8
**Description de l'échantillon:**	
**ID de l'échantillon:**	F8
**Date/Heure de création de l'échantillon:**	2019-07-10T17:04:20.695
**Type de l'échantillon:**	Échantillon standard
**Méthode de classification :**	MALDI Biotyper MSP Identification Standard Method 1.1
**Méthode de prétraitement :**	BioTyper Preprocessing Standard Method 1.2
**Méthode ACQ :**	D:\Methods\flexControlMethods\MBT_FC.par
**Horodatage ACQ :**	2019-07-10T17:28:52.423
**Méthode AutoXecute :**	MBT_AutoX_smart
**Bibliothèque de MSP utilisée:**	Culturomics / f8c211c3-71c5-471b-8a7e-7f6abca59bb9 / 2019-06-28T14:15:05.935, Timone / 29617d84-2a1e-4bf6-a13d-569eecb48f06 / 2018-04-19T13:24:29.884, BDAL / contains 7854 MSPs / e7ef41ca-b750-4d47-9a1c-6c26fa454356 / 2019-02-01T09:48:20.358

Classement (Qualité)	Profil de référence	Score Valeur	Identifiant NCBI
1 (+)	Bacillus simplex CSURP558	1.80	130148166
2 (-)	Bacillus simplex 110818	1.67	133055080
3 (-)	Bacillus simplex 10109635	1.57	133055080
4 (-)	Bacillus muralis DSM 16288T DSM	1.47	264697
5 (-)	Clostridium spiroforme 1047_NCTC 11211T BOG	1.44	29348
6 (-)	Bacillus megaterium DSM 32T DSM	1.43	1404
7 (-)	Bacillus psychrosaccharolyticus DSM 6T DSM	1.42	1407
8 (-)	Bacillus simplex DSM 1321T DSM	1.42	1478
9 (-)	Bacillus endophyticus DSM 13796T DSM	1.41	135735

Tableau des résultats pour analyte 68--suite page suivante

Tableau des résultats pour analyte 68 -- suite de la page précédente			
**Classement (Qualité)**	**Profil de référence**	**Score Valeur**	**Identifiant NCBI**
10<br>(-)	Bacillus simplex CSURP5196	1.36	130148166

Analyte 69

**Nom de l'échantillon:**	F9
**Description de l'échantillon:**	
**ID de l'échantillon:**	F9
**Date/Heure de création de l'échantillon:**	2019-07-10T17:04:20.697
**Type de l'échantillon:**	Échantillon standard
**Méthode de classification :**	MALDI Biotyper MSP Identification Standard Method 1.1
**Méthode de prétraitement :**	BioTyper Preprocessing Standard Method 1.2
**Méthode ACQ :**	D:\Methods\flexControlMethods\MBT_FC.par
**Horodatage ACQ :**	2019-07-10T17:29:12.863
**Méthode AutoXecute :**	MBT_AutoX_smart
**Bibliothèque de MSP utilisée:**	Culturomics / f8c211c3-71c5-471b-8a7e-7f6abca59bb9 / 2019-06-28T14:15:05.935, Timone / 29617d84-2a1e-4bf6-a13d-569eecb48f06 / 2018-04-19T13:24:29.884, BDAL / contains 7854 MSPs / e7ef41ca-b750-4d47-9a1c-6c26fa454356 / 2019-02-01T09:48:20.358

Classement (Qualité)	Profil de référence	Score Valeur	Identifiant NCBI
1 (+++)	Bacillus pumilus CSURP4226	2.37	130148166
2 (+++)	Bacillus pumilus CSURP8100	2.21	130148166
3 (+++)	Bacillus pumilus CSURP4085	2.07	130148166
4 (+++)	Bacillus pumilus 10403329	2.05	133055080
5 (+++)	Bacillus pumilus CSURP4105	2.03	130148166
6 (+++)	Bacillus pumilus 10403987	2.03	133055080
7 (+++)	Bacillus pumilus 10403607	2.02	133055080
8 (+++)	Bacillus pumilus 10403990	2.01	133055080
9 (+)	Bacillus pumilus 10403985	1.94	133055080

Tableau des résultats pour analyte 69--suite page suivante

Tableau des résultats pour analyte 69 -- suite de la page précédente			
**Classement (Qualité)**	**Profil de référence**	**Score Valeur**	**Identifiant NCBI**
10 (+)	Bacillus pumilus 10403206	1.91	133055080

Analyte 70

**Nom de l'échantillon:**	F10
**Description de l'échantillon:**	
**ID de l'échantillon:**	F10
**Date/Heure de création de l'échantillon:**	2019-07-10T17:04:20.699
**Type de l'échantillon:**	Échantillon standard
**Méthode de classification :**	MALDI Biotyper MSP Identification Standard Method 1.1
**Méthode de prétraitement :**	BioTyper Preprocessing Standard Method 1.2
**Méthode ACQ :**	D:\Methods\flexControlMethods\MBT_FC.par
**Horodatage ACQ :**	2019-07-10T17:29:35.022
**Méthode AutoXecute :**	MBT_AutoX_smart
**Bibliothèque de MSP utilisée:**	Culturomics / f8c211c3-71c5-471b-8a7e-7f6abca59bb9 / 2019-06-28T14:15:05.935, Timone / 29617d84-2a1e-4bf6-a13d-569eecb48f06 / 2018-04-19T13:24:29.884, BDAL / contains 7854 MSPs / e7ef41ca-b750-4d47-9a1c-6c26fa454356 / 2019-02-01T09:48:20.358

Classement (Qualité)	Profil de référence	Score Valeur	Identifiant NCBI
1 (+++)	Bacillus pumilus CSURP4226	2.11	130148166
2 (+++)	Bacillus pumilus CSURP8100	2.06	130148166
3 (+)	Bacillus pumilus CSURP4105	1.96	130148166
4 (+)	Bacillus pumilus 10403329	1.94	133055080
5 (+)	Bacillus pumilus 10403987	1.81	133055080
6 (+)	Bacillus pumilus CSURP6343	1.79	130148166
7 (+)	Bacillus pumilus CSURP6343	1.79	130148166
8 (+)	Bacillus pumilus CSURP4085	1.74	130148166
9 (-)	Bacillus pumilus CSURP3862	1.67	130148166

Tableau des résultats pour analyte 70--suite page suivante

Tableau des résultats pour analyte 70 -- suite de la page précédente			
**Classement (Qualité)**	**Profil de référence**	**Score Valeur**	**Identifiant NCBI**
10<br>(-)	Bacillus pumilus 10403751	1.64	133055080

Analyte 71

**Nom de l'échantillon:**	F11
**Description de l'échantillon:**	
**ID de l'échantillon:**	F11
**Date/Heure de création de l'échantillon:**	2019-07-10T17:04:20.701
**Type de l'échantillon:**	Échantillon standard
**Méthode de classification :**	MALDI Biotyper MSP Identification Standard Method 1.1
**Méthode de prétraitement :**	BioTyper Preprocessing Standard Method 1.2
**Méthode ACQ :**	D:\Methods\flexControlMethods\MBT_FC.par
**Horodatage ACQ :**	2019-07-10T17:29:56.490
**Méthode AutoXecute :**	MBT_AutoX_smart
**Bibliothèque de MSP utilisée:**	Culturomics / f8c211c3-71c5-471b-8a7e-7f6abca59bb9 / 2019-06-28T14:15:05.935, Timone / 29617d84-2a1e-4bf6-a13d-569eecb48f06 / 2018-04-19T13:24:29.884, BDAL / contains 7854 MSPs / e7ef41ca-b750-4d47-9a1c-6c26fa454356 / 2019-02-01T09:48:20.358

Classement (Qualité)	Profil de référence	Score Valeur	Identifiant NCBI
1 (+)	Bacillus pumilus CSURP4085	1.94	130148166
2 (+)	Bacillus pumilus CSURP8100	1.92	130148166
3 (+)	Bacillus pumilus 10403329	1.81	133055080
4 (+)	Bacillus pumilus 10403987	1.77	133055080
5 (+)	Bacillus pumilus CSURP4226	1.76	130148166
6 (+)	Bacillus pumilus CSURP4105	1.76	130148166
7 (+)	Bacillus pumilus CSURP6343	1.74	130148166
8 (+)	Bacillus pumilus CSURP6343	1.74	130148166
9 (-)	Bacillus pumilus 10403206	1.61	133055080

Tableau des résultats pour analyte 71--suite page suivante

Tableau des résultats pour analyte 71 -- suite de la page précédente			
**Classement (Qualité)**	**Profil de référence**	**Score Valeur**	**Identifiant NCBI**
10<br>(-)	Bacillus pumilus 10403990	1.47	133055080

Analyte 72

**Nom de l'échantillon:**	F12
**Description de l'échantillon:**	
**ID de l'échantillon:**	F12
**Date/Heure de création de l'échantillon:**	2019-07-10T17:04:20.703
**Type de l'échantillon:**	Échantillon standard
**Méthode de classification :**	MALDI Biotyper MSP Identification Standard Method 1.1
**Méthode de prétraitement :**	BioTyper Preprocessing Standard Method 1.2
**Méthode ACQ :**	D:\Methods\flexControlMethods\MBT_FC.par
**Horodatage ACQ :**	2019-07-10T17:30:17.307
**Méthode AutoXecute :**	MBT_AutoX_smart
**Bibliothèque de MSP utilisée:**	Culturomics / f8c211c3-71c5-471b-8a7e-7f6abca59bb9 / 2019-06-28T14:15:05.935, Timone / 29617d84-2a1e-4bf6-a13d-569eecb48f06 / 2018-04-19T13:24:29.884, BDAL / contains 7854 MSPs / e7ef41ca-b750-4d47-9a1c-6c26fa454356 / 2019-02-01T09:48:20.358

Classement (Qualité)	Profil de référence	Score Valeur	Identifiant NCBI
1 (+++)	Bacillus pumilus CSURP8100	2.16	130148166
2 (+++)	Bacillus pumilus CSURP4085	2.14	130148166
3 (+++)	Bacillus pumilus CSURP4226	2.01	130148166
4 (+)	Bacillus pumilus 10403329	1.96	133055080
5 (+)	Bacillus pumilus CSURP6343	1.94	130148166
6 (+)	Bacillus pumilus CSURP6343	1.94	130148166
7 (+)	Bacillus pumilus CSURP4105	1.93	130148166
8 (+)	Bacillus pumilus 10403987	1.74	133055080
9 (+)	Bacillus pumilus 10403206	1.74	133055080

Tableau des résultats pour analyte 72--suite page suivante

Tableau des résultats pour analyte 72 -- suite de la page précédente			
**Classement (Qualité)**	**Profil de référence**	**Score Valeur**	**Identifiant NCBI**
10<br>(-)	Bacillus pumilus 10403751	1.65	133055080

Analyte 73

**Nom de l'échantillon:**	G1
**Description de l'échantillon:**	
**ID de l'échantillon:**	G1
**Date/Heure de création de l'échantillon:**	2019-07-10T17:04:20.705
**Type de l'échantillon:**	Échantillon standard
**Méthode de classification :**	MALDI Biotyper MSP Identification Standard Method 1.1
**Méthode de prétraitement :**	BioTyper Preprocessing Standard Method 1.2
**Méthode ACQ :**	D:\Methods\flexControlMethods\MBT_FC.par
**Horodatage ACQ :**	2019-07-10T17:30:38.652
**Méthode AutoXecute :**	MBT_AutoX_smart
**Bibliothèque de MSP utilisée:**	Culturomics / f8c211c3-71c5-471b-8a7e-7f6abca59bb9 / 2019-06-28T14:15:05.935, Timone / 29617d84-2a1e-4bf6-a13d-569eecb48f06 / 2018-04-19T13:24:29.884, BDAL / contains 7854 MSPs / e7ef41ca-b750-4d47-9a1c-6c26fa454356 / 2019-02-01T09:48:20.358

Classement (Qualité)	Profil de référence	Score Valeur	Identifiant NCBI
1 (+++)	Bacillus pumilus CSURP4226	2.45	130148166
2 (+++)	Bacillus pumilus CSURP8100	2.32	130148166
3 (+++)	Bacillus pumilus CSURP4085	2.23	130148166
4 (+++)	Bacillus pumilus CSURP6343	2.05	130148166
5 (+++)	Bacillus pumilus CSURP6343	2.05	130148166
6 (+++)	Bacillus pumilus CSURP4105	2.04	130148166
7 (+)	Bacillus pumilus 10403607	1.98	133055080
8 (+)	Bacillus pumilus 10403985	1.97	133055080
9 (+)	Bacillus pumilus 10403329	1.95	133055080

Tableau des résultats pour analyte 73--suite page suivante

Tableau des résultats pour analyte 73 -- suite de la page précédente			
**Classement (Qualité)**	**Profil de référence**	**Score Valeur**	**Identifiant NCBI**
10 (+)	Bacillus pumilus 10403987	1.93	133055080

Analyte 74

**Nom de l'échantillon:**	G2
**Description de l'échantillon:**	
**ID de l'échantillon:**	G2
**Date/Heure de création de l'échantillon:**	2019-07-10T17:04:20.708
**Type de l'échantillon:**	Échantillon standard
**Méthode de classification :**	MALDI Biotyper MSP Identification Standard Method 1.1
**Méthode de prétraitement :**	BioTyper Preprocessing Standard Method 1.2
**Méthode ACQ :**	D:\Methods\flexControlMethods\MBT_FC.par
**Horodatage ACQ :**	2019-07-10T17:31:00.202
**Méthode AutoXecute :**	MBT_AutoX_smart
**Bibliothèque de MSP utilisée:**	Culturomics / f8c211c3-71c5-471b-8a7e-7f6abca59bb9 / 2019-06-28T14:15:05.935, Timone / 29617d84-2a1e-4bf6-a13d-569eecb48f06 / 2018-04-19T13:24:29.884, BDAL / contains 7854 MSPs / e7ef41ca-b750-4d47-9a1c-6c26fa454356 / 2019-02-01T09:48:20.358

Classement (Qualité)	Profil de référence	Score Valeur	Identifiant NCBI
1 (+++)	Bacillus pumilus CSURP4226	2.23	130148166
2 (+++)	Bacillus pumilus CSURP8100	2.11	130148166
3 (+++)	Bacillus pumilus CSURP4085	2.07	130148166
4 (+)	Bacillus pumilus 10403987	1.96	133055080
5 (+)	Bacillus pumilus CSURP4105	1.87	130148166
6 (+)	Bacillus pumilus 10403329	1.83	133055080
7 (+)	Bacillus pumilus 10403990	1.78	133055080
8 (+)	Bacillus pumilus 10403985	1.76	133055080
9 (+)	Bacillus pumilus CSURP505	1.70	130148166

Tableau des résultats pour analyte 74--suite page suivante

Tableau des résultats pour analyte 74 -- suite de la page précédente			
**Classement (Qualité)**	**Profil de référence**	**Score Valeur**	**Identifiant NCBI**
10 (-)	Bacillus pumilus CSURP3862	1.69	130148166

Analyte 75

**Nom de l'échantillon:**	G3
**Description de l'échantillon:**	
**ID de l'échantillon:**	G3
**Date/Heure de création de l'échantillon:**	2019-07-10T17:04:20.710
**Type de l'échantillon:**	Échantillon standard
**Méthode de classification :**	MALDI Biotyper MSP Identification Standard Method 1.1
**Méthode de prétraitement :**	BioTyper Preprocessing Standard Method 1.2
**Méthode ACQ :**	D:\Methods\flexControlMethods\MBT_FC.par
**Horodatage ACQ :**	2019-07-10T17:31:21.428
**Méthode AutoXecute :**	MBT_AutoX_smart
**Bibliothèque de MSP utilisée:**	Culturomics / f8c211c3-71c5-471b-8a7e-7f6abca59bb9 / 2019-06-28T14:15:05.935, Timone / 29617d84-2a1e-4bf6-a13d-569eecb48f06 / 2018-04-19T13:24:29.884, BDAL / contains 7854 MSPs / e7ef41ca-b750-4d47-9a1c-6c26fa454356 / 2019-02-01T09:48:20.358

Classement (Qualité)	Profil de référence	Score Valeur	Identifiant NCBI
1 (+++)	Bacillus pumilus CSURP8100	2.32	130148166
2 (+++)	Bacillus pumilus CSURP4226	2.28	130148166
3 (+++)	Bacillus pumilus 10403329	2.11	133055080
4 (+++)	Bacillus pumilus 10403607	2.07	133055080
5 (+++)	Bacillus pumilus CSURP4105	2.03	130148166
6 (+++)	Bacillus pumilus 10403987	2.00	133055080
7 (+)	Bacillus pumilus 10403985	1.98	133055080
8 (+)	Bacillus pumilus CSURP4085	1.97	130148166
9 (+)	Bacillus pumilus 10403990	1.92	133055080

Tableau des résultats pour analyte 75--suite page suivante

Tableau des résultats pour analyte 75 -- suite de la page précédente			
**Classement (Qualité)**	**Profil de référence**	**Score Valeur**	**Identifiant NCBI**
10 (+)	Bacillus pumilus 10403751	1.91	133055080

Analyte 76

Nom de l'échantillon: G4
Description de l'échantillon:
ID de l'échantillon: G4
Date/Heure de création de l'échantillon: 2019-07-10T17:04:20.712
Type de l'échantillon: Échantillon standard
Méthode de classification : MALDI Biotyper MSP Identification Standard Method 1.1
Méthode de prétraitement : BioTyper Preprocessing Standard Method 1.2
Méthode ACQ : D:\Methods\flexControlMethods\MBT_FC.par
Horodatage ACQ : 2019-07-10T17:31:42.819
Méthode AutoXecute : MBT_AutoX_smart
Bibliothèque de MSP utilisée: Culturomics / f8c211c3-71c5-471b-8a7e-7f6abca59bb9 / 2019-06-28T14:15:05.935, Timone / 29617d84-2a1e-4bf6-a13d-569eecb48f06 / 2018-04-19T13:24:29.884, BDAL / contains 7854 MSPs / e7ef41ca-b750-4d47-9a1c-6c26fa454356 / 2019-02-01T09:48:20.358

Classement (Qualité)	Profil de référence	Score Valeur	Identifiant NCBI
1 (+++)	Bacillus pumilus CSURP8100	2.26	130148166
2 (+++)	Bacillus pumilus CSURP4226	2.23	130148166
3 (+++)	Bacillus pumilus CSURP6343	2.22	130148166
4 (+++)	Bacillus pumilus CSURP6343	2.22	130148166
5 (+++)	Bacillus pumilus CSURP4105	2.05	130148166
6 (+++)	Bacillus pumilus 10403329	2.01	133055080
7 (+++)	Bacillus pumilus 10403607	2.01	133055080
8 (+++)	Bacillus pumilus 10149151	2.01	133055080
9 (+)	Bacillus pumilus 10403985	1.99	133055080

Tableau des résultats pour analyte 76--suite page suivante

Tableau des résultats pour analyte 76 -- suite de la page précédente			
**Classement (Qualité)**	**Profil de référence**	**Score Valeur**	**Identifiant NCBI**
10 (+)	Bacillus pumilus 10403987	1.93	133055080

Analyte 77

**Nom de l'échantillon:**	G5
**Description de l'échantillon:**	
**ID de l'échantillon:**	G5
**Date/Heure de création de l'échantillon:**	2019-07-10T17:04:20.714
**Type de l'échantillon:**	Échantillon standard
**Méthode de classification :**	MALDI Biotyper MSP Identification Standard Method 1.1
**Méthode de prétraitement :**	BioTyper Preprocessing Standard Method 1.2
**Méthode ACQ :**	D:\Methods\flexControlMethods\MBT_FC.par
**Horodatage ACQ :**	2019-07-10T17:32:03.890
**Méthode AutoXecute :**	MBT_AutoX_smart
**Bibliothèque de MSP utilisée:**	Culturomics / f8c211c3-71c5-471b-8a7e-7f6abca59bb9 / 2019-06-28T14:15:05.935, Timone / 29617d84-2a1e-4bf6-a13d-569eecb48f06 / 2018-04-19T13:24:29.884, BDAL / contains 7854 MSPs / e7ef41ca-b750-4d47-9a1c-6c26fa454356 / 2019-02-01T09:48:20.358

Classement (Qualité)	Profil de référence	Score Valeur	Identifiant NCBI
1 (-)	Bacteroides vulgatus DSM 1447T DSM	1.49	821
2 (-)	Bacillus subtilis 10402717	1.43	133055080
3 (-)	Staphylococcus aureus ATCC 33591 THL	1.43	1280
4 (-)	Propionimicrobium lymphophilum CSURP108	1.41	130148166
5 (-)	Arthrobacter pyridinolis B384 UFL	1.38	1663
6 (-)	Lactobacillus paracasei ssp paracasei DSM 20207 DSM	1.38	47714
7 (-)	Africanibacillus timonensis P6386P	1.34	130148166
8 (-)	Bacillus pseudomycoides DSM 12442T DSM	1.33	64104
9 (-)	Lactobacillus plantarum DSM 20205 DSM	1.33	1590

Tableau des résultats pour analyte 77--suite page suivante

Tableau des résultats pour analyte 77 -- suite de la page précédente			
**Classement (Qualité)**	**Profil de référence**	**Score Valeur**	**Identifiant NCBI**
10<br>(-)	Pseudomonas nitroreducens LMG 20221T HAM	1.33	46680

Analyte 78

**Nom de l'échantillon:**	G6
**Description de l'échantillon:**	
**ID de l'échantillon:**	G6
**Date/Heure de création de l'échantillon:**	2019-07-10T17:04:20.717
**Type de l'échantillon:**	Échantillon standard
**Méthode de classification :**	MALDI Biotyper MSP Identification Standard Method 1.1
**Méthode de prétraitement :**	BioTyper Preprocessing Standard Method 1.2
**Méthode ACQ :**	D:\Methods\flexControlMethods\MBT_FC.par
**Horodatage ACQ :**	2019-07-10T17:32:23.705
**Méthode AutoXecute :**	MBT_AutoX_smart
**Bibliothèque de MSP utilisée:**	Culturomics / f8c211c3-71c5-471b-8a7e-7f6abca59bb9 / 2019-06-28T14:15:05.935, Timone / 29617d84-2a1e-4bf6-a13d-569eecb48f06 / 2018-04-19T13:24:29.884, BDAL / contains 7854 MSPs / e7ef41ca-b750-4d47-9a1c-6c26fa454356 / 2019-02-01T09:48:20.358

Classement (Qualité)	Profil de référence	Score Valeur	Identifiant NCBI
1 (-)	Bacillus valismortis CSURP2348	1.67	130148166
2 (-)	Bacillus amyloliquefaciens CSURP714	1.49	130148166
3 (-)	Brevibacterium casei IMET 10997T HKJ	1.39	33889
4 (-)	Filifactor villosus 1051_NCTC 11220T BOG	1.37	29374
5 (-)	Bacillus subtilis ssp subtilis DSM 10T DSM	1.35	135461
6 (-)	Staphylococcus cohnii ssp cohnii DSM 20260T DSM	1.30	74704
7 (-)	Bacillus pumilus 10149151	1.29	133055080
8 (-)	Staphylococcus cohnii ssp urealyticus DSM 6718T DSM	1.28	94138
9 (-)	Listeria grayi DSM 20596 DSM	1.27	1641

Tableau des résultats pour analyte 78--suite page suivante

Tableau des résultats pour analyte 78 -- suite de la page précédente			
**Classement (Qualité)**	**Profil de référence**	**Score Valeur**	**Identifiant NCBI**
10<br>(-)	Lactobacillus paracasei ssp paracasei DSM 20312 DSM	1.26	47714

Analyte 79

**Nom de l'échantillon:**	G7
**Description de l'échantillon:**	
**ID de l'échantillon:**	G7
**Date/Heure de création de l'échantillon:**	2019-07-10T17:04:20.720
**Type de l'échantillon:**	Échantillon standard
**Méthode de classification :**	MALDI Biotyper MSP Identification Standard Method 1.1
**Méthode de prétraitement :**	BioTyper Preprocessing Standard Method 1.2
**Méthode ACQ :**	D:\Methods\flexControlMethods\MBT_FC.par
**Horodatage ACQ :**	2019-07-10T17:32:43.928
**Méthode AutoXecute :**	MBT_AutoX_smart
**Bibliothèque de MSP utilisée:**	Culturomics / f8c211c3-71c5-471b-8a7e-7f6abca59bb9 / 2019-06-28T14:15:05.935, Timone / 29617d84-2a1e-4bf6-a13d-569eecb48f06 / 2018-04-19T13:24:29.884, BDAL / contains 7854 MSPs / e7ef41ca-b750-4d47-9a1c-6c26fa454356 / 2019-02-01T09:48:20.358

Classement (Qualité)	Profil de référence	Score Valeur	Identifiant NCBI
1 (+++)	Bacillus pumilus CSURP8100	2.14	130148166
2 (+++)	Bacillus pumilus CSURP4105	2.05	130148166
3 (+++)	Bacillus pumilus 10403329	2.03	133055080
4 (+++)	Bacillus pumilus 10403985	2.02	133055080
5 (+++)	Bacillus pumilus 10403607	2.02	133055080
6 (+)	Bacillus pumilus 10149151	1.98	133055080
7 (+)	Bacillus pumilus CSURP6343	1.97	130148166
8 (+)	Bacillus pumilus CSURP6343	1.97	130148166
9 (+)	Bacillus pumilus 10403987	1.94	133055080

Tableau des résultats pour analyte 79--suite page suivante

Tableau des résultats pour analyte 79 -- suite de la page précédente			
**Classement (Qualité)**	**Profil de référence**	**Score Valeur**	**Identifiant NCBI**
10<br>(+)	Bacillus pumilus 10403751	1.94	133055080

Analyte 80

**Nom de l'échantillon:**	G8
**Description de l'échantillon:**	
**ID de l'échantillon:**	G8
**Date/Heure de création de l'échantillon:**	2019-07-10T17:04:20.722
**Type de l'échantillon:**	Échantillon standard
**Méthode de classification :**	MALDI Biotyper MSP Identification Standard Method 1.1
**Méthode de prétraitement :**	BioTyper Preprocessing Standard Method 1.2
**Méthode ACQ :**	D:\Methods\flexControlMethods\MBT_FC.par
**Horodatage ACQ :**	2019-07-10T17:33:04.912
**Méthode AutoXecute :**	MBT_AutoX_smart
**Bibliothèque de MSP utilisée:**	Culturomics / f8c211c3-71c5-471b-8a7e-7f6abca59bb9 / 2019-06-28T14:15:05.935, Timone / 29617d84-2a1e-4bf6-a13d-569eecb48f06 / 2018-04-19T13:24:29.884, BDAL / contains 7854 MSPs / e7ef41ca-b750-4d47-9a1c-6c26fa454356 / 2019-02-01T09:48:20.358

Classement (Qualité)	Profil de référence	Score Valeur	Identifiant NCBI
1 (+)	Bacillus pumilus CSURP4085	1.90	130148166
2 (+)	Bacillus pumilus CSURP6343	1.89	130148166
3 (+)	Bacillus pumilus CSURP6343	1.89	130148166
4 (+)	Bacillus pumilus CSURP8100	1.85	130148166
5 (+)	Bacillus pumilus 10403329	1.78	133055080
6 (-)	Bacillus pumilus 10403206	1.68	133055080
7 (-)	Bacillus pumilus CSURP4226	1.68	130148166
8 (-)	Bacillus pumilus CSURP4105	1.66	130148166
9 (-)	Bacillus pumilus 10149151	1.64	133055080

Tableau des résultats pour analyte 80--suite page suivante

Tableau des résultats pour analyte 80 -- suite de la page précédente			
**Classement (Qualité)**	**Profil de référence**	**Score Valeur**	**Identifiant NCBI**
10<br>(-)	Bacillus pumilus 10403607	1.63	133055080

Analyte 81

**Nom de l'échantillon:**	G9
**Description de l'échantillon:**	
**ID de l'échantillon:**	G9
**Date/Heure de création de l'échantillon:**	2019-07-10T17:04:20.725
**Type de l'échantillon:**	Échantillon standard
**Méthode de classification :**	MALDI Biotyper MSP Identification Standard Method 1.1
**Méthode de prétraitement :**	BioTyper Preprocessing Standard Method 1.2
**Méthode ACQ :**	D:\Methods\flexControlMethods\MBT_FC.par
**Horodatage ACQ :**	2019-07-10T17:33:25.691
**Méthode AutoXecute :**	MBT_AutoX_smart
**Bibliothèque de MSP utilisée:**	Culturomics / f8c211c3-71c5-471b-8a7e-7f6abca59bb9 / 2019-06-28T14:15:05.935, Timone / 29617d84-2a1e-4bf6-a13d-569eecb48f06 / 2018-04-19T13:24:29.884, BDAL / contains 7854 MSPs / e7ef41ca-b750-4d47-9a1c-6c26fa454356 / 2019-02-01T09:48:20.358

Classement (Qualité)	Profil de référence	Score Valeur	Identifiant NCBI
1 (+++)	Bacillus pumilus CSURP4226	2.21	130148166
2 (+++)	Bacillus pumilus CSURP8100	2.05	130148166
3 (+)	Bacillus pumilus CSURP4085	1.95	130148166
4 (+)	Bacillus pumilus CSURP6343	1.94	130148166
5 (+)	Bacillus pumilus CSURP6343	1.94	130148166
6 (+)	Bacillus pumilus 10403985	1.90	133055080
7 (+)	Bacillus pumilus CSURP4105	1.89	130148166
8 (+)	Bacillus pumilus 10403987	1.87	133055080
9 (+)	Bacillus pumilus 10403607	1.87	133055080

Tableau des résultats pour analyte 81--suite page suivante

Tableau des résultats pour analyte 81 -- suite de la page précédente			
**Classement (Qualité)**	**Profil de référence**	**Score Valeur**	**Identifiant NCBI**
10<br>(+)	Bacillus pumilus 10403329	1.87	133055080

Analyte 82

**Nom de l'échantillon:**	G10
**Description de l'échantillon:**	
**ID de l'échantillon:**	G10
**Date/Heure de création de l'échantillon:**	2019-07-10T17:04:20.727
**Type de l'échantillon:**	Échantillon standard
**Méthode de classification :**	MALDI Biotyper MSP Identification Standard Method 1.1
**Méthode de prétraitement :**	BioTyper Preprocessing Standard Method 1.2
**Méthode ACQ :**	D:\Methods\flexControlMethods\MBT_FC.par
**Horodatage ACQ :**	2019-07-10T17:33:44.409
**Méthode AutoXecute :**	MBT_AutoX_smart
**Bibliothèque de MSP utilisée:**	Culturomics / f8c211c3-71c5-471b-8a7e-7f6abca59bb9 / 2019-06-28T14:15:05.935, Timone / 29617d84-2a1e-4bf6-a13d-569eecb48f06 / 2018-04-19T13:24:29.884, BDAL / contains 7854 MSPs / e7ef41ca-b750-4d47-9a1c-6c26fa454356 / 2019-02-01T09:48:20.358

Classement (Qualité)	Profil de référence	Score Valeur	Identifiant NCBI
1 (+++)	Bacillus pumilus CSURP4226	2.42	130148166
2 (+++)	Bacillus pumilus CSURP8100	2.40	130148166
3 (+++)	Bacillus pumilus CSURP4085	2.19	130148166
4 (+++)	Bacillus pumilus CSURP6343	2.19	130148166
5 (+++)	Bacillus pumilus CSURP6343	2.19	130148166
6 (+++)	Bacillus pumilus 10403607	2.10	133055080
7 (+++)	Bacillus pumilus 10403329	2.10	133055080
8 (+++)	Bacillus pumilus CSURP4105	2.08	130148166
9 (+++)	Bacillus pumilus 10403987	2.06	133055080

Tableau des résultats pour analyte 82--suite page suivante

Tableau des résultats pour analyte 82 -- suite de la page précédente			
**Classement (Qualité)**	**Profil de référence**	**Score Valeur**	**Identifiant NCBI**
10 (+++)	Bacillus pumilus 10403985	2.03	133055080

Analyte 83

**Nom de l'échantillon:**	G11
**Description de l'échantillon:**	
**ID de l'échantillon:**	G11
**Date/Heure de création de l'échantillon:**	2019-07-10T17:04:20.730
**Type de l'échantillon:**	Échantillon standard
**Méthode de classification :**	MALDI Biotyper MSP Identification Standard Method 1.1
**Méthode de prétraitement :**	BioTyper Preprocessing Standard Method 1.2
**Méthode ACQ :**	D:\Methods\flexControlMethods\MBT_FC.par
**Horodatage ACQ :**	2019-07-10T17:34:05.567
**Méthode AutoXecute :**	MBT_AutoX_smart
**Bibliothèque de MSP utilisée:**	Culturomics / f8c211c3-71c5-471b-8a7e-7f6abca59bb9 / 2019-06-28T14:15:05.935, Timone / 29617d84-2a1e-4bf6-a13d-569eecb48f06 / 2018-04-19T13:24:29.884, BDAL / contains 7854 MSPs / e7ef41ca-b750-4d47-9a1c-6c26fa454356 / 2019-02-01T09:48:20.358

Classement (Qualité)	Profil de référence	Score Valeur	Identifiant NCBI
1 (-)	Aromatoleum aromaticum EbN1 MPB	1.46	12960
2 (-)	Bacillus pumilus 10149151	1.43	133055080
3 (-)	Bacillus subtilis ssp subtilis DSM 5660 DSM	1.42	135461
4 (-)	Bacillus amyloliquefaciens 10403754	1.39	133055080
5 (-)	Bacillus amyloliquefaciens 10403754	1.39	133055080
6 (-)	Thauera linaloolentis 47Lol MPB	1.39	76112
7 (-)	Staphylococcus aureus ssp aureus DSM 4910 DSM	1.39	46170
8 (-)	Aromatoleum terpenicum pCyN1 MPB	1.37	12960
9 (-)	Staphylococcus epidermidis 6b_s ESL	1.34	1282

Tableau des résultats pour analyte 83--suite page suivante

Tableau des résultats pour analyte 83 -- suite de la page précédente			
**Classement (Qualité)**	**Profil de référence**	**Score Valeur**	**Identifiant NCBI**
10<br>(-)	Terrimonas ferruginea DSM 30193T HAM	1.30	249

Analyte 84

**Nom de l'échantillon:**	G12
**Description de l'échantillon:**	
**ID de l'échantillon:**	G12
**Date/Heure de création de l'échantillon:**	2019-07-10T17:04:20.732
**Type de l'échantillon:**	Échantillon standard
**Méthode de classification :**	MALDI Biotyper MSP Identification Standard Method 1.1
**Méthode de prétraitement :**	BioTyper Preprocessing Standard Method 1.2
**Méthode ACQ :**	D:\Methods\flexControlMethods\MBT_FC.par
**Horodatage ACQ :**	2019-07-10T17:34:25.711
**Méthode AutoXecute :**	MBT_AutoX_smart
**Bibliothèque de MSP utilisée:**	Culturomics / f8c211c3-71c5-471b-8a7e-7f6abca59bb9 / 2019-06-28T14:15:05.935, Timone / 29617d84-2a1e-4bf6-a13d-569eecb48f06 / 2018-04-19T13:24:29.884, BDAL / contains 7854 MSPs / e7ef41ca-b750-4d47-9a1c-6c26fa454356 / 2019-02-01T09:48:20.358

Classement (Qualité)	Profil de référence	Score Valeur	Identifiant NCBI
1 (+++)	Bacillus subtilis CSURP291	2.23	130148166
2 (+++)	Bacillus subtilis strain CD7.3	2.22	133993714
3 (+++)	Bacillus subtilis DSM 5611 DSM	2.02	1423
4 (+++)	Bacillus subtilis CD15-3	2.00	133993714
5 (+)	Bacillus subtilis ssp subtilis DSM 5660 DSM	1.99	135461
6 (+)	Lactobacillus fermentum CSURP4362	1.94	130148166
7 (+)	Bacillus subtilis CSURP3865	1.93	130148166
8 (+)	Bacillus subtilis DSM 5552 DSM	1.84	1423
9 (+)	Bacillus mojavensis DSM 9205T DSM	1.83	72360

Tableau des résultats pour analyte 84--suite page suivante

Tableau des résultats pour analyte 84 -- suite de la page précédente			
**Classement (Qualité)**	**Profil de référence**	**Score Valeur**	**Identifiant NCBI**
10<br>(+)	Bacillus subtilis ssp subtilis DSM 10T DSM	1.79	135461

Analyte 85

**Nom de l'échantillon:**	H1
**Description de l'échantillon:**	
**ID de l'échantillon:**	H1
**Date/Heure de création de l'échantillon:**	2019-07-10T17:04:20.734
**Type de l'échantillon:**	Échantillon standard
**Méthode de classification :**	MALDI Biotyper MSP Identification Standard Method 1.1
**Méthode de prétraitement :**	BioTyper Preprocessing Standard Method 1.2
**Méthode ACQ :**	D:\Methods\flexControlMethods\MBT_FC.par
**Horodatage ACQ :**	2019-07-10T17:34:47.096
**Méthode AutoXecute :**	MBT_AutoX_smart
**Bibliothèque de MSP utilisée:**	Culturomics / f8c211c3-71c5-471b-8a7e-7f6abca59bb9 / 2019-06-28T14:15:05.935, Timone / 29617d84-2a1e-4bf6-a13d-569eecb48f06 / 2018-04-19T13:24:29.884, BDAL / contains 7854 MSPs / e7ef41ca-b750-4d47-9a1c-6c26fa454356 / 2019-02-01T09:48:20.358

Classement (Qualité)	Profil de référence	Score Valeur	Identifiant NCBI
1 (+++)	Bacillus subtilis CD15-3	2.05	133993714
2 (+++)	Bacillus subtilis strain CD7.3	2.03	133993714
3 (+)	Bacillus subtilis CSURP291	1.87	130148166
4 (+)	Bacillus subtilis DSM 5611 DSM	1.79	1423
5 (+)	Bacillus subtilis ssp subtilis DSM 5660 DSM	1.78	135461
6 (+)	Bacillus subtilis ssp subtilis DSM 10T DSM	1.76	135461
7 (+)	Lactobacillus fermentum CSURP4362	1.74	130148166
8 (-)	Bacillus vallismortis CSURP984	1.69	130148166
9 (-)	Bacillus subtilis ssp subtilis CICC 23950 CICC	1.62	135461

Tableau des résultats pour analyte 85--suite page suivante

Tableau des résultats pour analyte 85 -- suite de la page précédente			
**Classement (Qualité)**	**Profil de référence**	**Score Valeur**	**Identifiant NCBI**
10 (-)	Bacillus mojavensis CSURP1524	1.61	130148166

Analyte 86

**Nom de l'échantillon:**	H2
**Description de l'échantillon:**	
**ID de l'échantillon:**	H2
**Date/Heure de création de l'échantillon:**	2019-07-10T17:04:20.735
**Type de l'échantillon:**	Échantillon standard
**Méthode de classification :**	MALDI Biotyper MSP Identification Standard Method 1.1
**Méthode de prétraitement :**	BioTyper Preprocessing Standard Method 1.2
**Méthode ACQ :**	D:\Methods\flexControlMethods\MBT_FC.par
**Horodatage ACQ :**	2019-07-10T17:35:07.967
**Méthode AutoXecute :**	MBT_AutoX_smart
**Bibliothèque de MSP utilisée:**	Culturomics / f8c211c3-71c5-471b-8a7e-7f6abca59bb9 / 2019-06-28T14:15:05.935, Timone / 29617d84-2a1e-4bf6-a13d-569eecb48f06 / 2018-04-19T13:24:29.884, BDAL / contains 7854 MSPs / e7ef41ca-b750-4d47-9a1c-6c26fa454356 / 2019-02-01T09:48:20.358

Classement (Qualité)	Profil de référence	Score Valeur	Identifiant NCBI
1 (-)	Staphylococcus cohnii ssp urealyticus DSM 6718T DSM	1.33	94138
2 (-)	Lactobacillus curvatus DSM 20495 DSM	1.33	28038
3 (-)	Arthrobacter luteolus DSM 13067T DSM	1.31	98672
4 (-)	Filifactor villosus 1051_NCTC 11220T BOG	1.31	29374
5 (-)	Candida tropicalis VML	1.30	5482
6 (-)	Lactobacillus paracasei ssp paracasei DSM 20207 DSM	1.30	47714
7 (-)	Arthrobacter pascens DSM 20545T DSM	1.27	1677
8 (-)	Lactobacillus sakei ssp sakei DSM 20017T DSM	1.27	214326
9 (-)	Aeromonas veronii CECT 5761T DSM	1.24	654

Tableau des résultats pour analyte 86--suite page suivante

Tableau des résultats pour analyte 86 -- suite de la page précédente			
**Classement (Qualité)**	**Profil de référence**	**Score Valeur**	**Identifiant NCBI**
10 (-)	Aeromonas encheleia CECT 4342T DSM	1.24	73010

Analyte 87

**Nom de l'échantillon:**	H3
**Description de l'échantillon:**	
**ID de l'échantillon:**	H3
**Date/Heure de création de l'échantillon:**	2019-07-10T17:04:20.737
**Type de l'échantillon:**	Échantillon standard
**Méthode de classification :**	MALDI Biotyper MSP Identification Standard Method 1.1
**Méthode de prétraitement :**	BioTyper Preprocessing Standard Method 1.2
**Méthode ACQ :**	D:\Methods\flexControlMethods\MBT_FC.par
**Horodatage ACQ :**	2019-07-10T17:35:28.947
**Méthode AutoXecute :**	MBT_AutoX_smart
**Bibliothèque de MSP utilisée:**	Culturomics / f8c211c3-71c5-471b-8a7e-7f6abca59bb9 / 2019-06-28T14:15:05.935, Timone / 29617d84-2a1e-4bf6-a13d-569eecb48f06 / 2018-04-19T13:24:29.884, BDAL / contains 7854 MSPs / e7ef41ca-b750-4d47-9a1c-6c26fa454356 / 2019-02-01T09:48:20.358

Classement (Qualité)	Profil de référence	Score Valeur	Identifiant NCBI
1 (-)	Arthrobacter ramosus IMET 10685T HKJ	1.34	1672
2 (-)	Citrobacter freundii 13158_2 CHB	1.31	546
3 (-)	Lactobacillus intestinalis DSM 6629T DSM	1.28	151781
4 (-)	Terrimonas ferruginea DSM 30193T HAM	1.26	249
5 (-)	Bacillus pumilus 10403329	1.25	133055080
6 (-)	Staphylococcus pasteuri DSM 10656T DSM	1.24	45972
7 (-)	Anaerococcus vaginalis 8270201	1.21	133993714
8 (-)	Agromyces humatus HKI 327 HKJ	1.21	279573
9 (-)	Flavobacterium flevense DSM 1076T HAM	1.21	983

Tableau des résultats pour analyte 87--suite page suivante

Tableau des résultats pour analyte 87 -- suite de la page précédente			
**Classement (Qualité)**	**Profil de référence**	**Score Valeur**	**Identifiant NCBI**
10<br>(-)	Clostridium cadaveris 1074_ATCC 25783T BOG	1.20	1529

Analyte 88

**Nom de l'échantillon:**	H4
**Description de l'échantillon:**	
**ID de l'échantillon:**	H4
**Date/Heure de création de l'échantillon:**	2019-07-10T17:04:20.739
**Type de l'échantillon:**	Échantillon standard
**Méthode de classification :**	MALDI Biotyper MSP Identification Standard Method 1.1
**Méthode de prétraitement :**	BioTyper Preprocessing Standard Method 1.2
**Méthode ACQ :**	D:\Methods\flexControlMethods\MBT_FC.par
**Horodatage ACQ :**	2019-07-10T17:35:50.119
**Méthode AutoXecute :**	MBT_AutoX_smart
**Bibliothèque de MSP utilisée:**	Culturomics / f8c211c3-71c5-471b-8a7e-7f6abca59bb9 / 2019-06-28T14:15:05.935, Timone / 29617d84-2a1e-4bf6-a13d-569eecb48f06 / 2018-04-19T13:24:29.884, BDAL / contains 7854 MSPs / e7ef41ca-b750-4d47-9a1c-6c26fa454356 / 2019-02-01T09:48:20.358

Classement (Qualité)	Profil de référence	Score Valeur	Identifiant NCBI
1 (+)	Bacillus subtilis strain CD7.3	1.99	133993714
2 (+)	Bacillus subtilis CSURP3865	1.89	130148166
3 (+)	Bacillus subtilis CSURP291	1.84	130148166
4 (+)	Bacillus subtilis DSM 5552 DSM	1.73	1423
5 (-)	Bacillus subtilis ssp subtilis DSM 5660 DSM	1.66	135461
6 (-)	Bacillus mojavensis CSURP1524	1.64	130148166
7 (-)	Bacillus subtilis DSM 5611 DSM	1.61	1423
8 (-)	Bacillus subtilis ssp spizizenii DSM 15029T DSM	1.59	96241
9 (-)	Bacillus subtilis CD15-3	1.58	133993714

Tableau des résultats pour analyte 88--suite page suivante

Tableau des résultats pour analyte 88 -- suite de la page précédente			
**Classement (Qualité)**	**Profil de référence**	**Score Valeur**	**Identifiant NCBI**
10<br>(-)	Bacillus subtilis ssp subtilis DSM 10T DSM	1.58	135461

Analyte 89

**Nom de l'échantillon:**	H5
**Description de l'échantillon:**	
**ID de l'échantillon:**	H5
**Date/Heure de création de l'échantillon:**	2019-07-10T17:04:20.741
**Type de l'échantillon:**	Échantillon standard
**Méthode de classification :**	MALDI Biotyper MSP Identification Standard Method 1.1
**Méthode de prétraitement :**	BioTyper Preprocessing Standard Method 1.2
**Méthode ACQ :**	D:\Methods\flexControlMethods\MBT_FC.par
**Horodatage ACQ :**	2019-07-10T17:36:10.396
**Méthode AutoXecute :**	MBT_AutoX_smart
**Bibliothèque de MSP utilisée:**	Culturomics / f8c211c3-71c5-471b-8a7e-7f6abca59bb9 / 2019-06-28T14:15:05.935, Timone / 29617d84-2a1e-4bf6-a13d-569eecb48f06 / 2018-04-19T13:24:29.884, BDAL / contains 7854 MSPs / e7ef41ca-b750-4d47-9a1c-6c26fa454356 / 2019-02-01T09:48:20.358

Classement (Qualité)	Profil de référence	Score Valeur	Identifiant NCBI
1 (-)	Bacillus licheniformis	1.46	133993714
2 (-)	Lactobacillus pentosus DSM 20199 DSM	1.34	1589
3 (-)	Acidovorax temperans DSM 7270T HAM	1.25	80878
4 (-)	Lactobacillus plantarum ssp plantarum DSM 20174T DSM	1.25	337330
5 (-)	Lactobacillus salivarius DSM 20555T DSM	1.24	1624
6 (-)	Legionella pneumophila ssp pneumophila ATCC 33152T THL	1.21	446
7 (-)	Lactobacillus pentosus DSM 20314T DSM	1.20	1589
8 (-)	Staphylococcus simulans DSM 20322T DSM	1.20	1286
9 (-)	Lactobacillus coryniformis ssp coryniformis DSM 20007 DSM	1.20	115541

Tableau des résultats pour analyte 89--suite page suivante

Tableau des résultats pour analyte 89 -- suite de la page précédente			
**Classement (Qualité)**	**Profil de référence**	**Score Valeur**	**Identifiant NCBI**
10<br>(-)	Sodalis glossinidius DSM 16929T HAM	1.19	63612

Analyte 90

**Nom de l'échantillon:**	H6
**Description de l'échantillon:**	
**ID de l'échantillon:**	H6
**Date/Heure de création de l'échantillon:**	2019-07-10T17:04:20.744
**Type de l'échantillon:**	Échantillon standard
**Méthode de classification :**	MALDI Biotyper MSP Identification Standard Method 1.1
**Méthode de prétraitement :**	BioTyper Preprocessing Standard Method 1.2
**Méthode ACQ :**	D:\Methods\flexControlMethods\MBT_FC.par
**Horodatage ACQ :**	2019-07-10T17:36:31.362
**Méthode AutoXecute :**	MBT_AutoX_smart
**Bibliothèque de MSP utilisée:**	Culturomics / f8c211c3-71c5-471b-8a7e-7f6abca59bb9 / 2019-06-28T14:15:05.935, Timone / 29617d84-2a1e-4bf6-a13d-569eecb48f06 / 2018-04-19T13:24:29.884, BDAL / contains 7854 MSPs / e7ef41ca-b750-4d47-9a1c-6c26fa454356 / 2019-02-01T09:48:20.358

Classement (Qualité)	Profil de référence	Score Valeur	Identifiant NCBI
1 (+)	Bacillus licheniformis	1.70	133993714
2 (-)	Bacillus sonorensis DSM 13779T DSM	1.48	119858
3 (-)	Enterococcus faecalis ATCC 29212 CHB	1.40	1351
4 (-)	Lactobacillus pentosus DSM 20199 DSM	1.36	1589
5 (-)	Propionibacterium acnes 8246983	1.34	133055080
6 (-)	Hydrogenophaga flava B339 UFL	1.34	65657
7 (-)	Lactobacillus plantarum ssp plantarum DSM 20174T DSM	1.30	337330
8 (-)	Bacillus licheniformis CS 54_1 BRB	1.29	1402
9 (-)	Staphylococcus sciuri ssp sciuri DSM 20345T DSM	1.28	147467

Tableau des résultats pour analyte 90--suite page suivante

Tableau des résultats pour analyte 90 -- suite de la page précédente			
**Classement (Qualité)**	**Profil de référence**	**Score Valeur**	**Identifiant NCBI**
10<br>(-)	Pseudarthrobacter sulfonivorans DSM 14002T DSM	1.28	121292

Analyte 91

**Nom de l’échantillon:**	H7
**Description de l’échantillon:**	
**ID de l’échantillon:**	H7
**Date/Heure de création de l’échantillon:**	2019-07-10T17:04:20.746
**Type de l’échantillon:**	Échantillon standard
**Méthode de classification :**	MALDI Biotyper MSP Identification Standard Method 1.1
**Méthode de prétraitement :**	BioTyper Preprocessing Standard Method 1.2
**Méthode ACQ :**	D:\Methods\flexControlMethods\MBT_FC.par
**Horodatage ACQ :**	2019-07-10T17:36:52.658
**Méthode AutoXecute :**	MBT_AutoX_smart
**Bibliothèque de MSP utilisée:**	Culturomics / f8c211c3-71c5-471b-8a7e-7f6abca59bb9 / 2019-06-28T14:15:05.935, Timone / 29617d84-2a1e-4bf6-a13d-569eecb48f06 / 2018-04-19T13:24:29.884, BDAL / contains 7854 MSPs / e7ef41ca-b750-4d47-9a1c-6c26fa454356 / 2019-02-01T09:48:20.358

Classement (Qualité)	Profil de référence	Score Valeur	Identifiant NCBI
1 (-)	Bacillus licheniformis	1.53	133993714
2 (-)	Pseudomonas granadensis CSURP8718	1.43	130148166
3 (-)	Lactobacillus pentosus DSM 20199 DSM	1.43	1589
4 (-)	Propionibacterium acnes 8246983	1.38	133055080
5 (-)	Helicobacter canis CIP 104753T CBB	1.34	29419
6 (-)	Lactobacillus satsumensis DSM 16230T DSM	1.29	259059
7 (-)	Lactobacillus salivarius DSM 20492 DSM	1.27	1624
8 (-)	Lactobacillus murinus DSM 20452T DSM	1.24	1622
9 (-)	Staphylococcus sciuri ssp sciuri DSM 20345T DSM	1.24	147467

Tableau des résultats pour analyte 91--suite page suivante

Tableau des résultats pour analyte 91 -- suite de la page précédente			
**Classement (Qualité)**	**Profil de référence**	**Score Valeur**	**Identifiant NCBI**
10<br>(-)	Lactobacillus plantarum ssp plantarum DSM 20174T DSM	1.24	337330

Analyte 92

**Nom de l'échantillon:**	H8
**Description de l'échantillon:**	
**ID de l'échantillon:**	H8
**Date/Heure de création de l'échantillon:**	2019-07-10T17:04:20.749
**Type de l'échantillon:**	Échantillon standard
**Méthode de classification :**	MALDI Biotyper MSP Identification Standard Method 1.1
**Méthode de prétraitement :**	BioTyper Preprocessing Standard Method 1.2
**Méthode ACQ :**	D:\Methods\flexControlMethods\MBT_FC.par
**Horodatage ACQ :**	2019-07-10T17:37:13.329
**Méthode AutoXecute :**	MBT_AutoX_smart
**Bibliothèque de MSP utilisée:**	Culturomics / f8c211c3-71c5-471b-8a7e-7f6abca59bb9 / 2019-06-28T14:15:05.935, Timone / 29617d84-2a1e-4bf6-a13d-569eecb48f06 / 2018-04-19T13:24:29.884, BDAL / contains 7854 MSPs / e7ef41ca-b750-4d47-9a1c-6c26fa454356 / 2019-02-01T09:48:20.358

Classement (Qualité)	Profil de référence	Score Valeur	Identifiant NCBI
1 (+++)	Escherichia coli CSURP4799	2.36	130148166
2 (+++)	Escherichia coli CSURP420	2.31	130148166
3 (+++)	Escherichia coli CSURP427	2.30	130148166
4 (+++)	Escherichia coli CSURP426	2.26	130148166
5 (+++)	Escherichia coli CSURP428	2.26	130148166
6 (+++)	Escherichia coli CSURP1872	2.25	130148166
7 (+++)	Escherichia coli CSURP422	2.24	130148166
8 (+++)	Escherichia coli CSURP3866	2.24	130148166
9 (+++)	Escherichia coli CSURP397	2.22	130148166

Tableau des résultats pour analyte 92--suite page suivante

Tableau des résultats pour analyte 92 -- suite de la page précédente			
**Classement (Qualité)**	**Profil de référence**	**Score Valeur**	**Identifiant NCBI**
10 (+++)	Escherichia coli CSURP415	2.21	130148166

Analyte 93

**Nom de l'échantillon:**	H9
**Description de l'échantillon:**	
**ID de l'échantillon:**	H9
**Date/Heure de création de l'échantillon:**	2019-07-10T17:04:20.750
**Type de l'échantillon:**	Échantillon standard
**Méthode de classification :**	MALDI Biotyper MSP Identification Standard Method 1.1
**Méthode de prétraitement :**	BioTyper Preprocessing Standard Method 1.2
**Méthode ACQ :**	D:\Methods\flexControlMethods\MBT_FC.par
**Horodatage ACQ :**	2019-07-10T17:37:31.559
**Méthode AutoXecute :**	MBT_AutoX_smart
**Bibliothèque de MSP utilisée:**	Culturomics / f8c211c3-71c5-471b-8a7e-7f6abca59bb9 / 2019-06-28T14:15:05.935, Timone / 29617d84-2a1e-4bf6-a13d-569eecb48f06 / 2018-04-19T13:24:29.884, BDAL / contains 7854 MSPs / e7ef41ca-b750-4d47-9a1c-6c26fa454356 / 2019-02-01T09:48:20.358

Classement (Qualité)	Profil de référence	Score Valeur	Identifiant NCBI
1 (+++)	Escherichia coli CSURP4799	2.39	130148166
2 (+++)	Escherichia coli CSURP1872	2.39	130148166
3 (+++)	Escherichia coli CSURP2202	2.35	130148166
4 (+++)	Escherichia coli CSURP1570	2.35	130148166
5 (+++)	Escherichia coli CSURP422	2.33	130148166
6 (+++)	Escherichia coli CIP7624 lot 561	2.32	133055080
7 (+++)	Escherichia coli CSURP2701	2.27	130148166
8 (+++)	Escherichia coli CSURP4969	2.25	130148166
9 (+++)	Escherichia coli ATCC 25922 THL	2.23	562

Tableau des résultats pour analyte 93--suite page suivante

Tableau des résultats pour analyte 93 -- suite de la page précédente			
**Classement (Qualité)**	**Profil de référence**	**Score Valeur**	**Identifiant NCBI**
10 (+++)	Escherichia coli CSURP415	2.22	130148166

Analyte 94

**Nom de l'échantillon:**	H10
**Description de l'échantillon:**	
**ID de l'échantillon:**	H10
**Date/Heure de création de l'échantillon:**	2019-07-10T17:04:20.752
**Type de l'échantillon:**	Échantillon standard
**Méthode de classification :**	MALDI Biotyper MSP Identification Standard Method 1.1
**Méthode de prétraitement :**	BioTyper Preprocessing Standard Method 1.2
**Méthode ACQ :**	D:\Methods\flexControlMethods\MBT_FC.par
**Horodatage ACQ :**	2019-07-10T17:37:50.277
**Méthode AutoXecute :**	MBT_AutoX_smart
**Bibliothèque de MSP utilisée:**	Culturomics / f8c211c3-71c5-471b-8a7e-7f6abca59bb9 / 2019-06-28T14:15:05.935, Timone / 29617d84-2a1e-4bf6-a13d-569eecb48f06 / 2018-04-19T13:24:29.884, BDAL / contains 7854 MSPs / e7ef41ca-b750-4d47-9a1c-6c26fa454356 / 2019-02-01T09:48:20.358

Classement (Qualité)	Profil de référence	Score Valeur	Identifiant NCBI
1 (+++)	Escherichia coli CSURP4799	2.33	130148166
2 (+++)	Escherichia coli CSURP399	2.27	130148166
3 (+++)	Escherichia coli CSURP420	2.22	130148166
4 (+++)	Escherichia coli CSURP1872	2.20	130148166
5 (+++)	Escherichia coli CSURP3223	2.20	130148166
6 (+++)	Escherichia coli CSURP415	2.19	130148166
7 (+++)	Escherichia coli CSURP3247	2.16	130148166
8 (+++)	Escherichia coli CSURP422	2.16	130148166
9 (+++)	Escherichia coli CSURP3866	2.16	130148166

Tableau des résultats pour analyte 94--suite page suivante

Tableau des résultats pour analyte 94 -- suite de la page précédente			
**Classement (Qualité)**	**Profil de référence**	**Score Valeur**	**Identifiant NCBI**
10 (+++)	Escherichia coli CSURP1570	2.14	130148166

Analyte 95

**Nom de l'échantillon:**	H11
**Description de l'échantillon:**	
**ID de l'échantillon:**	H11
**Date/Heure de création de l'échantillon:**	2019-07-10T17:04:20.754
**Type de l'échantillon:**	Échantillon standard
**Méthode de classification :**	MALDI Biotyper MSP Identification Standard Method 1.1
**Méthode de prétraitement :**	BioTyper Preprocessing Standard Method 1.2
**Méthode ACQ :**	D:\Methods\flexControlMethods\MBT_FC.par
**Horodatage ACQ :**	2019-07-10T17:38:11.675
**Méthode AutoXecute :**	MBT_AutoX_smart
**Bibliothèque de MSP utilisée:**	Culturomics / f8c211c3-71c5-471b-8a7e-7f6abca59bb9 / 2019-06-28T14:15:05.935, Timone / 29617d84-2a1e-4bf6-a13d-569eecb48f06 / 2018-04-19T13:24:29.884, BDAL / contains 7854 MSPs / e7ef41ca-b750-4d47-9a1c-6c26fa454356 / 2019-02-01T09:48:20.358

Classement (Qualité)	Profil de référence	Score Valeur	Identifiant NCBI
1 (-)	Agromyces rhizospherae HKI 302_DSM 14597T HKJ	1.49	88374
2 (-)	Lactobacillus paracasei subsp paracasei 103918T CIP	1.32	133055080
3 (-)	Salinivibrio costicola CSUR P2423	1.31	130148166
4 (-)	Rhizobium radiobacter B177 UFL	1.27	358
5 (-)	Candida lusitaniae CBS 4413T CBS	1.26	36911
6 (-)	Mycobacterium avium hominissuis 10400589	1.24	133055080
7 (-)	Lactobacillus parabuchneri DSM 15352 DSM	1.23	152331
8 (-)	Candida krusei ATCC 6258 THL	1.22	4909
9 (-)	Microbacterium saperdae IMET 11076T HKJ	1.21	69368

Tableau des résultats pour analyte 95--suite page suivante

Tableau des résultats pour analyte 95 -- suite de la page précédente			
**Classement (Qualité)**	**Profil de référence**	**Score Valeur**	**Identifiant NCBI**
10<br>(-)	Staphylococcus auricularis BK04812_08 ERL	1.18	29379

Analyte 96

**Nom de l'échantillon:**	H12
**Description de l'échantillon:**	
**ID de l'échantillon:**	H12
**Date/Heure de création de l'échantillon:**	2019-07-10T17:04:20.756
**Type de l'échantillon:**	Échantillon standard
**Méthode de classification :**	MALDI Biotyper MSP Identification Standard Method 1.1
**Méthode de prétraitement :**	BioTyper Preprocessing Standard Method 1.2
**Méthode ACQ :**	D:\Methods\flexControlMethods\MBT_FC.par
**Horodatage ACQ :**	2019-07-10T17:38:32.460
**Méthode AutoXecute :**	MBT_AutoX_smart
**Bibliothèque de MSP utilisée:**	Culturomics / f8c211c3-71c5-471b-8a7e-7f6abca59bb9 / 2019-06-28T14:15:05.935, Timone / 29617d84-2a1e-4bf6-a13d-569eecb48f06 / 2018-04-19T13:24:29.884, BDAL / contains 7854 MSPs / e7ef41ca-b750-4d47-9a1c-6c26fa454356 / 2019-02-01T09:48:20.358

Classement (Qualité)	Profil de référence	Score Valeur	Identifiant NCBI
1 (-)	Bacillus vallismortis strain CN13.1	1.30	133993714
2 (-)	Candida lusitaniae CBS 4413T CBS	1.23	36911
3 (-)	Actinomyces turicensis CSURP5607	1.23	130148166
4 (-)	Enterococcus faecium VRE_PX_16086218 MLD	1.22	1352
5 (-)	Filifactor villosus 1051_NCTC 11220T BOG	1.22	29374
6 (-)	Pseudarthrobacter oxydans DSM 20119T DSM	1.22	1671
7 (-)	Pseudarthrobacter polychromogenes DSM 20136T DSM	1.19	1676
8 (-)	Lactobacillus paralimentarius DSM 13961 DSM	1.18	83526
9 (-)	Mycobacterium boenickei DSM 44677T DSM	1.17	146017

Tableau des résultats pour analyte 96--suite page suivante

Tableau des résultats pour analyte 96 -- suite de la page précédente			
**Classement (Qualité)**	**Profil de référence**	**Score Valeur**	**Identifiant NCBI**
10<br>(-)	Pantoea anthrophila CSURP3653	1.16	130148166
