# Supplementary material for: Characterization of Gut Bacteria in Natural Populations of Sand Flies (Diptera: Psychodidae) from Endemic and Non-Endemic Areas of Leishmaniasis in Morocco
Source: Microorganisms. 2025 Sep 30;13(10):2279. doi: 10.3390/microorganisms13102279 (PMC12565922; doi:10.3390/microorganisms13102279)
Supplement: Supplementary file 1 [file microorganisms-13-02279-s001.zip › microorganisms-3810545 S2.pdf]

Bruker Daltonik MALDI Biotyper
Résultats d’identification

Détails du projet:

**Nom du projet:**	190710-1841-10102301
**Description du projet:**	
**Auteur du projet:**	tof-user@STVPR-FLEX-PC1
**Date/Heure de création du projet:**	2019-07-10T18:42:16.540
**Nombre d’échantillons :**	48
**Type :**	Échantillon standard
**BTS-QC :**	pas existant
**Position BTS-QC :**	
**ID instrument :**	254472.00200
**Version du serveur :**	4.1.80 (PYTH) 102 2017-08-226_04-55-52

Aperçu des résultats

Nom de l’échantillon	ID de l’échantillon	Organisme (meilleur candidat)	Score Valeur	Organisme (second candidat)	Score Valeur
A1 (+++) (A)	A1 (standard)	Bacillus pumilus	2.32	Bacillus pumilus	2.25
A2 (-) (C)	A2 (standard)	**Aucune identification possible**	1.58	**Aucune identification possible**	1.54
A3 (-) (C)	A3 (standard)	**Aucune identification possible**	1.39	**Aucune identification possible**	1.33
A4 (-) (C)	A4 (standard)	**Aucune identification possible**	1.35	**Aucune identification possible**	1.31
A5 (-) (C)	A5 (standard)	**Aucune identification possible**	1.15	**Aucune identification possible**	1.13
A6 (+++) (A)	A6 (standard)	Bacillus pumilus	2.02	Bacillus pumilus	1.97

Aperçu des résultats -- suite page suivante

Aperçu des résultats -- suite de la page précédente					
**Nom de l'échantillon**	**ID de l'échantillon**	**Organisme (meilleur candidat)**	**Score Valeur**	**Organisme (second candidat)**	**Score Valeur**
A7 (+++) (A)	A7 (standard)	Bacillus pumilus	2.20	Bacillus pumilus	2.17
A8 (+++) (A)	A8 (standard)	Bacillus pumilus	2.20	Bacillus pumilus	2.20
A9 (+++) (A)	A9 (standard)	Bacillus pumilus	2.11	Bacillus pumilus	2.08
A10 (+++) (A)	A10 (standard)	Bacillus pumilus	2.06	Bacillus pumilus	1.88
A11 (+++) (A)	A11 (standard)	Bacillus pumilus	2.61	Bacillus pumilus	2.49
A12 (+++) (A)	A12 (standard)	Bacillus pumilus	2.16	Bacillus pumilus	1.99
B1 (+++) (A)	B1 (standard)	Bacillus subtilis	2.35	Bacillus subtilis	2.05
B2 (+++) (C)	B2 (standard)	Lactobacillus fermentum	2.15	Bacillus subtilis	2.14
B3 (+) (B)	B3 (standard)	Bacillus pumilus	1.99	Bacillus pumilus	1.84
B4 (+++) (A)	B4 (standard)	Bacillus pumilus	2.47	Bacillus pumilus	2.47
B5 (-) (C)	B5 (standard)	**Aucune identification possible**	1.51	**Aucune identification possible**	1.37
B6 (-) (C)	B6 (standard)	**Aucune identification possible**	1.66	**Aucune identification possible**	1.57
B7 (+++) (A)	B7 (standard)	Lysinibacillus fusiformis	2.11	Lysinibacillus fusiformis	2.08
B8 (+++) (B)	B8 (standard)	Lysinibacillus boronitolerans	2.16	Lysinibacillus fusiformis	2.09
B9 (-) (C)	B9 (standard)	**Aucune identification possible**	1.19	**Aucune identification possible**	1.14
B10 (-) (C)	B10 (standard)	**Aucune identification possible**	1.65	**Aucune identification possible**	1.62
Aperçu des résultats -- suite page suivante					

Aperçu des résultats -- suite de la page précédente					
**Nom de l'échantillon**	**ID de l'échantillon**	**Organisme (meilleur candidat)**	**Score Valeur**	**Organisme (second candidat)**	**Score Valeur**
B11 (+++) (A)	B11 (standard)	Bacillus pumilus	2.25	Bacillus pumilus	2.00
B12 (+++) (A)	B12 (standard)	Bacillus pumilus	2.04	Bacillus pumilus	2.00
C1 (-) (C)	C1 (standard)	**Aucune identification possible**	1.15	**Aucune identification possible**	1.11
C2 (-) (C)	C2 (standard)	**Aucune identification possible**	1.19	**Aucune identification possible**	1.09
C3 (+++) (A)	C3 (standard)	Bacillus pumilus	2.43	Bacillus pumilus	2.20
C4 (+) (B)	C4 (standard)	Bacillus pumilus	1.92	Bacillus pumilus	1.77
C5 (+++) (A)	C5 (standard)	Staphylococcus lentus	2.17	Staphylococcus lentus	2.13
C6 (+++) (A)	C6 (standard)	Staphylococcus lentus	2.41	Staphylococcus lentus	2.28
C7 (+++) (A)	C7 (standard)	Bacillus pumilus	2.19	Bacillus pumilus	2.17
C8 (+) (B)	C8 (standard)	Bacillus pumilus	1.78	Bacillus pumilus	1.74
C9 (+++) (A)	C9 (standard)	Bacillus subtilis	2.35	Bacillus subtilis	2.26
C10 (+++) (A)	C10 (standard)	Bacillus subtilis	2.30	Bacillus subtilis	2.15
C11 (-) (C)	C11 (standard)	**Aucune identification possible**	1.46	**Aucune identification possible**	1.39
C12 (+++) (A)	C12 (standard)	Bacillus subtilis	2.33	Bacillus subtilis	2.17
D1 (+) (B)	D1 (standard)	Bacillus subtilis	1.88	Bacillus subtilis	1.78
D2 (+) (B)	D2 (standard)	Bacillus subtilis	1.93	**Aucune identification possible**	1.56
Aperçu des résultats -- suite page suivante					

Aperçu des résultats -- suite de la page précédente

Nom de l'échantillon	ID de l'échantillon	Organisme (meilleur candidat)	Score Valeur	Organisme (second candidat)	Score Valeur
D3 (+++) (A)	D3 (standard)	Bacillus pumilus	2.33	Bacillus pumilus	2.32
D4 (+++) (A)	D4 (standard)	Bacillus pumilus	2.11	Bacillus pumilus	2.07
D5 (-) (C)	D5 (standard)	**Aucune identification possible**	1.39	**Aucune identification possible**	1.32
D6 (-) (C)	D6 (standard)	**Aucune identification possible**	1.37	**Aucune identification possible**	1.31
D7 (+++) (A)	D7 (standard)	Proteus vulgaris	2.09	Proteus vulgaris	2.09
D8 (+) (B)	D8 (standard)	Proteus vulgaris	1.77	**Aucune identification possible**	1.65
D9 (-) (C)	D9 (standard)	**Aucune identification possible**	1.57	**Aucune identification possible**	1.48
D10 (+) (B)	D10 (standard)	Proteus hauseri	1.96	Proteus hauseri	1.91
D11 (+) (B)	D11 (standard)	Morganella morganii	1.80	Morganella morganii	1.78
D12 (+) (B)	D12 (standard)	Morganella morganii	1.89	Morganella morganii	1.83

Indication de référence

Profil de référence	Commentaire
Acinetobacter gerneri DSM 14967T HAM	Les profils des espèces appartenant à ce genre sont très ressemblants: la différentiation des espèces est donc difficile.
Acinetobacter tandoii DSM 14970T HAM	Les profils des espèces appartenant à ce genre sont très ressemblants: la différentiation des espèces est donc difficile.
Aeromonas schubertii CECT 4240T DSM	Les profils des espèces appartenant à ce genre sont très ressemblants: la différentiation des espèces est donc difficile.
Bacillus atrophaeus DSM 5551 DSM	appartient au groupe Bacillus subtilis. La qualité des spectres (qui influe sur le score) dépend du degré de sporulation: Utilisez des colonies fraîches.
Bacillus cereus DSM 31T DSM	Bacillus anthracis, cereus, mycoides, pseudomycoides, thuringiensis et weihenstephanensis sont proches et appartiennent au groupe Bacillus cereus. En particulier les spectres de Bacillus cereus sont très similaires à ceux de Bacillus anthracis. Bacillus anthracis n'est pas inclus dans la base de données du MALDI Biotyper. Pour une différentiation une méthode adéquate doit être sélectionnée par un professionnel expérimenté. La qualité des spectres (score) dépende du degré de sporulation: utiliser des cultures fraîches.
Bacillus koreensis DSM 16467T DSM	La qualité des spectres (qui influe sur le score) dépend du degré de sporulation: Utilisez des colonies fraîches.
Bacillus mojavensis DSM 9205T DSM	appartient au groupe Bacillus subtilis. La qualité des spectres (qui influe sur le score) dépend du degré de sporulation: Utilisez des colonies fraîches.
Bacillus muralis DSM 16288T DSM	La qualité des spectres (qui influe sur le score) dépend du degré de sporulation: Utilisez des colonies fraîches.
Bacillus pumilus DSM 354 DSM	La qualité des spectres (qui influe sur le score) dépend du degré de sporulation: Utilisez des colonies fraîches.
Bacillus subtilis DSM 5552 DSM	appartient au groupe Bacillus subtilis. La qualité des spectres (qui influe sur le score) dépend du degré de sporulation: Utilisez des colonies fraîches.
Bacillus subtilis DSM 5611 DSM	appartient au groupe Bacillus subtilis. La qualité des spectres (qui influe sur le score) dépend du degré de sporulation: Utilisez des colonies fraîches.
Bacillus subtilis ssp spizizenii DSM 15029T DSM	appartient au groupe Bacillus subtilis. La qualité des spectres (qui influe sur le score) dépend du degré de sporulation: Utilisez des colonies fraîches.
Bacillus subtilis ssp subtilis DSM 10T DSM	appartient au groupe Bacillus subtilis. La qualité des spectres (qui influe sur le score) dépend du degré de sporulation: Utilisez des colonies fraîches.
Bacillus subtilis ssp subtilis DSM 5660 DSM	appartient au groupe Bacillus subtilis. La qualité des spectres (qui influe sur le score) dépend du degré de sporulation: Utilisez des colonies fraîches.

Indication de référence --suite page suivante

Indication de référence -- suite de la page précédente	
**Profil de référence**	**Commentaire**
Bacillus weihenstephanensis DSM 11821T DSM	Bacillus anthracis, cereus, mycoides, pseudomycoides, thuringiensis et weihenstephanensis sont proches et appartiennent au groupe Bacillus cereus. En particulier les spectres de Bacillus cereus sont très similaires à ceux de Bacillus anthracis. Bacillus anthracis n'est pas inclus dans la base de données du MALDI Biotyper. Pour une différentiation une méthode adéquate doit être sélectionnée par un professionnel expérimenté. La qualité des spectres (score) dépende du degré de sporulation: utiliser des cultures fraîches.
Burkholderia cenocepacia LMG 12614 HAM	appartient au complexe Burkholderia cepacia
Burkholderia seminalis VA40474_09 ERL	appartient au complexe Burkholderia cepacia
Clostridium beijerinckii 1072_ATCC 25752T BOG	Les espèces beijerinckii / diolis du genre Clostridium présentent des profils très similaires : il est donc difficile de distinguer ces espèces.
Clostridium clostridioforme CCUG 38271 CCUG	Les espèces bolteae / clostridioforme du genre Clostridium présentent des profils très similaires : il est donc difficile de distinguer ces espèces.
Escherichia coli ATCC 35218 CHB	proche de Shigella / Escherichia fergusonii mais ne peut en être distingué avec certitude pour le moment
Escherichia coli MB11464_1 CHB	proche de Shigella / Escherichia fergusonii mais ne peut en être distingué avec certitude pour le moment
Fusobacterium naviforme DSM 20699 BRB	Les espèces naviforme / nucleatum du genre Fusobacterium présentent des profils très similaires : il est donc difficile de distinguer ces espèces.
Klebsiella aerogenes 15282_1 CHB	Synonyme de Enterobacter aerogenes
Klebsiella oxytoca ATCC 700324 THL	Les espèces Klebsiella oxytoca et ornithinolytica / planticola / terrigena du genre Raoultella présentent des profils très similaires : il est donc difficile de distinguer ces espèces.
Klebsiella pneumoniae ssp pneumoniae 9295_1 CHB	proche de Klebsiella variicola
Lysinibacillus boronitolerans DSM 17140T DSM	Les espèces boronitolerans / xylanilyticus du genre Lysinibacillus présentent des profils très similaires : il est donc difficile de distinguer ces espèces. La qualité des spectres (qui influe sur le score) dépend du degré de sporulation: Utilisez des colonies fraîches.
Lysinibacillus fusiformis DSM 2898T BRB	La qualité des spectres (qui influe sur le score) dépend du degré de sporulation: Utilisez des colonies fraîches.
Lysinibacillus fusiformis DSM 2898T DSM	La qualité des spectres (qui influe sur le score) dépend du degré de sporulation: Utilisez des colonies fraîches.
Indication de référence --suite page suivante	

Indication de référence -- suite de la page précédente	
**Profil de référence**	**Commentaire**
Lysinibacillus fusiformis DSM 493 DSM	La qualité des spectres (qui influe sur le score) dépend du degré de sporulation: Utilisez des colonies fraîches.
Lysinibacillus sphaericus DSM 2899 DSM	La qualité des spectres (qui influe sur le score) dépend du degré de sporulation: Utilisez des colonies fraîches.
Lysinibacillus xylanilyticus CICC 20858 CICC	Les espèces boronitolerans / xylanilyticus du genre Lysinibacillus présentent des profils très similaires : il est donc difficile de distinguer ces espèces. La qualité des spectres (qui influe sur le score) dépend du degré de sporulation: Utilisez des colonies fraîches.
Proteus hauseri CC_2400 MCW	Les espèces hauseri / penneri / vulgaris du genre Proteus présentent des profils très similaires : il est donc difficile de distinguer ces espèces.
Proteus hauseri CC_2695 MCW	Les espèces hauseri / penneri / vulgaris du genre Proteus présentent des profils très similaires : il est donc difficile de distinguer ces espèces.
Proteus hauseri NY_1346 MCW	Les espèces hauseri / penneri / vulgaris du genre Proteus présentent des profils très similaires : il est donc difficile de distinguer ces espèces.
Proteus hauseri NY_1373 MCW	Les espèces hauseri / penneri / vulgaris du genre Proteus présentent des profils très similaires : il est donc difficile de distinguer ces espèces.
Proteus penneri DSM 4544T DSM	Les espèces hauseri / penneri / vulgaris du genre Proteus présentent des profils très similaires : il est donc difficile de distinguer ces espèces.
Proteus vulgaris (PX) 22086129 MLD	Les espèces hauseri / penneri / vulgaris du genre Proteus présentent des profils très similaires : il est donc difficile de distinguer ces espèces.
Proteus vulgaris DSM 13387_QC DSM	Les espèces hauseri / penneri / vulgaris du genre Proteus présentent des profils très similaires : il est donc difficile de distinguer ces espèces.
Proteus vulgaris DSM 13625 DSM	Les espèces hauseri / penneri / vulgaris du genre Proteus présentent des profils très similaires : il est donc difficile de distinguer ces espèces.
Proteus vulgaris DSM 30119 DSM	Les espèces hauseri / penneri / vulgaris du genre Proteus présentent des profils très similaires : il est donc difficile de distinguer ces espèces.
Proteus vulgaris DSM 46228 DSM	Les espèces hauseri / penneri / vulgaris du genre Proteus présentent des profils très similaires : il est donc difficile de distinguer ces espèces.
Proteus vulgaris LMG 5586 LMG	Les espèces hauseri / penneri / vulgaris du genre Proteus présentent des profils très similaires : il est donc difficile de distinguer ces espèces.
Pseudomonas cedrina ssp cedrina CIP 105541T HAM	appartient au groupe Pseudomonas fluorescens
Pseudomonas monteilii DSM 14164T HAM	appartient au groupe Pseudomonas putida
Indication de référence --suite page suivante	

Indication de référence -- suite de la page précédente	
**Profil de référence**	**Commentaire**
Raoultella ornithinolytica MB_18887 CHB	Les espèces Klebsiella oxytoca et ornithinolytica / planticola / terrigena du genre Raoultella présentent des profils très similaires : il est donc difficile de distinguer ces espèces.
Salmonella sp (enterica st Anatum) 11 LAL	Les Salmonelles peuvent seulement être identifiées au niveau du genre.
Solibacillus silvestris DSM 12223T DSM	La qualité des spectres (qui influe sur le score) dépend du degré de sporulation: Utilisez des colonies fraîches.

Correspondances des scores

Valeur	Description	Symboles	Couleur
2.00 - 3.00	Identification avec une haute confiance	(+++)	vert
1.70 - 1.99	Identification avec une faible confiance	(+)	jaune
0.00 - 1.69	Aucune identification possible	(-)	rouge

Signification des indices de cohérence (A - C)

Catégorie	Description
(A)	**Haute cohérence :** Le meilleur résultat correspond à une identification avec une haute confiance. Le deuxième meilleur résultat est (1) une identification avec une haute confiance, dans laquelle l'espèce est identique au meilleur résultat, (2) une identification avec une faible confiance, dans laquelle le genre est identique au meilleur résultat, ou (3) une absence d'identification.
(B)	**Faible cohérence :** Les exigences pour un indice de cohérence élevé ne sont pas atteintes. Le meilleur résultat correspond à une identification avec une confiance haute ou faible. Le deuxième meilleur résultat est (1) une identification avec une confiance haute ou faible, dans laquelle le genre est identique au meilleur résultat ou (2) une absence d'identification.
(C)	**Pas de cohérence :** Les exigences pour un indice de cohérence élevé ou faible ne sont pas atteintes.

Analyte 1

**Nom de l'échantillon:**	A1
**Description de l'échantillon:**	
**ID de l'échantillon:**	A1
**Date/Heure de création de l'échantillon:**	2019-07-10T18:42:16.569
**Type de l'échantillon:**	Échantillon standard
**Méthode de classification :**	MALDI Biotyper MSP Identification Standard Method 1.1
**Méthode de prétraitement :**	BioTyper Preprocessing Standard Method 1.2
**Méthode ACQ :**	D:\Methods\flexControlMethods\MBT_FC.par
**Horodatage ACQ :**	2019-07-10T18:42:37.787
**Méthode AutoXecute :**	MBT_AutoX_smart
**Bibliothèque de MSP utilisée:**	BDAL / contains 7854 MSPs / e7ef41ca-b750-4d47-9a1c-6c26fa454356 / 2019-01-02T15:31:15.698, Timone / de47ac8d-677c-4f70-821a-4bf7f4ccfa8d / 2019-01-22T15:40:50.648, Culturomics / 89878d5c-559e-4a65-96a9-6a526c01a7ee / 2019-06-28T13:49:07.074

Classement (Qualité)	Profil de référence	Score Valeur	Identifiant NCBI
1 (+++)	Bacillus pumilus CSURP4226	2.32	131944301
2 (+++)	Bacillus pumilus CSURP8100	2.25	131944301
3 (+++)	Bacillus pumilus CSURP4105	2.10	131944301
4 (+++)	Bacillus pumilus CSURP4085	2.05	131944301
5 (+++)	Bacillus pumilus 10403987	2.03	147143335
6 (+++)	Bacillus pumilus CSURP6343	2.00	131944301
7 (+++)	Bacillus pumilus CSURP6343	2.00	131944301
8 (+)	Bacillus pumilus 10403329	1.94	147143335
9 (+)	Bacillus pumilus 10403607	1.87	147143335

Tableau des résultats pour analyte 1--suite page suivante

Tableau des résultats pour analyte 1 -- suite de la page précédente			
**Classement (Qualité)**	**Profil de référence**	**Score Valeur**	**Identifiant NCBI**
10 (+)	Bacillus pumilus CSURP505	1.86	131944301

Analyte 2

**Nom de l'échantillon:**	A2
**Description de l'échantillon:**	
**ID de l'échantillon:**	A2
**Date/Heure de création de l'échantillon:**	2019-07-10T18:42:16.570
**Type de l'échantillon:**	Échantillon standard
**Méthode de classification :**	MALDI Biotyper MSP Identification Standard Method 1.1
**Méthode de prétraitement :**	BioTyper Preprocessing Standard Method 1.2
**Méthode ACQ :**	D:\Methods\flexControlMethods\MBT_FC.par
**Horodatage ACQ :**	2019-07-10T18:43:02.058
**Méthode AutoXecute :**	MBT_AutoX_smart
**Bibliothèque de MSP utilisée:**	BDAL / contains 7854 MSPs / e7ef41ca-b750-4d47-9a1c-6c26fa454356 / 2019-01-02T15:31:15.698, Timone / de47ac8d-677c-4f70-821a-4bf7f4ccfa8d / 2019-01-22T15:40:50.648, Culturomics / 89878d5c-559e-4a65-96a9-6a526c01a7ee / 2019-06-28T13:49:07.074

Classement (Qualité)	Profil de référence	Score Valeur	Identifiant NCBI
1 (-)	Bacillus pumilus 10403329	1.58	147143335
2 (-)	Bacillus pumilus CSURP6343	1.54	131944301
3 (-)	Bacillus pumilus CSURP6343	1.54	131944301
4 (-)	Bacillus pumilus CSURP4085	1.50	131944301
5 (-)	Bacillus pumilus CSURP4105	1.41	131944301
6 (-)	Bacillus pumilus CSURP8100	1.35	131944301
7 (-)	Bacillus pumilus 10403206	1.33	147143335
8 (-)	Clostridium sporogenes CSURP3354	1.28	131944301
9 (-)	Bacillus pumilus 10403990	1.28	147143335

Tableau des résultats pour analyte 2--suite page suivante

Tableau des résultats pour analyte 2 -- suite de la page précédente			
**Classement (Qualité)**	**Profil de référence**	**Score Valeur**	**Identifiant NCBI**
10<br>(-)	Bacillus pumilus 10403987	1.25	147143335

Analyte 3

Nom de l'échantillon: A3
Description de l'échantillon:
ID de l'échantillon: A3
Date/Heure de création de l'échantillon: 2019-07-10T18:42:16.572
Type de l'échantillon: Échantillon standard
Méthode de classification : MALDI Biotyper MSP Identification Standard Method 1.1
Méthode de prétraitement : BioTyper Preprocessing Standard Method 1.2
Méthode ACQ : D:\Methods\flexControlMethods\MBT_FC.par
Horodatage ACQ : 2019-07-10T18:43:46.313
Méthode AutoXecute : MBT_AutoX_smart
Bibliothèque de MSP utilisée: BDAL / contains 7854 MSPs / e7ef41ca-b750-4d47-9a1c-6c26fa454356 / 2019-01-02T15:31:15.698, Timone / de47ac8d-677c-4f70-821a-4bf7f4ccfa8d / 2019-01-22T15:40:50.648, Culturomics / 89878d5c-559e-4a65-96a9-6a526c01a7ee / 2019-06-28T13:49:07.074

Classement (Qualité)	Profil de référence	Score Valeur	Identifiant NCBI
1 (-)	Bacillus cereus 9052859	1.39	147143335
2 (-)	Bacillus cereus CSURP5041	1.33	131944301
3 (-)	Bacillus toyonensis CSURP5560	1.28	131944301
4 (-)	Phoenicibacter massiliensis P5887P	1.24	131944301
5 (-)	Staphyloccocus hominis 10208785	1.22	147143335
6 (-)	Legionella feeleii 9405902	1.21	147143335
7 (-)	Pseudoacidovorax intermedius 10406591	1.21	147143335
8 (-)	Bacillus cereus CSURP4232	1.16	131944301
9 (-)	Bacillus cereus DSM 31T DSM	1.11	1396

Tableau des résultats pour analyte 3--suite page suivante

Tableau des résultats pour analyte 3 -- suite de la page précédente			
**Classement (Qualité)**	**Profil de référence**	**Score Valeur**	**Identifiant NCBI**
10<br>(-)	Staphylococcus sp. CSURP6705	1.11	131944301

Analyte 4

**Nom de l'échantillon:**	A4
**Description de l'échantillon:**	
**ID de l'échantillon:**	A4
**Date/Heure de création de l'échantillon:**	2019-07-10T18:42:16.574
**Type de l'échantillon:**	Échantillon standard
**Méthode de classification :**	MALDI Biotyper MSP Identification Standard Method 1.1
**Méthode de prétraitement :**	BioTyper Preprocessing Standard Method 1.2
**Méthode ACQ :**	D:\Methods\flexControlMethods\MBT_FC.par
**Horodatage ACQ :**	2019-07-10T18:44:35.093
**Méthode AutoXecute :**	MBT_AutoX_smart
**Bibliothèque de MSP utilisée:**	BDAL / contains 7854 MSPs / e7ef41ca-b750-4d47-9a1c-6c26fa454356 / 2019-01-02T15:31:15.698, Timone / de47ac8d-677c-4f70-821a-4bf7f4ccfa8d / 2019-01-22T15:40:50.648, Culturomics / 89878d5c-559e-4a65-96a9-6a526c01a7ee / 2019-06-28T13:49:07.074

Classement (Qualité)	Profil de référence	Score Valeur	Identifiant NCBI
1 (-)	Sphingobium chlorophenolicum DSM 7098T HAM	1.35	46429
2 (-)	Bacillus cereus CSURP5041	1.31	131944301
3 (-)	Pseudomonas monteilii DSM 14164T HAM	1.31	76759
4 (-)	Bacteroides fragilis MB_5088_05 THL	1.26	817
5 (-)	Acidovorax temperans DSM 7270T HAM	1.25	80878
6 (-)	Pandoraea norimbergensis DSM 11628T HAM	1.25	93219
7 (-)	Bacillus cereus DSM 31T DSM	1.25	1396
8 (-)	Acinetobacter tandoii DSM 14970T HAM	1.24	202954
9 (-)	Staphylococcus hominis 18 ESL	1.24	1290

Tableau des résultats pour analyte 4--suite page suivante

Tableau des résultats pour analyte 4 -- suite de la page précédente			
**Classement (Qualité)**	**Profil de référence**	**Score Valeur**	**Identifiant NCBI**
10<br>(-)	Bacillus weihenstephanensis DSM 11821T DSM	1.23	86662

Analyte 5

**Nom de l'échantillon:**	A5
**Description de l'échantillon:**	
**ID de l'échantillon:**	A5
**Date/Heure de création de l'échantillon:**	2019-07-10T18:42:16.576
**Type de l'échantillon:**	Échantillon standard
**Méthode de classification :**	MALDI Biotyper MSP Identification Standard Method 1.1
**Méthode de prétraitement :**	BioTyper Preprocessing Standard Method 1.2
**Méthode ACQ :**	D:\Methods\flexControlMethods\MBT_FC.par
**Horodatage ACQ :**	2019-07-10T18:44:58.688
**Méthode AutoXecute :**	MBT_AutoX_smart
**Bibliothèque de MSP utilisée:**	BDAL / contains 7854 MSPs / e7ef41ca-b750-4d47-9a1c-6c26fa454356 / 2019-01-02T15:31:15.698, Timone / de47ac8d-677c-4f70-821a-4bf7f4ccfa8d / 2019-01-22T15:40:50.648, Culturomics / 89878d5c-559e-4a65-96a9-6a526c01a7ee / 2019-06-28T13:49:07.074

Classement (Qualité)	Profil de référence	Score Valeur	Identifiant NCBI
1 (-)	Tatumella citrea DSM 13699T HAM	1.15	53336
2 (-)	Klebsiella oxytoca ATCC 700324 THL	1.13	571
3 (-)	Arthrobacter luteolus DSM 13067T DSM	1.12	98672
4 (-)	Mycobacterium smegmatis 19 PGM	1.11	1772
5 (-)	Escherichia coli MB11464_1 CHB	1.09	562
6 (-)	Megamonas sp[2] 07_136 N2 IBS	1.07	158846
7 (-)	Escherichia coli ATCC 35218 CHB	1.06	562
8 (-)	Lactobacillus delbrueckii ssp lactis DSM 20072T DSM	1.04	29397
9 (-)	Eubacterium limosum 11 RLT	1.04	1736

Tableau des résultats pour analyte 5--suite page suivante

Tableau des résultats pour analyte 5 -- suite de la page précédente			
**Classement (Qualité)**	**Profil de référence**	**Score Valeur**	**Identifiant NCBI**
10 (-)	Bacillus pumilus 10403329	1.03	147143335

Analyte 6

**Nom de l'échantillon:**	A6
**Description de l'échantillon:**	
**ID de l'échantillon:**	A6
**Date/Heure de création de l'échantillon:**	2019-07-10T18:42:16.578
**Type de l'échantillon:**	Échantillon standard
**Méthode de classification :**	MALDI Biotyper MSP Identification Standard Method 1.1
**Méthode de prétraitement :**	BioTyper Preprocessing Standard Method 1.2
**Méthode ACQ :**	D:\Methods\flexControlMethods\MBT_FC.par
**Horodatage ACQ :**	2019-07-10T18:45:17.806
**Méthode AutoXecute :**	MBT_AutoX_smart
**Bibliothèque de MSP utilisée:**	BDAL / contains 7854 MSPs / e7ef41ca-b750-4d47-9a1c-6c26fa454356 / 2019-01-02T15:31:15.698, Timone / de47ac8d-677c-4f70-821a-4bf7f4ccfa8d / 2019-01-22T15:40:50.648, Culturomics / 89878d5c-559e-4a65-96a9-6a526c01a7ee / 2019-06-28T13:49:07.074

Classement (Qualité)	Profil de référence	Score Valeur	Identifiant NCBI
1 (+++)	Bacillus pumilus CSURP4085	2.02	131944301
2 (+)	Bacillus pumilus CSURP8100	1.97	131944301
3 (+)	Bacillus pumilus 10403329	1.93	147143335
4 (+)	Bacillus pumilus CSURP4226	1.87	131944301
5 (+)	Bacillus pumilus 10403607	1.80	147143335
6 (+)	Bacillus pumilus CSURP4105	1.78	131944301
7 (+)	Bacillus pumilus 10403987	1.75	147143335
8 (+)	Bacillus pumilus 10403206	1.75	147143335
9 (-)	Bacillus pumilus 10403990	1.67	147143335

Tableau des résultats pour analyte 6--suite page suivante

Tableau des résultats pour analyte 6 -- suite de la page précédente			
**Classement (Qualité)**	**Profil de référence**	**Score Valeur**	**Identifiant NCBI**
10<br>(-)	Bacillus pumilus CSURP6343	1.64	131944301

Analyte 7

**Nom de l’échantillon:**	A7
**Description de l’échantillon:**	
**ID de l’échantillon:**	A7
**Date/Heure de création de l’échantillon:**	2019-07-10T18:42:16.579
**Type de l’échantillon:**	Échantillon standard
**Méthode de classification :**	MALDI Biotyper MSP Identification Standard Method 1.1
**Méthode de prétraitement :**	BioTyper Preprocessing Standard Method 1.2
**Méthode ACQ :**	D:\Methods\flexControlMethods\MBT_FC.par
**Horodatage ACQ :**	2019-07-10T18:45:38.504
**Méthode AutoXecute :**	MBT_AutoX_smart
**Bibliothèque de MSP utilisée:**	BDAL / contains 7854 MSPs / e7ef41ca-b750-4d47-9a1c-6c26fa454356 / 2019-01-02T15:31:15.698, Timone / de47ac8d-677c-4f70-821a-4bf7f4ccfa8d / 2019-01-22T15:40:50.648, Culturomics / 89878d5c-559e-4a65-96a9-6a526c01a7ee / 2019-06-28T13:49:07.074

Classement (Qualité)	Profil de référence	Score Valeur	Identifiant NCBI
1 (+++)	Bacillus pumilus CSURP4226	2.20	131944301
2 (+++)	Bacillus pumilus CSURP8100	2.17	131944301
3 (+++)	Bacillus pumilus CSURP6343	2.13	131944301
4 (+++)	Bacillus pumilus CSURP6343	2.13	131944301
5 (+++)	Bacillus pumilus 10403329	2.02	147143335
6 (+)	Bacillus pumilus CSURP4085	1.95	131944301
7 (+)	Bacillus pumilus 10403987	1.88	147143335
8 (+)	Bacillus pumilus 10149151	1.87	147143335
9 (+)	Bacillus pumilus 10403607	1.81	147143335

Tableau des résultats pour analyte 7--suite page suivante

Tableau des résultats pour analyte 7 -- suite de la page précédente			
**Classement (Qualité)**	**Profil de référence**	**Score Valeur**	**Identifiant NCBI**
10<br>(+)	Bacillus pumilus 10403985	1.77	147143335

Analyte 8

**Nom de l'échantillon:**	A8
**Description de l'échantillon:**	
**ID de l'échantillon:**	A8
**Date/Heure de création de l'échantillon:**	2019-07-10T18:42:16.580
**Type de l'échantillon:**	Échantillon standard
**Méthode de classification :**	MALDI Biotyper MSP Identification Standard Method 1.1
**Méthode de prétraitement :**	BioTyper Preprocessing Standard Method 1.2
**Méthode ACQ :**	D:\Methods\flexControlMethods\MBT_FC.par
**Horodatage ACQ :**	2019-07-10T18:45:59.837
**Méthode AutoXecute :**	MBT_AutoX_smart
**Bibliothèque de MSP utilisée:**	BDAL / contains 7854 MSPs / e7ef41ca-b750-4d47-9a1c-6c26fa454356 / 2019-01-02T15:31:15.698, Timone / de47ac8d-677c-4f70-821a-4bf7f4ccfa8d / 2019-01-22T15:40:50.648, Culturomics / 89878d5c-559e-4a65-96a9-6a526c01a7ee / 2019-06-28T13:49:07.074

Classement (Qualité)	Profil de référence	Score Valeur	Identifiant NCBI
1 (+++)	Bacillus pumilus CSURP6343	2.20	131944301
2 (+++)	Bacillus pumilus CSURP6343	2.20	131944301
3 (+++)	Bacillus pumilus CSURP4105	2.17	131944301
4 (+++)	Bacillus pumilus CSURP4226	2.14	131944301
5 (+++)	Bacillus pumilus CSURP8100	2.08	131944301
6 (+)	Bacillus pumilus CSURP4085	1.96	131944301
7 (+)	Bacillus pumilus 10403329	1.88	147143335
8 (+)	Bacillus pumilus 10149151	1.87	147143335
9 (+)	Bacillus pumilus 10403607	1.87	147143335

Tableau des résultats pour analyte 8--suite page suivante

Tableau des résultats pour analyte 8 -- suite de la page précédente			
**Classement (Qualité)**	**Profil de référence**	**Score Valeur**	**Identifiant NCBI**
10<br>(+)	Bacillus pumilus 10403987	1.82	147143335

Analyte 9

**Nom de l'échantillon:**	A9
**Description de l'échantillon:**	
**ID de l'échantillon:**	A9
**Date/Heure de création de l'échantillon:**	2019-07-10T18:42:16.582
**Type de l'échantillon:**	Échantillon standard
**Méthode de classification :**	MALDI Biotyper MSP Identification Standard Method 1.1
**Méthode de prétraitement :**	BioTyper Preprocessing Standard Method 1.2
**Méthode ACQ :**	D:\Methods\flexControlMethods\MBT_FC.par
**Horodatage ACQ :**	2019-07-10T18:46:20.740
**Méthode AutoXecute :**	MBT_AutoX_smart
**Bibliothèque de MSP utilisée:**	BDAL / contains 7854 MSPs / e7ef41ca-b750-4d47-9a1c-6c26fa454356 / 2019-01-02T15:31:15.698, Timone / de47ac8d-677c-4f70-821a-4bf7f4ccfa8d / 2019-01-22T15:40:50.648, Culturomics / 89878d5c-559e-4a65-96a9-6a526c01a7ee / 2019-06-28T13:49:07.074

Classement (Qualité)	Profil de référence	Score Valeur	Identifiant NCBI
1 (+++)	Bacillus pumilus CSURP4105	2.11	131944301
2 (+++)	Bacillus pumilus CSURP8100	2.08	131944301
3 (+++)	Bacillus pumilus CSURP6343	2.08	131944301
4 (+++)	Bacillus pumilus CSURP6343	2.08	131944301
5 (+)	Bacillus pumilus CSURP4226	1.95	131944301
6 (+)	Bacillus pumilus 10403329	1.95	147143335
7 (+)	Bacillus pumilus DSM 354 DSM	1.78	1408
8 (+)	Bacillus pumilus CSURP4085	1.72	131944301
9 (-)	Bacillus pumilus 10149151	1.63	147143335

Tableau des résultats pour analyte 9--suite page suivante

Tableau des résultats pour analyte 9 -- suite de la page précédente			
**Classement (Qualité)**	**Profil de référence**	**Score Valeur**	**Identifiant NCBI**
10<br>(-)	Bacillus pumilus CSURP8210	1.62	131944301

Analyte 10

**Nom de l'échantillon:**	A10
**Description de l'échantillon:**	
**ID de l'échantillon:**	A10
**Date/Heure de création de l'échantillon:**	2019-07-10T18:42:16.584
**Type de l'échantillon:**	Échantillon standard
**Méthode de classification :**	MALDI Biotyper MSP Identification Standard Method 1.1
**Méthode de prétraitement :**	BioTyper Preprocessing Standard Method 1.2
**Méthode ACQ :**	D:\Methods\flexControlMethods\MBT_FC.par
**Horodatage ACQ :**	2019-07-10T18:46:42.274
**Méthode AutoXecute :**	MBT_AutoX_smart
**Bibliothèque de MSP utilisée:**	BDAL / contains 7854 MSPs / e7ef41ca-b750-4d47-9a1c-6c26fa454356 / 2019-01-02T15:31:15.698, Timone / de47ac8d-677c-4f70-821a-4bf7f4ccfa8d / 2019-01-22T15:40:50.648, Culturomics / 89878d5c-559e-4a65-96a9-6a526c01a7ee / 2019-06-28T13:49:07.074

Classement (Qualité)	Profil de référence	Score Valeur	Identifiant NCBI
1 (+++)	Bacillus pumilus CSURP4085	2.06	131944301
2 (+)	Bacillus pumilus CSURP8100	1.88	131944301
3 (+)	Bacillus pumilus 10403329	1.88	147143335
4 (+)	Bacillus pumilus 10403206	1.88	147143335
5 (+)	Bacillus pumilus CSURP4226	1.74	131944301
6 (+)	Bacillus pumilus 10403607	1.74	147143335
7 (+)	Bacillus pumilus CSURP4105	1.72	131944301
8 (+)	Bacillus pumilus 10403987	1.70	147143335
9 (-)	Bacillus pumilus 10403990	1.66	147143335

Tableau des résultats pour analyte 10--suite page suivante

Tableau des résultats pour analyte 10 -- suite de la page précédente			
**Classement (Qualité)**	**Profil de référence**	**Score Valeur**	**Identifiant NCBI**
10<br>(-)	Bacillus pumilus CSURP6343	1.52	131944301

Analyte 11

**Nom de l'échantillon:**	A11
**Description de l'échantillon:**	
**ID de l'échantillon:**	A11
**Date/Heure de création de l'échantillon:**	2019-07-10T18:42:16.585
**Type de l'échantillon:**	Échantillon standard
**Méthode de classification :**	MALDI Biotyper MSP Identification Standard Method 1.1
**Méthode de prétraitement :**	BioTyper Preprocessing Standard Method 1.2
**Méthode ACQ :**	D:\Methods\flexControlMethods\MBT_FC.par
**Horodatage ACQ :**	2019-07-10T18:47:04.241
**Méthode AutoXecute :**	MBT_AutoX_smart
**Bibliothèque de MSP utilisée:**	BDAL / contains 7854 MSPs / e7ef41ca-b750-4d47-9a1c-6c26fa454356 / 2019-01-02T15:31:15.698, Timone / de47ac8d-677c-4f70-821a-4bf7f4ccfa8d / 2019-01-22T15:40:50.648, Culturomics / 89878d5c-559e-4a65-96a9-6a526c01a7ee / 2019-06-28T13:49:07.074

Classement (Qualité)	Profil de référence	Score Valeur	Identifiant NCBI
1 (+++)	Bacillus pumilus CSURP4226	2.61	131944301
2 (+++)	Bacillus pumilus CSURP8100	2.49	131944301
3 (+++)	Bacillus pumilus CSURP4105	2.21	131944301
4 (+++)	Bacillus pumilus CSURP4085	2.20	131944301
5 (+++)	Bacillus pumilus 10403607	2.07	147143335
6 (+++)	Bacillus pumilus CSURP3862	2.05	131944301
7 (+++)	Bacillus pumilus 10403329	2.00	147143335
8 (+)	Bacillus pumilus 10403987	1.91	147143335
9 (+)	Bacillus pumilus CSURP6343	1.90	131944301

Tableau des résultats pour analyte 11--suite page suivante

Tableau des résultats pour analyte 11 -- suite de la page précédente			
**Classement (Qualité)**	**Profil de référence**	**Score Valeur**	**Identifiant NCBI**
10<br>(+)	Bacillus pumilus CSURP6343	1.90	131944301

Analyte 12

Nom de l'échantillon: A12
Description de l'échantillon:
ID de l'échantillon: A12
Date/Heure de création de l'échantillon: 2019-07-10T18:42:16.586
Type de l'échantillon: Échantillon standard
Méthode de classification : MALDI Biotyper MSP Identification Standard Method 1.1
Méthode de prétraitement : BioTyper Preprocessing Standard Method 1.2
Méthode ACQ : D:\Methods\flexControlMethods\MBT_FC.par
Horodatage ACQ : 2019-07-10T18:47:22.150
Méthode AutoXecute : MBT_AutoX_smart
Bibliothèque de MSP utilisée: BDAL / contains 7854 MSPs / e7ef41ca-b750-4d47-9a1c-6c26fa454356 / 2019-01-02T15:31:15.698, Timone / de47ac8d-677c-4f70-821a-4bf7f4ccfa8d / 2019-01-22T15:40:50.648, Culturomics / 89878d5c-559e-4a65-96a9-6a526c01a7ee / 2019-06-28T13:49:07.074

Classement (Qualité)	Profil de référence	Score Valeur	Identifiant NCBI
1 (+++)	Bacillus pumilus CSURP4226	2.16	131944301
2 (+)	Bacillus pumilus CSURP4085	1.99	131944301
3 (+)	Bacillus pumilus 10403987	1.98	147143335
4 (+)	Bacillus pumilus CSURP8100	1.98	131944301
5 (+)	Bacillus pumilus 10403329	1.92	147143335
6 (+)	Bacillus pumilus 10403985	1.83	147143335
7 (+)	Bacillus pumilus 10403607	1.80	147143335
8 (+)	Bacillus pumilus 10403990	1.77	147143335
9 (+)	Bacillus pumilus 10403751	1.75	147143335

Tableau des résultats pour analyte 12--suite page suivante

Tableau des résultats pour analyte 12 -- suite de la page précédente			
**Classement (Qualité)**	**Profil de référence**	**Score Valeur**	**Identifiant NCBI**
10<br>(-)	Bacillus pumilus CSURP4105	1.67	131944301

Analyte 13

**Nom de l'échantillon:**	B1
**Description de l'échantillon:**	
**ID de l'échantillon:**	B1
**Date/Heure de création de l'échantillon:**	2019-07-10T18:42:16.588
**Type de l'échantillon:**	Échantillon standard
**Méthode de classification :**	MALDI Biotyper MSP Identification Standard Method 1.1
**Méthode de prétraitement :**	BioTyper Preprocessing Standard Method 1.2
**Méthode ACQ :**	D:\Methods\flexControlMethods\MBT_FC.par
**Horodatage ACQ :**	2019-07-10T18:47:42.794
**Méthode AutoXecute :**	MBT_AutoX_smart
**Bibliothèque de MSP utilisée:**	BDAL / contains 7854 MSPs / e7ef41ca-b750-4d47-9a1c-6c26fa454356 / 2019-01-02T15:31:15.698, Timone / de47ac8d-677c-4f70-821a-4bf7f4ccfa8d / 2019-01-22T15:40:50.648, Culturomics / 89878d5c-559e-4a65-96a9-6a526c01a7ee / 2019-06-28T13:49:07.074

Classement (Qualité)	Profil de référence	Score Valeur	Identifiant NCBI
1 (+++)	Bacillus subtilis CSURP291	2.35	131944301
2 (+++)	Bacillus subtilis ssp subtilis DSM 5660 DSM	2.05	135461
3 (+++)	Lactobacillus fermentum CSURP4362	2.03	131944301
4 (+++)	Bacillus subtilis DSM 5611 DSM	2.02	1423
5 (+)	Bacillus subtilis ssp subtilis DSM 10T DSM	1.92	135461
6 (+)	Bacillus vallismortis CSURP984	1.90	131944301
7 (+)	Bacillus subtilis CSURP3865	1.89	131944301
8 (+)	Bacillus subtilis DSM 5552 DSM	1.87	1423
9 (+)	Bacillus mojavensis DSM 9205T DSM	1.75	72360

Tableau des résultats pour analyte 13--suite page suivante

Tableau des résultats pour analyte 13 -- suite de la page précédente			
**Classement (Qualité)**	**Profil de référence**	**Score Valeur**	**Identifiant NCBI**
10<br>(-)	Bacillus mojavensis CSURP1524	1.66	131944301

Analyte 14

**Nom de l'échantillon:**	B2
**Description de l'échantillon:**	
**ID de l'échantillon:**	B2
**Date/Heure de création de l'échantillon:**	2019-07-10T18:42:16.590
**Type de l'échantillon:**	Échantillon standard
**Méthode de classification :**	MALDI Biotyper MSP Identification Standard Method 1.1
**Méthode de prétraitement :**	BioTyper Preprocessing Standard Method 1.2
**Méthode ACQ :**	D:\Methods\flexControlMethods\MBT_FC.par
**Horodatage ACQ :**	2019-07-10T18:48:03.526
**Méthode AutoXecute :**	MBT_AutoX_smart
**Bibliothèque de MSP utilisée:**	BDAL / contains 7854 MSPs / e7ef41ca-b750-4d47-9a1c-6c26fa454356 / 2019-01-02T15:31:15.698, Timone / de47ac8d-677c-4f70-821a-4bf7f4ccfa8d / 2019-01-22T15:40:50.648, Culturomics / 89878d5c-559e-4a65-96a9-6a526c01a7ee / 2019-06-28T13:49:07.074

Classement (Qualité)	Profil de référence	Score Valeur	Identifiant NCBI
1 (+++)	Lactobacillus fermentum CSURP4362	2.15	131944301
2 (+++)	Bacillus subtilis CSURP291	2.14	131944301
3 (+)	Bacillus vallismortis CSURP984	1.87	131944301
4 (+)	Bacillus subtilis DSM 5611 DSM	1.87	1423
5 (+)	Bacillus subtilis ssp subtilis DSM 5660 DSM	1.85	135461
6 (+)	Bacillus subtilis CSURP3865	1.83	131944301
7 (+)	Bacillus subtilis ssp subtilis DSM 10T DSM	1.72	135461
8 (+)	Bacillus subtilis DSM 5552 DSM	1.72	1423
9 (-)	Bacillus mojavensis DSM 9205T DSM	1.66	72360

Tableau des résultats pour analyte 14--suite page suivante

Tableau des résultats pour analyte 14 -- suite de la page précédente			
**Classement (Qualité)**	**Profil de référence**	**Score Valeur**	**Identifiant NCBI**
10<br>(-)	Bacillus mojavensis CSURP1524	1.58	131944301

Analyte 15

**Nom de l'échantillon:**	B3
**Description de l'échantillon:**	
**ID de l'échantillon:**	B3
**Date/Heure de création de l'échantillon:**	2019-07-10T18:42:16.592
**Type de l'échantillon:**	Échantillon standard
**Méthode de classification :**	MALDI Biotyper MSP Identification Standard Method 1.1
**Méthode de prétraitement :**	BioTyper Preprocessing Standard Method 1.2
**Méthode ACQ :**	D:\Methods\flexControlMethods\MBT_FC.par
**Horodatage ACQ :**	2019-07-10T18:48:24.881
**Méthode AutoXecute :**	MBT_AutoX_smart
**Bibliothèque de MSP utilisée:**	BDAL / contains 7854 MSPs / e7ef41ca-b750-4d47-9a1c-6c26fa454356 / 2019-01-02T15:31:15.698, Timone / de47ac8d-677c-4f70-821a-4bf7f4ccfa8d / 2019-01-22T15:40:50.648, Culturomics / 89878d5c-559e-4a65-96a9-6a526c01a7ee / 2019-06-28T13:49:07.074

Classement (Qualité)	Profil de référence	Score Valeur	Identifiant NCBI
1 (+)	Bacillus pumilus CSURP4085	1.99	131944301
2 (+)	Bacillus pumilus 10403329	1.84	147143335
3 (+)	Bacillus pumilus 10403206	1.77	147143335
4 (+)	Bacillus pumilus CSURP4105	1.70	131944301
5 (-)	Bacillus pumilus CSURP4226	1.63	131944301
6 (-)	Bacillus pumilus CSURP8100	1.62	131944301
7 (-)	Bacillus pumilus 10403990	1.53	147143335
8 (-)	Bacillus pumilus 10149151	1.44	147143335
9 (-)	Bacillus pumilus CSURP505	1.43	131944301

Tableau des résultats pour analyte 15--suite page suivante

Tableau des résultats pour analyte 15 -- suite de la page précédente			
**Classement (Qualité)**	**Profil de référence**	**Score Valeur**	**Identifiant NCBI**
10<br>(-)	Bacillus pumilus 10403985	1.36	147143335

Analyte 16

**Nom de l'échantillon:**	B4
**Description de l'échantillon:**	
**ID de l'échantillon:**	B4
**Date/Heure de création de l'échantillon:**	2019-07-10T18:42:16.594
**Type de l'échantillon:**	Échantillon standard
**Méthode de classification :**	MALDI Biotyper MSP Identification Standard Method 1.1
**Méthode de prétraitement :**	BioTyper Preprocessing Standard Method 1.2
**Méthode ACQ :**	D:\Methods\flexControlMethods\MBT_FC.par
**Horodatage ACQ :**	2019-07-10T18:48:42.192
**Méthode AutoXecute :**	MBT_AutoX_smart
**Bibliothèque de MSP utilisée:**	BDAL / contains 7854 MSPs / e7ef41ca-b750-4d47-9a1c-6c26fa454356 / 2019-01-02T15:31:15.698, Timone / de47ac8d-677c-4f70-821a-4bf7f4ccfa8d / 2019-01-22T15:40:50.648, Culturomics / 89878d5c-559e-4a65-96a9-6a526c01a7ee / 2019-06-28T13:49:07.074

Classement (Qualité)	Profil de référence	Score Valeur	Identifiant NCBI
1 (+++)	Bacillus pumilus CSURP8100	2.47	131944301
2 (+++)	Bacillus pumilus CSURP4226	2.47	131944301
3 (+++)	Bacillus pumilus CSURP4105	2.26	131944301
4 (+++)	Bacillus pumilus CSURP4085	2.18	131944301
5 (+++)	Bacillus pumilus 10403329	2.16	147143335
6 (+++)	Bacillus pumilus 10403607	2.08	147143335
7 (+++)	Bacillus pumilus 10403987	2.07	147143335
8 (+++)	Bacillus pumilus CSURP6343	2.06	131944301
9 (+++)	Bacillus pumilus CSURP6343	2.06	131944301

Tableau des résultats pour analyte 16--suite page suivante

Tableau des résultats pour analyte 16 -- suite de la page précédente			
**Classement (Qualité)**	**Profil de référence**	**Score Valeur**	**Identifiant NCBI**
10<br>(+)	Bacillus pumilus 10149151	1.94	147143335

Analyte 17

**Nom de l'échantillon:**	B5
**Description de l'échantillon:**	
**ID de l'échantillon:**	B5
**Date/Heure de création de l'échantillon:**	2019-07-10T18:42:16.596
**Type de l'échantillon:**	Échantillon standard
**Méthode de classification :**	MALDI Biotyper MSP Identification Standard Method 1.1
**Méthode de prétraitement :**	BioTyper Preprocessing Standard Method 1.2
**Méthode ACQ :**	D:\Methods\flexControlMethods\MBT_FC.par
**Horodatage ACQ :**	2019-07-10T18:49:01.600
**Méthode AutoXecute :**	MBT_AutoX_smart
**Bibliothèque de MSP utilisée:**	BDAL / contains 7854 MSPs / e7ef41ca-b750-4d47-9a1c-6c26fa454356 / 2019-01-02T15:31:15.698, Timone / de47ac8d-677c-4f70-821a-4bf7f4ccfa8d / 2019-01-22T15:40:50.648, Culturomics / 89878d5c-559e-4a65-96a9-6a526c01a7ee / 2019-06-28T13:49:07.074

Classement (Qualité)	Profil de référence	Score Valeur	Identifiant NCBI
1 (-)	Brevibacillus laterosporus DSM 8787 DSM	1.51	1465
2 (-)	Aeromonas schubertii CECT 4240T DSM	1.37	652
3 (-)	Paenibacillus dendritiformis DSM 18844T DSM	1.27	130049
4 (-)	Brevibacillus laterosporus DSM 25T DSM	1.23	1465
5 (-)	Gordonia rubripertincta DSM 43570 DSM	1.23	36822
6 (-)	Paenibacillus glucanolyticus DSM 5162T DSM	1.22	59843
7 (-)	Paenibacillus brasilensis DSM 14914T DSM	1.21	128574
8 (-)	Paenibacillus agaridevorans DSM 1486 DSM	1.16	171404
9 (-)	Pseudomonas syringae ssp syringae LMG 1247T HAM	1.16	317

Tableau des résultats pour analyte 17--suite page suivante

Tableau des résultats pour analyte 17 -- suite de la page précédente			
**Classement (Qualité)**	**Profil de référence**	**Score Valeur**	**Identifiant NCBI**
10<br>(-)	Solibacillus silvestris DSM 12223T DSM	1.12	76853

Analyte 18

**Nom de l'échantillon:**	B6
**Description de l'échantillon:**	
**ID de l'échantillon:**	B6
**Date/Heure de création de l'échantillon:**	2019-07-10T18:42:16.599
**Type de l'échantillon:**	Échantillon standard
**Méthode de classification :**	MALDI Biotyper MSP Identification Standard Method 1.1
**Méthode de prétraitement :**	BioTyper Preprocessing Standard Method 1.2
**Méthode ACQ :**	D:\Methods\flexControlMethods\MBT_FC.par
**Horodatage ACQ :**	2019-07-10T18:49:18.972
**Méthode AutoXecute :**	MBT_AutoX_smart
**Bibliothèque de MSP utilisée:**	BDAL / contains 7854 MSPs / e7ef41ca-b750-4d47-9a1c-6c26fa454356 / 2019-01-02T15:31:15.698, Timone / de47ac8d-677c-4f70-821a-4bf7f4ccfa8d / 2019-01-22T15:40:50.648, Culturomics / 89878d5c-559e-4a65-96a9-6a526c01a7ee / 2019-06-28T13:49:07.074

Classement (Qualité)	Profil de référence	Score Valeur	Identifiant NCBI
1 (-)	Brevibacillus laterosporus DSM 8787 DSM	1.66	1465
2 (-)	Brevibacillus laterosporus DSM 25T DSM	1.57	1465
3 (-)	Aeromonas schubertii CECT 4240T DSM	1.37	652
4 (-)	Paenibacillus agaridevorans DSM 1486 DSM	1.36	171404
5 (-)	Paenibacillus apiarius DSM 5582 DSM	1.26	46240
6 (-)	Lactobacillus concavus DSM 17758T DSM	1.26	287844
7 (-)	Burkholderia tuberum LMG 21444T HAM	1.25	157910
8 (-)	Pseudomonas brassicacearum DSM 13227T HAM	1.24	86264
9 (-)	Pseudomonas frederiksbergensis DSM 13022T HAM	1.22	104087

Tableau des résultats pour analyte 18--suite page suivante

Tableau des résultats pour analyte 18 -- suite de la page précédente			
**Classement (Qualité)**	**Profil de référence**	**Score Valeur**	**Identifiant NCBI**
10<br>(-)	Brevibacillus parabrevis DSM 8376T DSM	1.22	54914

Analyte 19

**Nom de l'échantillon:**	B7
**Description de l'échantillon:**	
**ID de l'échantillon:**	B7
**Date/Heure de création de l'échantillon:**	2019-07-10T18:42:16.601
**Type de l'échantillon:**	Échantillon standard
**Méthode de classification :**	MALDI Biotyper MSP Identification Standard Method 1.1
**Méthode de prétraitement :**	BioTyper Preprocessing Standard Method 1.2
**Méthode ACQ :**	D:\Methods\flexControlMethods\MBT_FC.par
**Horodatage ACQ :**	2019-07-10T18:49:41.622
**Méthode AutoXecute :**	MBT_AutoX_smart
**Bibliothèque de MSP utilisée:**	BDAL / contains 7854 MSPs / e7ef41ca-b750-4d47-9a1c-6c26fa454356 / 2019-01-02T15:31:15.698, Timone / de47ac8d-677c-4f70-821a-4bf7f4ccfa8d / 2019-01-22T15:40:50.648, Culturomics / 89878d5c-559e-4a65-96a9-6a526c01a7ee / 2019-06-28T13:49:07.074

Classement (Qualité)	Profil de référence	Score Valeur	Identifiant NCBI
1 (+++)	Lysinibacillus fusiformis CSURP4522	2.11	131944301
2 (+++)	Lysinibacillus fusiformis CSURP5657	2.08	131944301
3 (+++)	Lysinibacillus fusiformis CSURP2236	2.03	131944301
4 (+++)	Lysinibacillus fusiformis CSURP1153	2.03	131944301
5 (+)	Lysinibacillus fusiformis CSURP8109	1.95	131944301
6 (+)	Lysinibacillus boronitolerans DSM 17140T DSM	1.90	309788
7 (+)	Lysinibacillus fusiformis DSM 493 DSM	1.87	28031
8 (+)	Lysinibacillus xylanilyticus CICC 20858 CICC	1.85	400634
9 (+)	Lysinibacillus fusiformis DSM 2898T DSM	1.84	28031

Tableau des résultats pour analyte 19--suite page suivante

Tableau des résultats pour analyte 19 -- suite de la page précédente			
**Classement (Qualité)**	**Profil de référence**	**Score Valeur**	**Identifiant NCBI**
10<br>(+)	Lysinibacillus fusiformis DSM 2898T BRB	1.84	28031

Analyte 20

**Nom de l'échantillon:**	B8
**Description de l'échantillon:**	
**ID de l'échantillon:**	B8
**Date/Heure de création de l'échantillon:**	2019-07-10T18:42:16.603
**Type de l'échantillon:**	Échantillon standard
**Méthode de classification :**	MALDI Biotyper MSP Identification Standard Method 1.1
**Méthode de prétraitement :**	BioTyper Preprocessing Standard Method 1.2
**Méthode ACQ :**	D:\Methods\flexControlMethods\MBT_FC.par
**Horodatage ACQ :**	2019-07-10T18:50:03.567
**Méthode AutoXecute :**	MBT_AutoX_smart
**Bibliothèque de MSP utilisée:**	BDAL / contains 7854 MSPs / e7ef41ca-b750-4d47-9a1c-6c26fa454356 / 2019-01-02T15:31:15.698, Timone / de47ac8d-677c-4f70-821a-4bf7f4ccfa8d / 2019-01-22T15:40:50.648, Culturomics / 89878d5c-559e-4a65-96a9-6a526c01a7ee / 2019-06-28T13:49:07.074

Classement (Qualité)	Profil de référence	Score Valeur	Identifiant NCBI
1 (+++)	Lysinibacillus boronitolerans DSM 17140T DSM	2.16	309788
2 (+++)	Lysinibacillus fusiformis CSURP4522	2.09	131944301
3 (+++)	Lysinibacillus xylanilyticus CICC 20858 CICC	2.09	400634
4 (+++)	Lysinibacillus fusiformis CSURP2236	2.06	131944301
5 (+++)	Lysinibacillus fusiformis DSM 2898T BRB	2.03	28031
6 (+)	Lysinibacillus fusiformis DSM 493 DSM	1.99	28031
7 (+)	Lysinibacillus fusiformis CSURP5657	1.94	131944301
8 (+)	Lysinibacillus sphaericus DSM 2899 DSM	1.85	1421
9 (+)	Lysinibacillus fusiformis CSURP1153	1.81	131944301

Tableau des résultats pour analyte 20--suite page suivante

Tableau des résultats pour analyte 20 -- suite de la page précédente			
**Classement (Qualité)**	**Profil de référence**	**Score Valeur**	**Identifiant NCBI**
10 (+)	Lysinibacillus fusiformis DSM 2898T DSM	1.81	28031

Analyte 21

**Nom de l'échantillon:**	B9
**Description de l'échantillon:**	
**ID de l'échantillon:**	B9
**Date/Heure de création de l'échantillon:**	2019-07-10T18:42:16.606
**Type de l'échantillon:**	Échantillon standard
**Méthode de classification :**	MALDI Biotyper MSP Identification Standard Method 1.1
**Méthode de prétraitement :**	BioTyper Preprocessing Standard Method 1.2
**Méthode ACQ :**	D:\Methods\flexControlMethods\MBT_FC.par
**Horodatage ACQ :**	2019-07-10T18:50:25.317
**Méthode AutoXecute :**	MBT_AutoX_smart
**Bibliothèque de MSP utilisée:**	BDAL / contains 7854 MSPs / e7ef41ca-b750-4d47-9a1c-6c26fa454356 / 2019-01-02T15:31:15.698, Timone / de47ac8d-677c-4f70-821a-4bf7f4ccfa8d / 2019-01-22T15:40:50.648, Culturomics / 89878d5c-559e-4a65-96a9-6a526c01a7ee / 2019-06-28T13:49:07.074

Classement (Qualité)	Profil de référence	Score Valeur	Identifiant NCBI
1 (-)	Proteus vulgaris (PX) 22086129 MLD	1.19	585
2 (-)	Moraxella nonliquefaciens CIP 104692 CIP	1.14	478
3 (-)	Clostridium novyi A 1025_NCTC 538 BOG	1.07	1542
4 (-)	Clostridium chauvoei 1023_NCTC 8070 BOG	1.03	46867
5 (-)	Staphylococcus carnosus ssp utilis DSM 11676T DSM	1.03	147449
6 (-)	Staphylococcus carnosus ssp utilis DSM 11677 DSM	0.99	147449
7 (-)	Salmonella sp (enterica st Anatum) 11 LAL	0.98	58712
8 (-)	Acinetobacter gerneri DSM 14967T HAM	0.96	202952
9 (-)	Eggerthella timonensis CSURP3135	0.96	131944301

Tableau des résultats pour analyte 21--suite page suivante

Tableau des résultats pour analyte 21 -- suite de la page précédente			
**Classement (Qualité)**	**Profil de référence**	**Score Valeur**	**Identifiant NCBI**
10<br>(-)	Lactobacillus amylolyticus DSM 11664T DSM	0.96	83683

Analyte 22

**Nom de l'échantillon:**	B10
**Description de l'échantillon:**	
**ID de l'échantillon:**	B10
**Date/Heure de création de l'échantillon:**	2019-07-10T18:42:16.608
**Type de l'échantillon:**	Échantillon standard
**Méthode de classification :**	MALDI Biotyper MSP Identification Standard Method 1.1
**Méthode de prétraitement :**	BioTyper Preprocessing Standard Method 1.2
**Méthode ACQ :**	D:\Methods\flexControlMethods\MBT_FC.par
**Horodatage ACQ :**	2019-07-10T18:50:48.242
**Méthode AutoXecute :**	MBT_AutoX_smart
**Bibliothèque de MSP utilisée:**	BDAL / contains 7854 MSPs / e7ef41ca-b750-4d47-9a1c-6c26fa454356 / 2019-01-02T15:31:15.698, Timone / de47ac8d-677c-4f70-821a-4bf7f4ccfa8d / 2019-01-22T15:40:50.648, Culturomics / 89878d5c-559e-4a65-96a9-6a526c01a7ee / 2019-06-28T13:49:07.074

Classement (Qualité)	Profil de référence	Score Valeur	Identifiant NCBI
1 (-)	Bacillus vallismortis CSURP984	1.65	131944301
2 (-)	Bacillus pumilus 10403329	1.62	147143335
3 (-)	Bacillus mojavensis DSM 9205T DSM	1.58	72360
4 (-)	Bacillus subtilis DSM 5552 DSM	1.57	1423
5 (-)	Bacillus subtilis ssp spizizenii DSM 15029T DSM	1.56	96241
6 (-)	Bacillus subtilis CSURP291	1.49	131944301
7 (-)	Bacillus subtilis CSURP3865	1.49	131944301
8 (-)	Bacillus pumilus CSURP4226	1.42	131944301
9 (-)	Bacillus pumilus 10403607	1.42	147143335

Tableau des résultats pour analyte 22--suite page suivante

Tableau des résultats pour analyte 22 -- suite de la page précédente			
**Classement (Qualité)**	**Profil de référence**	**Score Valeur**	**Identifiant NCBI**
10 (-)	Bacillus subtilis DSM 5611 DSM	1.41	1423

Analyte 23

**Nom de l'échantillon:**	B11
**Description de l'échantillon:**	
**ID de l'échantillon:**	B11
**Date/Heure de création de l'échantillon:**	2019-07-10T18:42:16.610
**Type de l'échantillon:**	Échantillon standard
**Méthode de classification :**	MALDI Biotyper MSP Identification Standard Method 1.1
**Méthode de prétraitement :**	BioTyper Preprocessing Standard Method 1.2
**Méthode ACQ :**	D:\Methods\flexControlMethods\MBT_FC.par
**Horodatage ACQ :**	2019-07-10T18:51:03.599
**Méthode AutoXecute :**	MBT_AutoX_smart
**Bibliothèque de MSP utilisée:**	BDAL / contains 7854 MSPs / e7ef41ca-b750-4d47-9a1c-6c26fa454356 / 2019-01-02T15:31:15.698, Timone / de47ac8d-677c-4f70-821a-4bf7f4ccfa8d / 2019-01-22T15:40:50.648, Culturomics / 89878d5c-559e-4a65-96a9-6a526c01a7ee / 2019-06-28T13:49:07.074

Classement (Qualité)	Profil de référence	Score Valeur	Identifiant NCBI
1 (+++)	Bacillus pumilus CSURP4226	2.25	131944301
2 (+++)	Bacillus pumilus CSURP4085	2.00	131944301
3 (+)	Bacillus pumilus CSURP8100	1.97	131944301
4 (+)	Bacillus pumilus CSURP4105	1.86	131944301
5 (+)	Bacillus pumilus 10403329	1.82	147143335
6 (+)	Bacillus pumilus 10403607	1.81	147143335
7 (+)	Bacillus pumilus 10403987	1.79	147143335
8 (+)	Bacillus pumilus CSURP6343	1.74	131944301
9 (+)	Bacillus pumilus CSURP6343	1.74	131944301

Tableau des résultats pour analyte 23--suite page suivante

Tableau des résultats pour analyte 23 -- suite de la page précédente			
**Classement (Qualité)**	**Profil de référence**	**Score Valeur**	**Identifiant NCBI**
10<br>(+)	Bacillus pumilus 10403985	1.71	147143335

Analyte 24

**Nom de l'échantillon:**	B12
**Description de l'échantillon:**	
**ID de l'échantillon:**	B12
**Date/Heure de création de l'échantillon:**	2019-07-10T18:42:16.612
**Type de l'échantillon:**	Échantillon standard
**Méthode de classification :**	MALDI Biotyper MSP Identification Standard Method 1.1
**Méthode de prétraitement :**	BioTyper Preprocessing Standard Method 1.2
**Méthode ACQ :**	D:\Methods\flexControlMethods\MBT_FC.par
**Horodatage ACQ :**	2019-07-10T18:51:25.347
**Méthode AutoXecute :**	MBT_AutoX_smart
**Bibliothèque de MSP utilisée:**	BDAL / contains 7854 MSPs / e7ef41ca-b750-4d47-9a1c-6c26fa454356 / 2019-01-02T15:31:15.698, Timone / de47ac8d-677c-4f70-821a-4bf7f4ccfa8d / 2019-01-22T15:40:50.648, Culturomics / 89878d5c-559e-4a65-96a9-6a526c01a7ee / 2019-06-28T13:49:07.074

Classement (Qualité)	Profil de référence	Score Valeur	Identifiant NCBI
1 (+++)	Bacillus pumilus CSURP4226	2.04	131944301
2 (+++)	Bacillus pumilus CSURP4085	2.00	131944301
3 (+)	Bacillus pumilus 10403607	1.91	147143335
4 (+)	Bacillus pumilus CSURP8100	1.88	131944301
5 (+)	Bacillus pumilus CSURP4105	1.88	131944301
6 (+)	Bacillus pumilus 10403987	1.77	147143335
7 (+)	Bacillus pumilus 10403329	1.77	147143335
8 (+)	Bacillus pumilus CSURP6343	1.75	131944301
9 (+)	Bacillus pumilus CSURP6343	1.75	131944301

Tableau des résultats pour analyte 24--suite page suivante

Tableau des résultats pour analyte 24 -- suite de la page précédente			
**Classement (Qualité)**	**Profil de référence**	**Score Valeur**	**Identifiant NCBI**
10<br>(-)	Bacillus pumilus 10403990	1.68	147143335

Analyte 25

**Nom de l'échantillon:**	C1
**Description de l'échantillon:**	
**ID de l'échantillon:**	C1
**Date/Heure de création de l'échantillon:**	2019-07-10T18:42:16.613
**Type de l'échantillon:**	Échantillon standard
**Méthode de classification :**	MALDI Biotyper MSP Identification Standard Method 1.1
**Méthode de prétraitement :**	BioTyper Preprocessing Standard Method 1.2
**Méthode ACQ :**	D:\Methods\flexControlMethods\MBT_FC.par
**Horodatage ACQ :**	2019-07-10T18:51:45.706
**Méthode AutoXecute :**	MBT_AutoX_smart
**Bibliothèque de MSP utilisée:**	BDAL / contains 7854 MSPs / e7ef41ca-b750-4d47-9a1c-6c26fa454356 / 2019-01-02T15:31:15.698, Timone / de47ac8d-677c-4f70-821a-4bf7f4ccfa8d / 2019-01-22T15:40:50.648, Culturomics / 89878d5c-559e-4a65-96a9-6a526c01a7ee / 2019-06-28T13:49:07.074

Classement (Qualité)	Profil de référence	Score Valeur	Identifiant NCBI
1 (-)	Actinomyces denticolens DSM 20671T DSM	1.15	52767
2 (-)	Clostridium beijerinckii 1072_ATCC 25752T BOG	1.11	1520
3 (-)	Blautia coccoides 1035_NCTC 11035T BOG	1.08	1532
4 (-)	Phoenicibacter massiliensis P5887P	1.04	131944301
5 (-)	Arthrobacter globiformis DSM 20124T DSM	1.02	1665
6 (-)	Fusobacterium naviforme DSM 20699 BRB	0.99	77917
7 (-)	Streptobacillus moniliformis B10_9 HLG	0.98	34105
8 (-)	Clostridium clostridioforme CCUG 38271 CCUG	0.97	1531
9 (-)	Corynebacterium flavescens IMET 11080T HKJ	0.97	28028

Tableau des résultats pour analyte 25--suite page suivante

Tableau des résultats pour analyte 25 -- suite de la page précédente			
**Classement (Qualité)**	**Profil de référence**	**Score Valeur**	**Identifiant NCBI**
10<br>(-)	Aromatoleum toluolicum T MPB	0.95	12960

Analyte 26

**Nom de l'échantillon:**	C2
**Description de l'échantillon:**	
**ID de l'échantillon:**	C2
**Date/Heure de création de l'échantillon:**	2019-07-10T18:42:16.615
**Type de l'échantillon:**	Échantillon standard
**Méthode de classification :**	MALDI Biotyper MSP Identification Standard Method 1.1
**Méthode de prétraitement :**	BioTyper Preprocessing Standard Method 1.2
**Méthode ACQ :**	D:\Methods\flexControlMethods\MBT_FC.par
**Horodatage ACQ :**	2019-07-10T18:52:08.292
**Méthode AutoXecute :**	MBT_AutoX_smart
**Bibliothèque de MSP utilisée:**	BDAL / contains 7854 MSPs / e7ef41ca-b750-4d47-9a1c-6c26fa454356 / 2019-01-02T15:31:15.698, Timone / de47ac8d-677c-4f70-821a-4bf7f4ccfa8d / 2019-01-22T15:40:50.648, Culturomics / 89878d5c-559e-4a65-96a9-6a526c01a7ee / 2019-06-28T13:49:07.074

Classement (Qualité)	Profil de référence	Score Valeur	Identifiant NCBI
1 (-)	Lactobacillus agilis DSM 20510 DSM	1.19	1601
2 (-)	Kocuria rosea 10402505	1.09	147143335
3 (-)	Klebsiella pneumoniae ssp pneumoniae 9295_1 CHB	1.09	72407
4 (-)	Pseudomonas sp B538 UFL	1.08	286
5 (-)	Kytococcus sedentarius IMET 11362T HKJ	1.07	1276
6 (-)	Pseudomonas cedrina ssp cedrina CIP 105541T HAM	1.06	76762
7 (-)	Senegalobacterium massiliense P3374P	1.05	131944301
8 (-)	Klebsiella aerogenes 15282_1 CHB	1.04	28451
9 (-)	Raoultella ornithinolytica MB_18887 CHB	1.03	54291

Tableau des résultats pour analyte 26--suite page suivante

Tableau des résultats pour analyte 26 -- suite de la page précédente			
**Classement (Qualité)**	**Profil de référence**	**Score Valeur**	**Identifiant NCBI**
10<br>(-)	Streptomyces sp HKI 77 HKJ	1.03	1883

Analyte 27

**Nom de l'échantillon:**	C3
**Description de l'échantillon:**	
**ID de l'échantillon:**	C3
**Date/Heure de création de l'échantillon:**	2019-07-10T18:42:16.616
**Type de l'échantillon:**	Échantillon standard
**Méthode de classification :**	MALDI Biotyper MSP Identification Standard Method 1.1
**Méthode de prétraitement :**	BioTyper Preprocessing Standard Method 1.2
**Méthode ACQ :**	D:\Methods\flexControlMethods\MBT_FC.par
**Horodatage ACQ :**	2019-07-10T18:52:28.513
**Méthode AutoXecute :**	MBT_AutoX_smart
**Bibliothèque de MSP utilisée:**	BDAL / contains 7854 MSPs / e7ef41ca-b750-4d47-9a1c-6c26fa454356 / 2019-01-02T15:31:15.698, Timone / de47ac8d-677c-4f70-821a-4bf7f4ccfa8d / 2019-01-22T15:40:50.648, Culturomics / 89878d5c-559e-4a65-96a9-6a526c01a7ee / 2019-06-28T13:49:07.074

Classement (Qualité)	Profil de référence	Score Valeur	Identifiant NCBI
1 (+++)	Bacillus pumilus CSURP4226	2.43	131944301
2 (+++)	Bacillus pumilus CSURP8100	2.20	131944301
3 (+++)	Bacillus pumilus CSURP4085	2.12	131944301
4 (+)	Bacillus pumilus 10403987	1.91	147143335
5 (+)	Bacillus pumilus 10403329	1.91	147143335
6 (+)	Bacillus pumilus 10403607	1.89	147143335
7 (+)	Bacillus pumilus 10403990	1.86	147143335
8 (+)	Bacillus pumilus CSURP4105	1.85	131944301
9 (+)	Bacillus pumilus 10403751	1.82	147143335

Tableau des résultats pour analyte 27--suite page suivante

Tableau des résultats pour analyte 27 -- suite de la page précédente			
**Classement (Qualité)**	**Profil de référence**	**Score Valeur**	**Identifiant NCBI**
10<br>(+)	Bacillus pumilus CSURP6343	1.76	131944301

Analyte 28

**Nom de l'échantillon:**	C4
**Description de l'échantillon:**	
**ID de l'échantillon:**	C4
**Date/Heure de création de l'échantillon:**	2019-07-10T18:42:16.618
**Type de l'échantillon:**	Échantillon standard
**Méthode de classification :**	MALDI Biotyper MSP Identification Standard Method 1.1
**Méthode de prétraitement :**	BioTyper Preprocessing Standard Method 1.2
**Méthode ACQ :**	D:\Methods\flexControlMethods\MBT_FC.par
**Horodatage ACQ :**	2019-07-10T18:52:51.267
**Méthode AutoXecute :**	MBT_AutoX_smart
**Bibliothèque de MSP utilisée:**	BDAL / contains 7854 MSPs / e7ef41ca-b750-4d47-9a1c-6c26fa454356 / 2019-01-02T15:31:15.698, Timone / de47ac8d-677c-4f70-821a-4bf7f4ccfa8d / 2019-01-22T15:40:50.648, Culturomics / 89878d5c-559e-4a65-96a9-6a526c01a7ee / 2019-06-28T13:49:07.074

Classement (Qualité)	Profil de référence	Score Valeur	Identifiant NCBI
1 (+)	Bacillus pumilus CSURP4226	1.92	131944301
2 (+)	Bacillus pumilus CSURP4085	1.77	131944301
3 (+)	Bacillus pumilus CSURP6343	1.74	131944301
4 (+)	Bacillus pumilus CSURP6343	1.74	131944301
5 (+)	Bacillus pumilus 10403329	1.72	147143335
6 (-)	Bacillus pumilus CSURP4105	1.65	131944301
7 (-)	Bacillus pumilus 10403990	1.64	147143335
8 (-)	Bacillus pumilus 10403987	1.62	147143335
9 (-)	Bacillus pumilus 10403985	1.54	147143335

Tableau des résultats pour analyte 28--suite page suivante

Tableau des résultats pour analyte 28 -- suite de la page précédente			
**Classement (Qualité)**	**Profil de référence**	**Score Valeur**	**Identifiant NCBI**
10 (-)	Bacillus pumilus 10403206	1.52	147143335

Analyte 29

**Nom de l'échantillon:**	C5
**Description de l'échantillon:**	
**ID de l'échantillon:**	C5
**Date/Heure de création de l'échantillon:**	2019-07-10T18:42:16.620
**Type de l'échantillon:**	Échantillon standard
**Méthode de classification :**	MALDI Biotyper MSP Identification Standard Method 1.1
**Méthode de prétraitement :**	BioTyper Preprocessing Standard Method 1.2
**Méthode ACQ :**	D:\Methods\flexControlMethods\MBT_FC.par
**Horodatage ACQ :**	2019-07-10T18:53:12.527
**Méthode AutoXecute :**	MBT_AutoX_smart
**Bibliothèque de MSP utilisée:**	BDAL / contains 7854 MSPs / e7ef41ca-b750-4d47-9a1c-6c26fa454356 / 2019-01-02T15:31:15.698, Timone / de47ac8d-677c-4f70-821a-4bf7f4ccfa8d / 2019-01-22T15:40:50.648, Culturomics / 89878d5c-559e-4a65-96a9-6a526c01a7ee / 2019-06-28T13:49:07.074

Classement (Qualité)	Profil de référence	Score Valeur	Identifiant NCBI
1 (+++)	Staphylococcus lentus CSURP4063	2.17	131944301
2 (+++)	Staphylococcus lentus CSURP3462	2.13	131944301
3 (+++)	Staphylococcus lentus CSURP4536	2.10	131944301
4 (-)	Lactobacillus vini DSM 20605T DSM	1.43	238015
5 (-)	Lactobacillus paracasei ssp paracasei DSM 8742 DSM	1.43	47714
6 (-)	Clostridium cochlearium 1077_ATCC 17787T BOG	1.33	1494
7 (-)	Bacillus pumilus 10403987	1.22	147143335
8 (-)	Staphylococcus epidermidis CSURP5596	1.21	131944301
9 (-)	Lactobacillus curvatus DSM 20495 DSM	1.20	28038

Tableau des résultats pour analyte 29--suite page suivante

Tableau des résultats pour analyte 29 -- suite de la page précédente			
**Classement (Qualité)**	**Profil de référence**	**Score Valeur**	**Identifiant NCBI**
10<br>(-)	Lachnoclostridium caccae CSURP3244	1.20	131944301

Analyte 30

**Nom de l’échantillon:**	C6
**Description de l’échantillon:**	
**ID de l’échantillon:**	C6
**Date/Heure de création de l’échantillon:**	2019-07-10T18:42:16.622
**Type de l’échantillon:**	Échantillon standard
**Méthode de classification :**	MALDI Biotyper MSP Identification Standard Method 1.1
**Méthode de prétraitement :**	BioTyper Preprocessing Standard Method 1.2
**Méthode ACQ :**	D:\Methods\flexControlMethods\MBT_FC.par
**Horodatage ACQ :**	2019-07-10T18:53:31.382
**Méthode AutoXecute :**	MBT_AutoX_smart
**Bibliothèque de MSP utilisée:**	BDAL / contains 7854 MSPs / e7ef41ca-b750-4d47-9a1c-6c26fa454356 / 2019-01-02T15:31:15.698, Timone / de47ac8d-677c-4f70-821a-4bf7f4ccfa8d / 2019-01-22T15:40:50.648, Culturomics / 89878d5c-559e-4a65-96a9-6a526c01a7ee / 2019-06-28T13:49:07.074

Classement (Qualité)	Profil de référence	Score Valeur	Identifiant NCBI
1 (+++)	Staphylococcus lentus CSURP3462	2.41	131944301
2 (+++)	Staphylococcus lentus CSURP4063	2.28	131944301
3 (+++)	Staphylococcus lentus CSURP4536	2.15	131944301
4 (-)	Staphylococcus sciuri ssp sciuri DSM 20345T DSM	1.48	147467
5 (-)	Lactobacillus paracasei ssp paracasei DSM 8742 DSM	1.46	47714
6 (-)	Lactobacillus vini DSM 20605T DSM	1.42	238015
7 (-)	Lactobacillus agilis DSM 20510 DSM	1.31	1601
8 (-)	Staphylococcus aureus ATCC 29213 THL	1.29	1280
9 (-)	Lactobacillus paracasei ssp paracasei DSM 2649 DSM	1.28	47714

Tableau des résultats pour analyte 30--suite page suivante

Tableau des résultats pour analyte 30 -- suite de la page précédente			
**Classement (Qualité)**	**Profil de référence**	**Score Valeur**	**Identifiant NCBI**
10<br>(-)	Morganella morganii ssp sibonii Mb19277_2 CHB	1.28	180435

Analyte 31

**Nom de l'échantillon:**	C7
**Description de l'échantillon:**	
**ID de l'échantillon:**	C7
**Date/Heure de création de l'échantillon:**	2019-07-10T18:42:16.623
**Type de l'échantillon:**	Échantillon standard
**Méthode de classification :**	MALDI Biotyper MSP Identification Standard Method 1.1
**Méthode de prétraitement :**	BioTyper Preprocessing Standard Method 1.2
**Méthode ACQ :**	D:\Methods\flexControlMethods\MBT_FC.par
**Horodatage ACQ :**	2019-07-10T18:53:50.773
**Méthode AutoXecute :**	MBT_AutoX_smart
**Bibliothèque de MSP utilisée:**	BDAL / contains 7854 MSPs / e7ef41ca-b750-4d47-9a1c-6c26fa454356 / 2019-01-02T15:31:15.698, Timone / de47ac8d-677c-4f70-821a-4bf7f4ccfa8d / 2019-01-22T15:40:50.648, Culturomics / 89878d5c-559e-4a65-96a9-6a526c01a7ee / 2019-06-28T13:49:07.074

Classement (Qualité)	Profil de référence	Score Valeur	Identifiant NCBI
1 (+++)	Bacillus pumilus CSURP4226	2.19	131944301
2 (+++)	Bacillus pumilus CSURP8100	2.17	131944301
3 (+++)	Bacillus pumilus CSURP4105	2.10	131944301
4 (+++)	Bacillus pumilus CSURP4085	2.01	131944301
5 (+)	Bacillus pumilus 10403329	1.88	147143335
6 (+)	Bacillus pumilus 10149151	1.82	147143335
7 (+)	Bacillus pumilus CSURP505	1.81	131944301
8 (+)	Bacillus pumilus CSURP6343	1.80	131944301
9 (+)	Bacillus pumilus CSURP6343	1.80	131944301

Tableau des résultats pour analyte 31--suite page suivante

Tableau des résultats pour analyte 31 -- suite de la page précédente			
**Classement (Qualité)**	**Profil de référence**	**Score Valeur**	**Identifiant NCBI**
10<br>(+)	Bacillus pumilus 10403607	1.79	147143335

Analyte 32

**Nom de l'échantillon:**	C8
**Description de l'échantillon:**	
**ID de l'échantillon:**	C8
**Date/Heure de création de l'échantillon:**	2019-07-10T18:42:16.625
**Type de l'échantillon:**	Échantillon standard
**Méthode de classification :**	MALDI Biotyper MSP Identification Standard Method 1.1
**Méthode de prétraitement :**	BioTyper Preprocessing Standard Method 1.2
**Méthode ACQ :**	D:\Methods\flexControlMethods\MBT_FC.par
**Horodatage ACQ :**	2019-07-10T18:54:12.104
**Méthode AutoXecute :**	MBT_AutoX_smart
**Bibliothèque de MSP utilisée:**	BDAL / contains 7854 MSPs / e7ef41ca-b750-4d47-9a1c-6c26fa454356 / 2019-01-02T15:31:15.698, Timone / de47ac8d-677c-4f70-821a-4bf7f4ccfa8d / 2019-01-22T15:40:50.648, Culturomics / 89878d5c-559e-4a65-96a9-6a526c01a7ee / 2019-06-28T13:49:07.074

Classement (Qualité)	Profil de référence	Score Valeur	Identifiant NCBI
1 (+)	Bacillus pumilus 10403987	1.78	147143335
2 (+)	Bacillus pumilus CSURP4085	1.74	131944301
3 (+)	Bacillus pumilus CSURP4226	1.73	131944301
4 (+)	Bacillus pumilus CSURP6343	1.72	131944301
5 (+)	Bacillus pumilus CSURP6343	1.72	131944301
6 (-)	Bacillus pumilus 10403607	1.68	147143335
7 (-)	Bacillus pumilus CSURP8100	1.59	131944301
8 (-)	Bacillus pumilus 10403990	1.57	147143335
9 (-)	Bacillus pumilus 10403329	1.56	147143335

Tableau des résultats pour analyte 32--suite page suivante

Tableau des résultats pour analyte 32 -- suite de la page précédente			
**Classement (Qualité)**	**Profil de référence**	**Score Valeur**	**Identifiant NCBI**
10<br>(-)	Bacillus pumilus CSURP4105	1.56	131944301

Analyte 33

**Nom de l'échantillon:**	C9
**Description de l'échantillon:**	
**ID de l'échantillon:**	C9
**Date/Heure de création de l'échantillon:**	2019-07-10T18:42:16.627
**Type de l'échantillon:**	Échantillon standard
**Méthode de classification :**	MALDI Biotyper MSP Identification Standard Method 1.1
**Méthode de prétraitement :**	BioTyper Preprocessing Standard Method 1.2
**Méthode ACQ :**	D:\Methods\flexControlMethods\MBT_FC.par
**Horodatage ACQ :**	2019-07-10T18:54:33.485
**Méthode AutoXecute :**	MBT_AutoX_smart
**Bibliothèque de MSP utilisée:**	BDAL / contains 7854 MSPs / e7ef41ca-b750-4d47-9a1c-6c26fa454356 / 2019-01-02T15:31:15.698, Timone / de47ac8d-677c-4f70-821a-4bf7f4ccfa8d / 2019-01-22T15:40:50.648, Culturomics / 89878d5c-559e-4a65-96a9-6a526c01a7ee / 2019-06-28T13:49:07.074

Classement (Qualité)	Profil de référence	Score Valeur	Identifiant NCBI
1 (+++)	Bacillus subtilis CSURP291	2.35	131944301
2 (+++)	Bacillus subtilis DSM 5611 DSM	2.26	1423
3 (+++)	Bacillus subtilis ssp subtilis DSM 5660 DSM	2.11	135461
4 (+++)	Lactobacillus fermentum CSURP4362	2.09	131944301
5 (+++)	Bacillus vallismortis CSURP984	2.04	131944301
6 (+++)	Bacillus subtilis DSM 5552 DSM	2.04	1423
7 (+++)	Bacillus subtilis ssp subtilis DSM 10T DSM	2.03	135461
8 (+++)	Bacillus mojavensis CSURP1524	2.02	131944301
9 (+++)	Bacillus subtilis CSURP3865	2.02	131944301

Tableau des résultats pour analyte 33--suite page suivante

Classement (Qualité)	Profil de référence	Score Valeur	Identifiant NCBI
Tableau des résultats pour analyte 33 -- suite de la page précédente			
10 (+++)	Bacillus mojavensis DSM 9205T DSM	2.01	72360

Analyte 34

**Nom de l'échantillon:**	C10
**Description de l'échantillon:**	
**ID de l'échantillon:**	C10
**Date/Heure de création de l'échantillon:**	2019-07-10T18:42:16.630
**Type de l'échantillon:**	Échantillon standard
**Méthode de classification :**	MALDI Biotyper MSP Identification Standard Method 1.1
**Méthode de prétraitement :**	BioTyper Preprocessing Standard Method 1.2
**Méthode ACQ :**	D:\Methods\flexControlMethods\MBT_FC.par
**Horodatage ACQ :**	2019-07-10T18:54:55.419
**Méthode AutoXecute :**	MBT_AutoX_smart
**Bibliothèque de MSP utilisée:**	BDAL / contains 7854 MSPs / e7ef41ca-b750-4d47-9a1c-6c26fa454356 / 2019-01-02T15:31:15.698, Timone / de47ac8d-677c-4f70-821a-4bf7f4ccfa8d / 2019-01-22T15:40:50.648, Culturomics / 89878d5c-559e-4a65-96a9-6a526c01a7ee / 2019-06-28T13:49:07.074

Classement (Qualité)	Profil de référence	Score Valeur	Identifiant NCBI
1 (+++)	Bacillus subtilis CSURP291	2.30	131944301
2 (+++)	Bacillus subtilis DSM 5611 DSM	2.15	1423
3 (+++)	Bacillus subtilis CSURP3865	2.11	131944301
4 (+++)	Bacillus subtilis ssp subtilis DSM 5660 DSM	2.07	135461
5 (+++)	Bacillus vallismortis CSURP984	2.07	131944301
6 (+++)	Bacillus subtilis ssp subtilis DSM 10T DSM	2.02	135461
7 (+++)	Bacillus mojavensis DSM 9205T DSM	2.00	72360
8 (+)	Lactobacillus fermentum CSURP4362	1.99	131944301
9 (+)	Bacillus mojavensis CSURP1524	1.97	131944301

Tableau des résultats pour analyte 34--suite page suivante

Tableau des résultats pour analyte 34 -- suite de la page précédente			
**Classement (Qualité)**	**Profil de référence**	**Score Valeur**	**Identifiant NCBI**
10<br>(+)	Bacillus subtilis DSM 5552 DSM	1.91	1423

Analyte 35

**Nom de l'échantillon:**	C11
**Description de l'échantillon:**	
**ID de l'échantillon:**	C11
**Date/Heure de création de l'échantillon:**	2019-07-10T18:42:16.632
**Type de l'échantillon:**	Échantillon standard
**Méthode de classification :**	MALDI Biotyper MSP Identification Standard Method 1.1
**Méthode de prétraitement :**	BioTyper Preprocessing Standard Method 1.2
**Méthode ACQ :**	D:\Methods\flexControlMethods\MBT_FC.par
**Horodatage ACQ :**	2019-07-10T18:55:17.159
**Méthode AutoXecute :**	MBT_AutoX_smart
**Bibliothèque de MSP utilisée:**	BDAL / contains 7854 MSPs / e7ef41ca-b750-4d47-9a1c-6c26fa454356 / 2019-01-02T15:31:15.698, Timone / de47ac8d-677c-4f70-821a-4bf7f4ccfa8d / 2019-01-22T15:40:50.648, Culturomics / 89878d5c-559e-4a65-96a9-6a526c01a7ee / 2019-06-28T13:49:07.074

Classement (Qualité)	Profil de référence	Score Valeur	Identifiant NCBI
1 (-)	Lactobacillus plantarum DSM 20205 DSM	1.46	1590
2 (-)	Clostridium cadaveris 1074_ATCC 25783T BOG	1.39	1529
3 (-)	Lactobacillus fermentum DSM 20391 DSM	1.29	1613
4 (-)	Bacillus subtilis ssp subtilis DSM 5660 DSM	1.29	135461
5 (-)	Glutamicibacter bergerei DSM 16367T DSM	1.28	256702
6 (-)	Clostridium tetani 1089_ATCC 10779 BOG	1.28	1513
7 (-)	Bacillus subtilis ssp spizizenii DSM 15029T DSM	1.28	96241
8 (-)	Bacillus koreensis DSM 16467T DSM	1.27	284581
9 (-)	Bacillus atrophaeus DSM 5551 DSM	1.26	1452

Tableau des résultats pour analyte 35--suite page suivante

Tableau des résultats pour analyte 35 -- suite de la page précédente			
**Classement (Qualité)**	**Profil de référence**	**Score Valeur**	**Identifiant NCBI**
10<br>(-)	Bacillus muralis DSM 16288T DSM	1.26	264697

Analyte 36

**Nom de l'échantillon:**	C12
**Description de l'échantillon:**	
**ID de l'échantillon:**	C12
**Date/Heure de création de l'échantillon:**	2019-07-10T18:42:16.634
**Type de l'échantillon:**	Échantillon standard
**Méthode de classification :**	MALDI Biotyper MSP Identification Standard Method 1.1
**Méthode de prétraitement :**	BioTyper Preprocessing Standard Method 1.2
**Méthode ACQ :**	D:\Methods\flexControlMethods\MBT_FC.par
**Horodatage ACQ :**	2019-07-10T18:55:37.563
**Méthode AutoXecute :**	MBT_AutoX_smart
**Bibliothèque de MSP utilisée:**	BDAL / contains 7854 MSPs / e7ef41ca-b750-4d47-9a1c-6c26fa454356 / 2019-01-02T15:31:15.698, Timone / de47ac8d-677c-4f70-821a-4bf7f4ccfa8d / 2019-01-22T15:40:50.648, Culturomics / 89878d5c-559e-4a65-96a9-6a526c01a7ee / 2019-06-28T13:49:07.074

Classement (Qualité)	Profil de référence	Score Valeur	Identifiant NCBI
1 (+++)	Bacillus subtilis CSURP291	2.33	131944301
2 (+++)	Bacillus subtilis DSM 5611 DSM	2.17	1423
3 (+++)	Bacillus subtilis ssp subtilis DSM 5660 DSM	2.05	135461
4 (+++)	Lactobacillus fermentum CSURP4362	2.01	131944301
5 (+++)	Bacillus subtilis CSURP3865	2.00	131944301
6 (+)	Bacillus vallismortis CSURP984	1.99	131944301
7 (+)	Bacillus subtilis DSM 5552 DSM	1.96	1423
8 (+)	Bacillus subtilis ssp subtilis DSM 10T DSM	1.95	135461
9 (+)	Bacillus mojavensis DSM 9205T DSM	1.85	72360

Tableau des résultats pour analyte 36--suite page suivante

Tableau des résultats pour analyte 36 -- suite de la page précédente			
**Classement (Qualité)**	**Profil de référence**	**Score Valeur**	**Identifiant NCBI**
10<br>(+)	Bacillus mojavensis CSURP1524	1.85	131944301

Analyte 37

**Nom de l’échantillon:**	D1
**Description de l’échantillon:**	
**ID de l’échantillon:**	D1
**Date/Heure de création de l’échantillon:**	2019-07-10T18:42:16.637
**Type de l’échantillon:**	Échantillon standard
**Méthode de classification :**	MALDI Biotyper MSP Identification Standard Method 1.1
**Méthode de prétraitement :**	BioTyper Preprocessing Standard Method 1.2
**Méthode ACQ :**	D:\Methods\flexControlMethods\MBT_FC.par
**Horodatage ACQ :**	2019-07-10T18:55:57.686
**Méthode AutoXecute :**	MBT_AutoX_smart
**Bibliothèque de MSP utilisée:**	BDAL / contains 7854 MSPs / e7ef41ca-b750-4d47-9a1c-6c26fa454356 / 2019-01-02T15:31:15.698, Timone / de47ac8d-677c-4f70-821a-4bf7f4ccfa8d / 2019-01-22T15:40:50.648, Culturomics / 89878d5c-559e-4a65-96a9-6a526c01a7ee / 2019-06-28T13:49:07.074

Classement (Qualité)	Profil de référence	Score Valeur	Identifiant NCBI
1 (+)	Bacillus subtilis CSURP291	1.88	131944301
2 (+)	Bacillus subtilis DSM 5611 DSM	1.78	1423
3 (+)	Bacillus subtilis CSURP3865	1.76	131944301
4 (+)	Bacillus subtilis ssp subtilis DSM 5660 DSM	1.74	135461
5 (-)	Bacillus vallismortis CSURP984	1.69	131944301
6 (-)	Bacillus subtilis ssp subtilis DSM 10T DSM	1.61	135461
7 (-)	Bacillus subtilis DSM 5552 DSM	1.52	1423
8 (-)	Lactobacillus fermentum CSURP4362	1.52	131944301
9 (-)	Bacillus atrophaeus DSM 5551 DSM	1.43	1452

Tableau des résultats pour analyte 37--suite page suivante

Tableau des résultats pour analyte 37 -- suite de la page précédente			
**Classement (Qualité)**	**Profil de référence**	**Score Valeur**	**Identifiant NCBI**
10<br>(-)	Bacillus mojavensis DSM 9205T DSM	1.41	72360

Analyte 38

Nom de l'échantillon: D2
Description de l'échantillon:
ID de l'échantillon: D2
Date/Heure de création de l'échantillon: 2019-07-10T18:42:16.639
Type de l'échantillon: Échantillon standard
Méthode de classification : MALDI Biotyper MSP Identification Standard Method 1.1
Méthode de prétraitement : BioTyper Preprocessing Standard Method 1.2
Méthode ACQ : D:\Methods\flexControlMethods\MBT_FC.par
Horodatage ACQ : 2019-07-10T18:56:19.220
Méthode AutoXecute : MBT_AutoX_smart
Bibliothèque de MSP utilisée: BDAL / contains 7854 MSPs / e7ef41ca-b750-4d47-9a1c-6c26fa454356 / 2019-01-02T15:31:15.698, Timone / de47ac8d-677c-4f70-821a-4bf7f4ccfa8d / 2019-01-22T15:40:50.648, Culturomics / 89878d5c-559e-4a65-96a9-6a526c01a7ee / 2019-06-28T13:49:07.074

Classement (Qualité)	Profil de référence	Score Valeur	Identifiant NCBI
1 (+)	Bacillus subtilis CSURP291	1.93	131944301
2 (-)	Bacillus subtilis DSM 5611 DSM	1.56	1423
3 (-)	Bacillus valismortis CSURP2348	1.51	131944301
4 (-)	Bacillus amyloliquefaciens 10403754	1.51	147143335
5 (-)	Bacillus amyloliquefaciens 10403754	1.51	147143335
6 (-)	Lactobacillus fermentum CSURP4362	1.50	131944301
7 (-)	Moraxella catarrhalis 15 PIM	1.39	480
8 (-)	Bacillus vallismortis CSURP984	1.37	131944301
9 (-)	Ruminococcus torques P4673P	1.29	131944301

Tableau des résultats pour analyte 38--suite page suivante

Tableau des résultats pour analyte 38 -- suite de la page précédente			
**Classement (Qualité)**	**Profil de référence**	**Score Valeur**	**Identifiant NCBI**
10<br>(-)	Bacillus subtilis 10402717	1.26	147143335

Analyte 39

**Nom de l'échantillon:**	D3
**Description de l'échantillon:**	
**ID de l'échantillon:**	D3
**Date/Heure de création de l'échantillon:**	2019-07-10T18:42:16.641
**Type de l'échantillon:**	Échantillon standard
**Méthode de classification :**	MALDI Biotyper MSP Identification Standard Method 1.1
**Méthode de prétraitement :**	BioTyper Preprocessing Standard Method 1.2
**Méthode ACQ :**	D:\Methods\flexControlMethods\MBT_FC.par
**Horodatage ACQ :**	2019-07-10T18:56:33.780
**Méthode AutoXecute :**	MBT_AutoX_smart
**Bibliothèque de MSP utilisée:**	BDAL / contains 7854 MSPs / e7ef41ca-b750-4d47-9a1c-6c26fa454356 / 2019-01-02T15:31:15.698, Timone / de47ac8d-677c-4f70-821a-4bf7f4ccfa8d / 2019-01-22T15:40:50.648, Culturomics / 89878d5c-559e-4a65-96a9-6a526c01a7ee / 2019-06-28T13:49:07.074

Classement (Qualité)	Profil de référence	Score Valeur	Identifiant NCBI
1 (+++)	Bacillus pumilus CSURP4226	2.33	131944301
2 (+++)	Bacillus pumilus CSURP8100	2.32	131944301
3 (+++)	Bacillus pumilus 10403987	2.17	147143335
4 (+++)	Bacillus pumilus 10403329	2.10	147143335
5 (+++)	Bacillus pumilus 10403607	2.04	147143335
6 (+++)	Bacillus pumilus CSURP4105	2.04	131944301
7 (+++)	Bacillus pumilus 10403990	2.00	147143335
8 (+)	Bacillus pumilus CSURP4085	1.99	131944301
9 (+)	Bacillus pumilus CSURP6343	1.95	131944301

Tableau des résultats pour analyte 39--suite page suivante

Tableau des résultats pour analyte 39 -- suite de la page précédente			
**Classement (Qualité)**	**Profil de référence**	**Score Valeur**	**Identifiant NCBI**
10<br>(+)	Bacillus pumilus CSURP6343	1.95	131944301

Analyte 40

**Nom de l'échantillon:**	D4
**Description de l'échantillon:**	
**ID de l'échantillon:**	D4
**Date/Heure de création de l'échantillon:**	2019-07-10T18:42:16.644
**Type de l'échantillon:**	Échantillon standard
**Méthode de classification :**	MALDI Biotyper MSP Identification Standard Method 1.1
**Méthode de prétraitement :**	BioTyper Preprocessing Standard Method 1.2
**Méthode ACQ :**	D:\Methods\flexControlMethods\MBT_FC.par
**Horodatage ACQ :**	2019-07-10T18:56:56.157
**Méthode AutoXecute :**	MBT_AutoX_smart
**Bibliothèque de MSP utilisée:**	BDAL / contains 7854 MSPs / e7ef41ca-b750-4d47-9a1c-6c26fa454356 / 2019-01-02T15:31:15.698, Timone / de47ac8d-677c-4f70-821a-4bf7f4ccfa8d / 2019-01-22T15:40:50.648, Culturomics / 89878d5c-559e-4a65-96a9-6a526c01a7ee / 2019-06-28T13:49:07.074

Classement (Qualité)	Profil de référence	Score Valeur	Identifiant NCBI
1 (+++)	Bacillus pumilus CSURP8100	2.11	131944301
2 (+++)	Bacillus pumilus CSURP4226	2.07	131944301
3 (+)	Bacillus pumilus CSURP6343	1.95	131944301
4 (+)	Bacillus pumilus CSURP6343	1.95	131944301
5 (+)	Bacillus pumilus CSURP4105	1.92	131944301
6 (+)	Bacillus pumilus 10403329	1.91	147143335
7 (+)	Bacillus pumilus 10403607	1.88	147143335
8 (+)	Bacillus pumilus 10403987	1.80	147143335
9 (+)	Bacillus pumilus 10403985	1.79	147143335

Tableau des résultats pour analyte 40--suite page suivante

Tableau des résultats pour analyte 40 -- suite de la page précédente			
**Classement (Qualité)**	**Profil de référence**	**Score Valeur**	**Identifiant NCBI**
10<br>(+)	Bacillus pumilus CSURP4085	1.76	131944301

Analyte 41

**Nom de l'échantillon:**	D5
**Description de l'échantillon:**	
**ID de l'échantillon:**	D5
**Date/Heure de création de l'échantillon:**	2019-07-10T18:42:16.646
**Type de l'échantillon:**	Échantillon standard
**Méthode de classification :**	MALDI Biotyper MSP Identification Standard Method 1.1
**Méthode de prétraitement :**	BioTyper Preprocessing Standard Method 1.2
**Méthode ACQ :**	D:\Methods\flexControlMethods\MBT_FC.par
**Horodatage ACQ :**	2019-07-10T18:57:17.018
**Méthode AutoXecute :**	MBT_AutoX_smart
**Bibliothèque de MSP utilisée:**	BDAL / contains 7854 MSPs / e7ef41ca-b750-4d47-9a1c-6c26fa454356 / 2019-01-02T15:31:15.698, Timone / de47ac8d-677c-4f70-821a-4bf7f4ccfa8d / 2019-01-22T15:40:50.648, Culturomics / 89878d5c-559e-4a65-96a9-6a526c01a7ee / 2019-06-28T13:49:07.074

Classement (Qualité)	Profil de référence	Score Valeur	Identifiant NCBI
1 (-)	Lactobacillus paralimentarius DSM 13238T DSM	1.39	83526
2 (-)	Lactobacillus satsumensis DSM 16230T DSM	1.32	259059
3 (-)	Cryptococcus neoformans RV07_02 18 VML	1.32	5207
4 (-)	Burkholderia cenocepacia LMG 12614 HAM	1.30	95486
5 (-)	Lactobacillus paracasei ssp paracasei DSM 20207 DSM	1.28	47714
6 (-)	Clostridium baratii 1084_ATCC 25782 BOG	1.27	1561
7 (-)	Cryptococcus neoformans_var_neoformans CBS 5467 CBS	1.27	40410
8 (-)	Sinomonas atrocyanea DSM 20127T DSM	1.25	37927
9 (-)	Burkholderia seminalis VA40474_09 ERL	1.20	87882

Tableau des résultats pour analyte 41--suite page suivante

Tableau des résultats pour analyte 41 -- suite de la page précédente			
**Classement (Qualité)**	**Profil de référence**	**Score Valeur**	**Identifiant NCBI**
10<br>(-)	Lactobacillus casei DSM 20011T DSM	1.19	1582

Analyte 42

**Nom de l'échantillon:**	D6
**Description de l'échantillon:**	
**ID de l'échantillon:**	D6
**Date/Heure de création de l'échantillon:**	2019-07-10T18:42:16.648
**Type de l'échantillon:**	Échantillon standard
**Méthode de classification :**	MALDI Biotyper MSP Identification Standard Method 1.1
**Méthode de prétraitement :**	BioTyper Preprocessing Standard Method 1.2
**Méthode ACQ :**	D:\Methods\flexControlMethods\MBT_FC.par
**Horodatage ACQ :**	2019-07-10T18:57:37.347
**Méthode AutoXecute :**	MBT_AutoX_smart
**Bibliothèque de MSP utilisée:**	BDAL / contains 7854 MSPs / e7ef41ca-b750-4d47-9a1c-6c26fa454356 / 2019-01-02T15:31:15.698, Timone / de47ac8d-677c-4f70-821a-4bf7f4ccfa8d / 2019-01-22T15:40:50.648, Culturomics / 89878d5c-559e-4a65-96a9-6a526c01a7ee / 2019-06-28T13:49:07.074

Classement (Qualité)	Profil de référence	Score Valeur	Identifiant NCBI
1 (-)	Burkholderia tuberum LMG 21444T HAM	1.37	157910
2 (-)	Legionella parisiensis HL04385084	1.31	147143335
3 (-)	Burkholderia fungorum LMG 20227T HAM	1.30	134537
4 (-)	Legionella parisiensis ATCC 700174	1.27	147143335
5 (-)	Legionella parisiensis LG08182001	1.27	147143335
6 (-)	Legionella bozemanii HL04313026	1.26	147143335
7 (-)	Legionella bozemanii HL04062051	1.24	147143335
8 (-)	Legionella parisiensis HL04385019	1.24	147143335
9 (-)	Legionella parisiensis Ly66-88	1.24	147143335

Tableau des résultats pour analyte 42--suite page suivante

Tableau des résultats pour analyte 42 -- suite de la page précédente			
**Classement (Qualité)**	**Profil de référence**	**Score Valeur**	**Identifiant NCBI**
10<br>(-)	Mannheimia haemolytica DSM 5283 BRB	1.22	75985

Analyte 43

Nom de l'échantillon: D7
Description de l'échantillon:
ID de l'échantillon: D7
Date/Heure de création de l'échantillon: 2019-07-10T18:42:16.650
Type de l'échantillon: Échantillon standard
Méthode de classification : MALDI Biotyper MSP Identification Standard Method 1.1
Méthode de prétraitement : BioTyper Preprocessing Standard Method 1.2
Méthode ACQ : D:\Methods\flexControlMethods\MBT_FC.par
Horodatage ACQ : 2019-07-10T18:57:58.385
Méthode AutoXecute : MBT_AutoX_smart
Bibliothèque de MSP utilisée: BDAL / contains 7854 MSPs / e7ef41ca-b750-4d47-9a1c-6c26fa454356 / 2019-01-02T15:31:15.698, Timone / de47ac8d-677c-4f70-821a-4bf7f4ccfa8d / 2019-01-22T15:40:50.648, Culturomics / 89878d5c-559e-4a65-96a9-6a526c01a7ee / 2019-06-28T13:49:07.074

Classement (Qualité)	Profil de référence	Score Valeur	Identifiant NCBI
1 (+++)	Proteus vulgaris (PX) 22086129 MLD	2.09	585
2 (+++)	Proteus vulgaris CSURP7845	2.09	131944301
3 (+++)	Proteus hauseri CC_2400 MCW	2.02	183417
4 (+++)	Proteus vulgaris CSURP722	2.02	131944301
5 (+++)	Proteus vulgaris DSM 30119 DSM	2.00	585
6 (+)	Proteus hauseri NY_1346 MCW	1.96	183417
7 (+)	Proteus hauseri NY_1373 MCW	1.91	183417
8 (+)	Proteus penneri DSM 4544T DSM	1.87	102862
9 (+)	Proteus vulgaris LMG 5586 LMG	1.83	585

Tableau des résultats pour analyte 43--suite page suivante

Tableau des résultats pour analyte 43 -- suite de la page précédente			
**Classement (Qualité)**	**Profil de référence**	**Score Valeur**	**Identifiant NCBI**
10<br>(+)	Proteus penneri CSURP7543	1.83	131944301

Analyte 44

**Nom de l'échantillon:**	D8
**Description de l'échantillon:**	
**ID de l'échantillon:**	D8
**Date/Heure de création de l'échantillon:**	2019-07-10T18:42:16.652
**Type de l'échantillon:**	Échantillon standard
**Méthode de classification :**	MALDI Biotyper MSP Identification Standard Method 1.1
**Méthode de prétraitement :**	BioTyper Preprocessing Standard Method 1.2
**Méthode ACQ :**	D:\Methods\flexControlMethods\MBT_FC.par
**Horodatage ACQ :**	2019-07-10T18:58:19.663
**Méthode AutoXecute :**	MBT_AutoX_smart
**Bibliothèque de MSP utilisée:**	BDAL / contains 7854 MSPs / e7ef41ca-b750-4d47-9a1c-6c26fa454356 / 2019-01-02T15:31:15.698, Timone / de47ac8d-677c-4f70-821a-4bf7f4ccfa8d / 2019-01-22T15:40:50.648, Culturomics / 89878d5c-559e-4a65-96a9-6a526c01a7ee / 2019-06-28T13:49:07.074

Classement (Qualité)	Profil de référence	Score Valeur	Identifiant NCBI
1 (+)	Proteus vulgaris CSURP7845	1.77	131944301
2 (-)	Proteus vulgaris DSM 30119 DSM	1.65	585
3 (-)	Proteus vulgaris CSURP8928	1.64	131944301
4 (-)	Proteus penneri DSM 4544T DSM	1.59	102862
5 (-)	Proteus vulgaris CSURP722	1.57	131944301
6 (-)	Proteus hauseri CC_2400 MCW	1.52	183417
7 (-)	Proteus penneri CSURP7543	1.51	131944301
8 (-)	Proteus vulgaris (PX) 22086129 MLD	1.48	585
9 (-)	Proteus hauseri NY_1373 MCW	1.45	183417

Tableau des résultats pour analyte 44--suite page suivante

Tableau des résultats pour analyte 44 -- suite de la page précédente			
**Classement (Qualité)**	**Profil de référence**	**Score Valeur**	**Identifiant NCBI**
10<br>(-)	Proteus mirabilis 9482_2 CHB	1.44	584

Analyte 45

**Nom de l'échantillon:**	D9
**Description de l'échantillon:**	
**ID de l'échantillon:**	D9
**Date/Heure de création de l'échantillon:**	2019-07-10T18:42:16.654
**Type de l'échantillon:**	Échantillon standard
**Méthode de classification :**	MALDI Biotyper MSP Identification Standard Method 1.1
**Méthode de prétraitement :**	BioTyper Preprocessing Standard Method 1.2
**Méthode ACQ :**	D:\Methods\flexControlMethods\MBT_FC.par
**Horodatage ACQ :**	2019-07-10T18:58:41.943
**Méthode AutoXecute :**	MBT_AutoX_smart
**Bibliothèque de MSP utilisée:**	BDAL / contains 7854 MSPs / e7ef41ca-b750-4d47-9a1c-6c26fa454356 / 2019-01-02T15:31:15.698, Timone / de47ac8d-677c-4f70-821a-4bf7f4ccfa8d / 2019-01-22T15:40:50.648, Culturomics / 89878d5c-559e-4a65-96a9-6a526c01a7ee / 2019-06-28T13:49:07.074

Classement (Qualité)	Profil de référence	Score Valeur	Identifiant NCBI
1 (-)	Proteus vulgaris CSURP382	1.57	131944301
2 (-)	Proteus hauseri CC_2695 MCW	1.48	183417
3 (-)	Proteus vulgaris LMG 5586 LMG	1.44	585
4 (-)	Escherichia coli CSURP4715	1.43	131944301
5 (-)	Proteus hauseri NY_1346 MCW	1.41	183417
6 (-)	Escherichia coli CSURP4923	1.38	131944301
7 (-)	Escherichia coli CSURP397	1.33	131944301
8 (-)	Proteus vulgaris CSURP7845	1.33	131944301
9 (-)	Proteus mirabilis (PX) 22086112 MLD	1.32	584

Tableau des résultats pour analyte 45--suite page suivante

Tableau des résultats pour analyte 45 -- suite de la page précédente			
**Classement (Qualité)**	**Profil de référence**	**Score Valeur**	**Identifiant NCBI**
10<br>(-)	Proteus vulgaris (PX) 22086129 MLD	1.32	585

Analyte 46

Nom de l'échantillon: D10
Description de l'échantillon:
ID de l'échantillon: D10
Date/Heure de création de l'échantillon: 2019-07-10T18:42:16.656
Type de l'échantillon: Échantillon standard
Méthode de classification : MALDI Biotyper MSP Identification Standard Method 1.1
Méthode de prétraitement : BioTyper Preprocessing Standard Method 1.2
Méthode ACQ : D:\Methods\flexControlMethods\MBT_FC.par
Horodatage ACQ : 2019-07-10T18:59:02.737
Méthode AutoXecute : MBT_AutoX_smart
Bibliothèque de MSP utilisée: BDAL / contains 7854 MSPs / e7ef41ca-b750-4d47-9a1c-6c26fa454356 / 2019-01-02T15:31:15.698, Timone / de47ac8d-677c-4f70-821a-4bf7f4ccfa8d / 2019-01-22T15:40:50.648, Culturomics / 89878d5c-559e-4a65-96a9-6a526c01a7ee / 2019-06-28T13:49:07.074

Classement (Qualité)	Profil de référence	Score Valeur	Identifiant NCBI
1 (+)	Proteus hauseri CC_2695 MCW	1.96	183417
2 (+)	Proteus hauseri CC_2400 MCW	1.91	183417
3 (+)	Proteus vulgaris DSM 46228 DSM	1.90	585
4 (+)	Proteus penneri CSURP7543	1.85	131944301
5 (+)	Proteus vulgaris DSM 30119 DSM	1.84	585
6 (+)	Proteus vulgaris DSM 13625 DSM	1.83	585
7 (+)	Proteus vulgaris CSURP7845	1.82	131944301
8 (+)	Proteus vulgaris DSM 13387_QC DSM	1.81	585
9 (+)	Proteus vulgaris CSURP722	1.81	131944301

Tableau des résultats pour analyte 46--suite page suivante

Tableau des résultats pour analyte 46 -- suite de la page précédente			
**Classement (Qualité)**	**Profil de référence**	**Score Valeur**	**Identifiant NCBI**
10 (+)	Proteus vulgaris LMG 5586 LMG	1.80	585

Analyte 47

**Nom de l'échantillon:**	D11
**Description de l'échantillon:**	
**ID de l'échantillon:**	D11
**Date/Heure de création de l'échantillon:**	2019-07-10T18:42:16.658
**Type de l'échantillon:**	Échantillon standard
**Méthode de classification :**	MALDI Biotyper MSP Identification Standard Method 1.1
**Méthode de prétraitement :**	BioTyper Preprocessing Standard Method 1.2
**Méthode ACQ :**	D:\Methods\flexControlMethods\MBT_FC.par
**Horodatage ACQ :**	2019-07-10T18:59:23.020
**Méthode AutoXecute :**	MBT_AutoX_smart
**Bibliothèque de MSP utilisée:**	BDAL / contains 7854 MSPs / e7ef41ca-b750-4d47-9a1c-6c26fa454356 / 2019-01-02T15:31:15.698, Timone / de47ac8d-677c-4f70-821a-4bf7f4ccfa8d / 2019-01-22T15:40:50.648, Culturomics / 89878d5c-559e-4a65-96a9-6a526c01a7ee / 2019-06-28T13:49:07.074

Classement (Qualité)	Profil de référence	Score Valeur	Identifiant NCBI
1 (+)	Morganella morganii (PX) 22086121 MLD	1.80	582
2 (+)	Morganella morganii ssp morganii DSM 30117 DSM	1.78	180434
3 (+)	Morganella morganii 9544_1 CHB	1.71	582
4 (-)	Morganella morganii (E) 21086317 MLD	1.67	582
5 (-)	Morganella morganii ssp morganii 15284_1 CHB	1.66	180434
6 (-)	Morganella morganii CSURP4159	1.65	131944301
7 (-)	Morganella morganii ssp morganii DSM 30164T DSM	1.56	180434
8 (-)	Morganella morganii 451 RLT	1.54	582
9 (-)	Morganella morganii RV_BA_03_A LBK	1.49	582

Tableau des résultats pour analyte 47--suite page suivante

Tableau des résultats pour analyte 47 -- suite de la page précédente			
**Classement (Qualité)**	**Profil de référence**	**Score Valeur**	**Identifiant NCBI**
10<br>(-)	Proteus vulgaris (PX) 22086129 MLD	1.45	585

Analyte 48

**Nom de l'échantillon:**	D12
**Description de l'échantillon:**	
**ID de l'échantillon:**	D12
**Date/Heure de création de l'échantillon:**	2019-07-10T18:42:16.660
**Type de l'échantillon:**	Échantillon standard
**Méthode de classification :**	MALDI Biotyper MSP Identification Standard Method 1.1
**Méthode de prétraitement :**	BioTyper Preprocessing Standard Method 1.2
**Méthode ACQ :**	D:\Methods\flexControlMethods\MBT_FC.par
**Horodatage ACQ :**	2019-07-10T18:59:44.702
**Méthode AutoXecute :**	MBT_AutoX_smart
**Bibliothèque de MSP utilisée:**	BDAL / contains 7854 MSPs / e7ef41ca-b750-4d47-9a1c-6c26fa454356 / 2019-01-02T15:31:15.698, Timone / de47ac8d-677c-4f70-821a-4bf7f4ccfa8d / 2019-01-22T15:40:50.648, Culturomics / 89878d5c-559e-4a65-96a9-6a526c01a7ee / 2019-06-28T13:49:07.074

Classement (Qualité)	Profil de référence	Score Valeur	Identifiant NCBI
1 (+)	Morganella morganii 9544_1 CHB	1.89	582
2 (+)	Morganella morganii (E) 21086317 MLD	1.83	582
3 (+)	Morganella morganii ssp morganii DSM 30164T DSM	1.83	180434
4 (+)	Morganella morganii ssp morganii DSM 30117 DSM	1.79	180434
5 (+)	Morganella morganii (PX) 22086121 MLD	1.78	582
6 (+)	Morganella morganii ssp morganii 15284_1 CHB	1.75	180434
7 (+)	Morganella morganii CSURP4159	1.73	131944301
8 (-)	Morganella morganii CSURP8059	1.61	131944301
9 (-)	Morganella morganii RV_BA_03_A LBK	1.60	582

Tableau des résultats pour analyte 48--suite page suivante

Tableau des résultats pour analyte 48 -- suite de la page précédente			
**Classement (Qualité)**	**Profil de référence**	**Score Valeur**	**Identifiant NCBI**
10<br>(-)	Proteus vulgaris (PX) 22086129 MLD	1.56	585
